# Identification of DC-174 as a Novel Hydroxamic Precandidate for the Development of an Oral Snakebite Treatment

**DOI:** 10.1021/acs.jmedchem.6c00064

**Published:** 2026-04-09

**Authors:** Daniel J. W. Chong, Laura-Oana Albulescu, Adam Westhorpe, Rachel H. Clare, Amy E. Marriott, Christopher M. Woodley, Ramachandran Gunasekar, Nada Mosallam, Edouard Crittenden, Emma Stars, Charlotte Dawson, Jeroen Kool, Mark C. Wilkinson, Suet C. Leung, Neil G. Berry, Nicholas R. Casewell, Paul M. O’Neill

**Affiliations:** † Department of Chemistry, 4591 University of Liverpool, Grove Street, Liverpool L69 7ZD, U. K.; ‡ Centre for Snakebite Research & Interventions, 9655 Liverpool School of Tropical Medicine, Pembroke Place, Liverpool L3 5QA, U. K.; § Department of Chemistry and Pharmaceutical Sciences, Amsterdam Institute for Molecular and Life Science, 1190 Vrije Universiteit Amsterdam, De Boelelaan 1105, Amsterdam 1081 HV, The Netherlands

## Abstract

Snakebite envenoming is a neglected tropical disease that causes high mortality and morbidity. The current treatment, intravenous antivenom, comes with numerous disadvantages, making new therapeutics important. Optimized small molecules offer the possibility for oral use at the onset of envenoming, and the highly pathogenic, zinc-dependent snake venom metalloproteinase toxin family represents an attractive target for drug discovery. Through systematic chemical modification guided by molecular modeling, we describe the development of hydroxamic acid **23** (DC-174), a molecule that displays potent broad-spectrum metalloproteinase inhibition (IC50s < 10 nM) and neutralizes the procoagulant activities of multiple snake venoms. In oral dosing studies, **23** showed preclinical efficacy in a mouse model of severe envenoming, with efficacy boosted by a pharmacokinetically informed multiple dosing regimen. This rationally designed, orally bioavailable metalloproteinase inhibitor represents an excellent lead compound for the development of a small-molecule drug treatment for snakebite.

## Graphical abstract

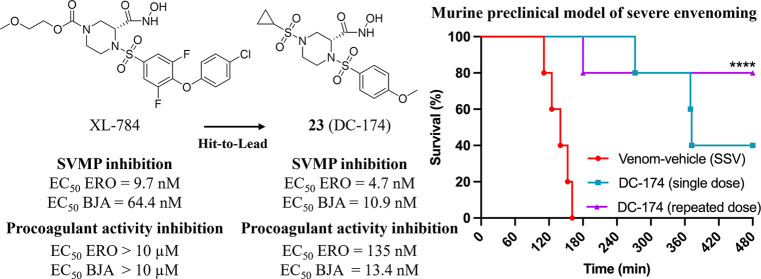

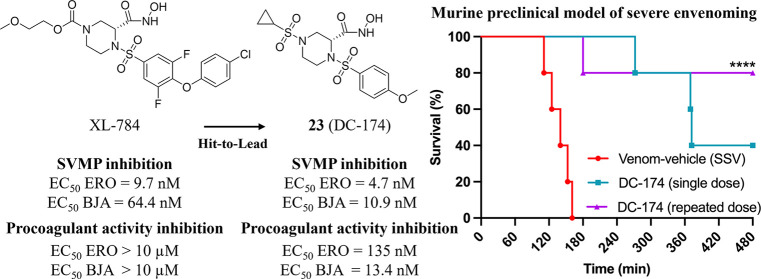

## Introduction

Snakebite envenoming is a life-threatening condition predominantly affecting people in rural communities in Africa, Asia and Latin America, and results in an annual death toll of up to 138,000, with many more survivors suffering long-term morbidity.^1^ In 2017 the World Health Organization (WHO) reclassified snakebite envenoming as a priority Neglected Tropical Disease^2^ and a roadmap was developed to reduce the snakebite burden, with one key pillar of this strategy being to ensure access to safe and effective treatment, inclusive of improved therapeutics.^3^

Antivenoms are current standard of care snakebite treatments that consist of animal-derived polyclonal antibodies sourced from hyper-immunized animals. Despite saving lives, antivenoms have several limitations, including the requirement for administration in a clinical environment due to the necessity for intravenous delivery, management of adverse reactions and reliance on cold chain storage.^4^ Placing these constraints on patients who are often on average 6 h from a hospital setting,^5,6^ likely contributes to poor outcomes, given the often acute and life-threatening nature of snakebite. Additional challenges include the low affordability and accessibility of antivenom in many tropical regions, which is partly due to the necessity to manufacture different products for different regions, due to the complex and variable nature of venom toxins among snake species.^7^

Despite extensive venom variation, four main pathogenic toxin families have been identified as being of greatest importance for snakebite due to their relative abundance and toxicity; the snake venom metalloproteinases (SVMPs), snake venom serine proteases (SVSPs), phospholipases A_2_ (PLA_2_s) and three-finger toxins (3FTxs).^8^ The SVMPs are zinc-dependent enzymes present in the venom of most snakes, but are particularly abundant in viper venoms (*Viperidae*).^8,9^ SVMPs are divided into three subclasses (P–I, P–II, P–III) based on gene organization; though each contains structurally and functionally distinct toxin isoforms.^9^ SVMPs induce and prolong local and systemic hemorrhage by degrading extracellular matrix proteins resulting in microvascular damage and by cleaving blood clotting factors, such as fibrinogen and prothrombin, resulting in coagulopathy.^10,11^ Their high abundance in many viper venoms (up to 74% of all toxins^8^) makes SVMPs a highly attractive target for novel snakebite therapeutics.

One contemporary approach (among others^12^) recently applied to more broadly inhibit diverse toxin isoforms found across snake species has been the use of small molecule drugs.^13–17^ Such molecules possess several theoretical advantages over current antivenom, including improved cross-snake species neutralization, increased affordability and no requirement for cold-chain, but also offer the possibility of oral formulation for rapid administration in the community after a snakebite, thus reducing the critical time to treatment.^18^ Recently, several drugs that may generically target the active site of venom enzymes have been actively repurposed for snakebite, including the PLA_2_ inhibitor varespladib (**1a**), which showed impressive preclinical efficacy and recently completed a Phase II snakebite trial, though the primary end point was not met.^19,20^ Similarly, two distinct group of molecules have been reported to show promising SVMP-inhibitory activities. Metal chelating molecules, particularly 2,3-dimercapto-1-propanesulfonic acid (DMPS) (**1b**), showed preclinical protection in *in vivo* envenoming models^14,16^ and has since been used in a Phase I dose optimization study to support onward clinical development for snakebite indication.^21^ The second comprises several matrix metalloproteinase (MMP) inhibitors. These compounds feature a zinc-binding motif which directly engages with the active site of SVMPs,^22^ and include marimastat (**1c**),^15,16,23–26^ prinomastat (**1d**)^24,26,27^ and batimastat (**1e**).^23–26^ These hydroxamic acids demonstrated potent inhibition of SVMP toxins *in vitro*, inhibition of procoagulant venom effects in physiologically relevant bioassays, and showed variable promise in preclinical trials for mitigating the local and systemic hemotoxic effects of snake venoms in small animal models.^15,16,23,24^

While such repurposing efforts have been highly encouraging, our understanding of the chemical space around SVMP inhibitors remains largely unexplored, as the identified repurposed drugs have not been optimized via medicinal chemistry approaches to improve their drugability for snakebite indication. To address this issue and expand the snakebite drug portfolio, we recently described a high-throughput screening (HTS) campaign that identified several potent SVMP inhibitors from a ∼3,500 compound library, including the MMP inhibitor XL-784 (**1f**), which displayed double digit nanomolar inhibition of the SVMP activity of several diverse snake venoms.^26^ Using **1f** as a starting scaffold, here we describe the first snakebite specific medicinal chemistry campaign to identify lead SVMP inhibitors that display broad cross-snake species inhibitory activity, and with superior potency and drug metabolism and pharmacokinetic (DMPK) properties to current lead drugs. Following a rationally designed optimization cascade, consisting of enzymatic and phenotypic assessments coupled to DMPK analyses, DC-174 (**23**) was identified as a precandidate molecule. This first, rationally designed, snakebite drug has desirable physicochemical and broad-spectrum venom inhibitory properties and demonstrates impressive preclinical efficacy against snakebite when dosed orally in a murine rescue model of envenoming.

## Results

Our prior primary HTS campaign identified several strong hits, including the known MMP-inhibitors marimastat (**1c**), prinomastat (**1d**) and XL-784 (**1f**).^26^ XL-784 (**1f**) has a highly modifiable piperazine scaffold and consists of (1) primary scaffold, (2) 4-substituted aryl groups and (3) *N*1-side chain (Figure 1A). Based on previous structure–activity relationship (SAR) studies with human MMPs, we preserved the central core scaffold in our SAR optimization strategy. In the related MMP-inhibitor Ro-1130830 (**1g**, Figure 1B) and its analogues, the hydroxamic acid is shown to chelate the zinc while forming a hydrogen bond with a nearby glutamate residue,^28^ with further hydrogen bonds predicted between the linking sulfonamide and backbone NH groups of a nearby loop.^29,30^ The aromatic rings represent the S1′ binding substituent (Figure 1C) shown in the development of selective MMP13 inhibitors.^28^ X-ray crystal structures of multiple SVMP toxins suggest this region and its available volume for binding may be variable (Figure S1), therefore modifications may affect the spectrum of SVMPs inhibited. Consequently, variation of substituents of the aromatic-ring and N1-side chain subunits were performed to investigate the structure–activity relationship against SVMPs.

**Figure 1:**
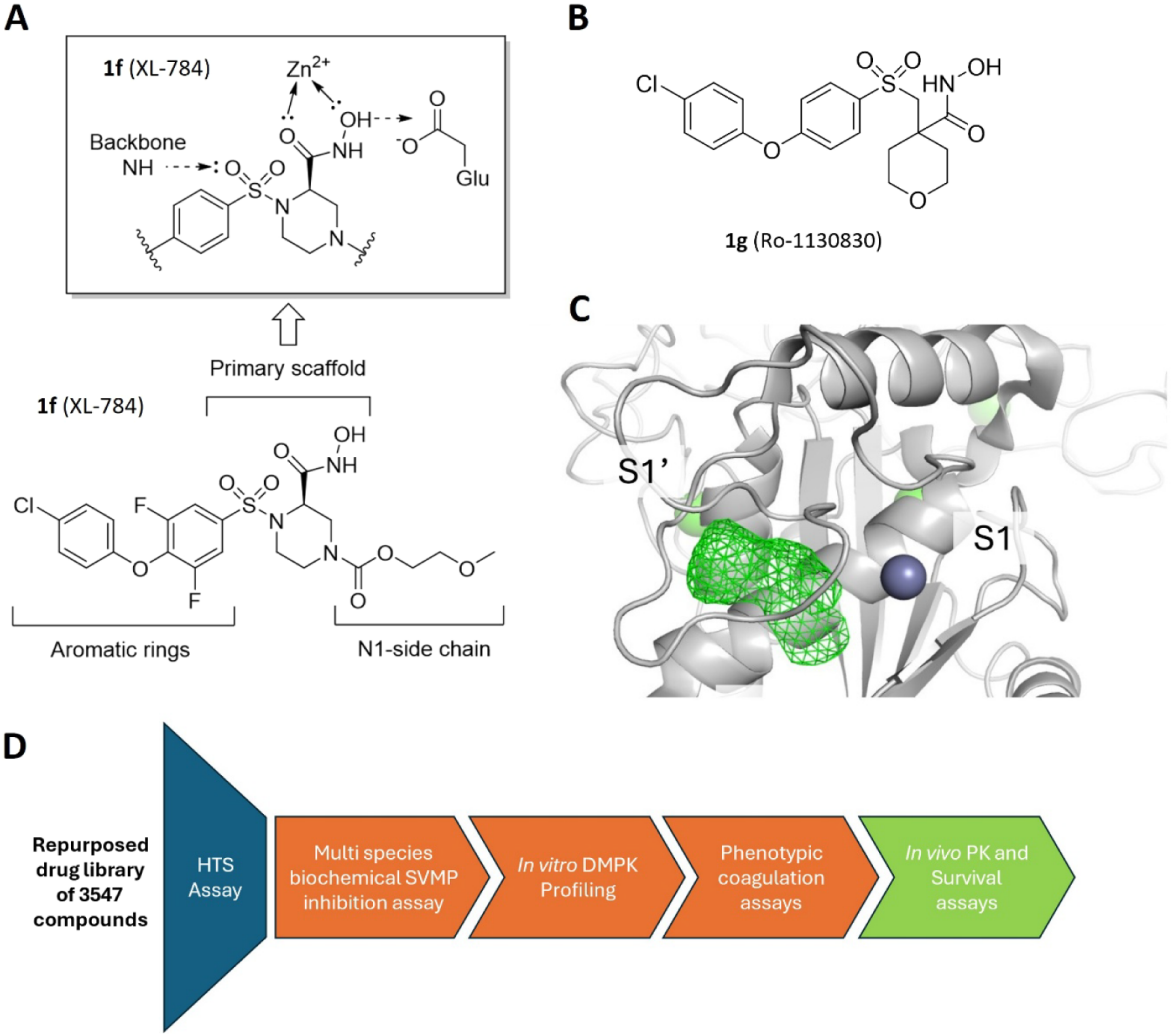
Medicinal chemistry optimization strategy. (**A**) Structure of the HTS hit compound XL-784 and proposed binding mode of the headgroup from studies using human MMP inhibitors. (**B**) The chemical structure of MMP-inhibitor Ro-1130830 (**1g**). (**C**) Representation of the binding site of the SVMP bothropasin from *Bothrops jararaca*. The protein secondary structure is rendered as gray ribbon, the catalytic zinc a as dark blue sphere and the S1′ pocket as green mesh with binding regions (S1 and S1′ pockets) labeled. (**D**) The screening cascade for compounds produced in this study.

Throughout our medicinal chemistry campaign, compounds were evaluated for their ability to inhibit enzymatic SVMP toxin activity *in vitro* using a previously described fluorescent-based substrate cleavage assay^26^ against five medically important viper venoms, namely *Echis romani* (Roman’s carpet viper, West Africa), *Crotalus atrox* (Western diamondback rattlesnake, North America), *Calloselasma rhodostoma* (Malayan pit viper, South East Asia), *Bothrops jararaca* (jararaca, South America) and *Bitis arietans* (puff adder, Africa). We defined a potent inhibitor as having *in vitro* SVMP inhibition potency of less than 100 nM. The downstream testing cascade (Figure 1D) included assessment of *in vitro* DMPK properties of the compounds, with thresholds for “good” compounds defined as rat hepatocyte clearance <10 μL/min/10^6^ cells, human microsomal clearance <10 μL/min/mg intrinsic clearances, and aqueous solubility at pH 7.4 > 500 μM.^31,32^ Secondary inhibitory assays assessing neutralization of venom activity on plasma clotting were used to triage synthesized molecules and benchmark against prinomastat and XL-784, before progression of lead compounds into *in vivo* models to evaluate pharmacokinetic (PK) and efficacy profiles.

The medicinal chemistry campaign resulted in 12 compounds (Figure 2, Table 1). The synthetic route diverged to afford either a biphenyl ether or biphenyl scaffold. Optimization began with sulfonamide extension of *N*-boc-piperazine **1**, giving yields of 67–95%. Following TFA deprotection, N-alkylation with various aliphatic and aromatic groups produced esters **6–14**. Initial conversion of these esters to hydroxamic acids via amide coupling with THP-protected hydroxylamine was later replaced by a more efficient direct conversion using excess hydroxylamine, affording hydroxamic acids **15–23**.

**Table 1:**
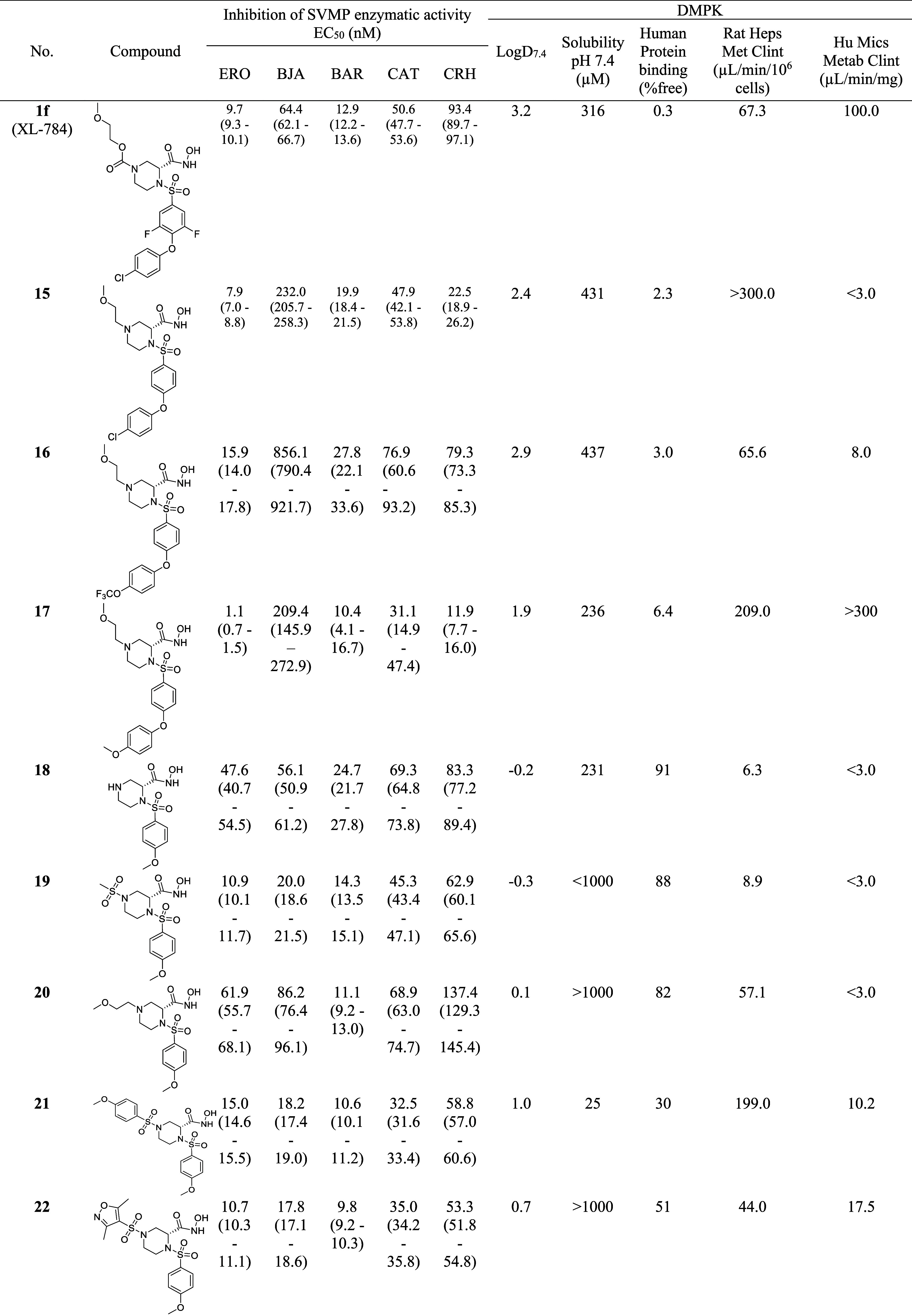

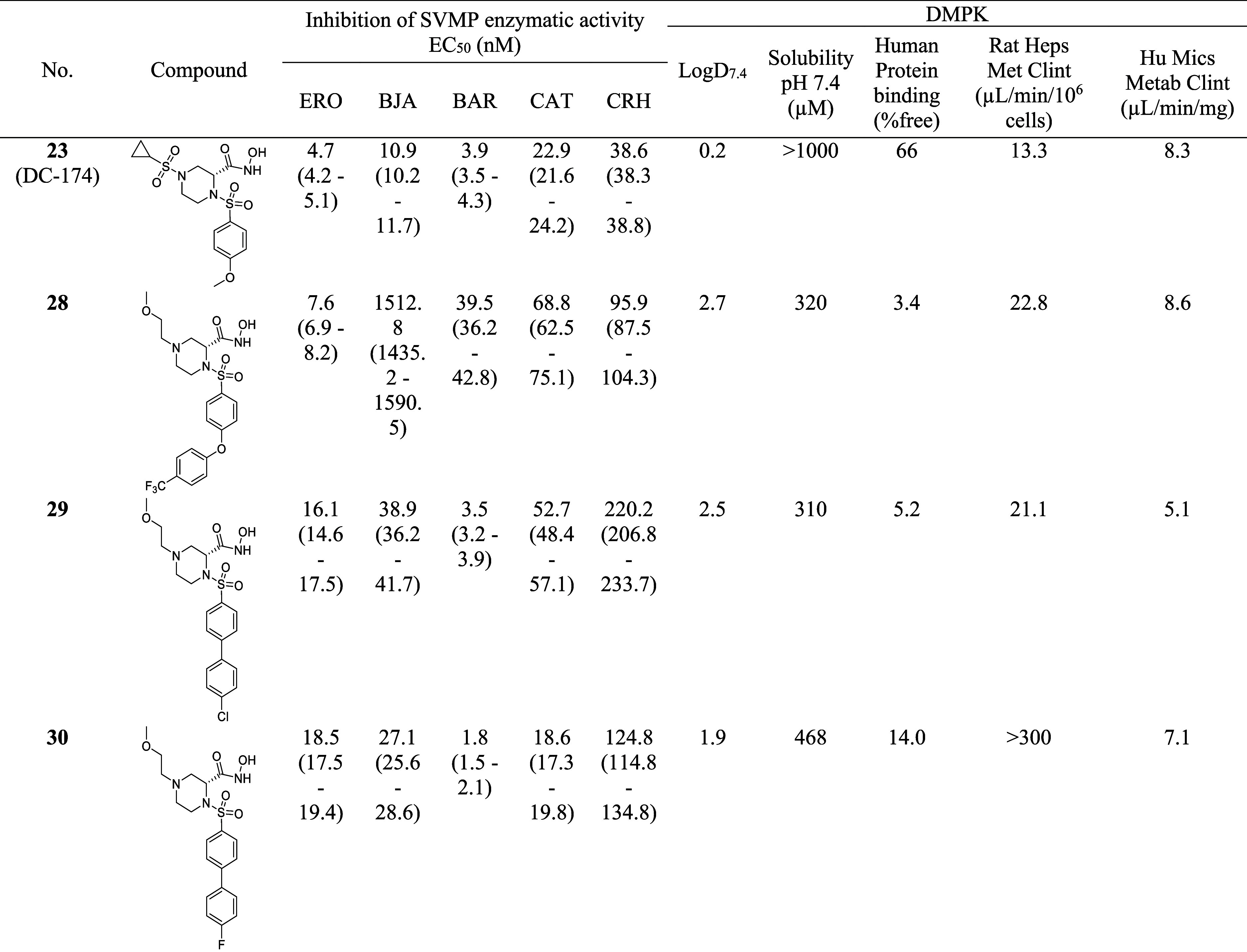
*In Vitro* Biological Evaluation of Compounds Described in Figure 2 ^a^

a ERO, *E. romani*; BJA, *B. jararaca*; BAR, *B. arietans*; CAT, *C. atrox*; CRH, *C. rhodostoma*. LogD_7.4_ – refers to the distribution coefficient between organic and aqueous solvent at physiological pH of 7.4. Rat Heps Met Clint – refers to the rate of *in vitro* hepatic intrinsic clearance in rat hepatocytes. Hu Mics Metab Clint – refers to the rate of *in vitro* hepatic intrinsic clearance in human microsomes. EC_50_ data represents means of *n* ≥ 3 repeats, bracketed values represent the range across repeats.

**Figure 2:**
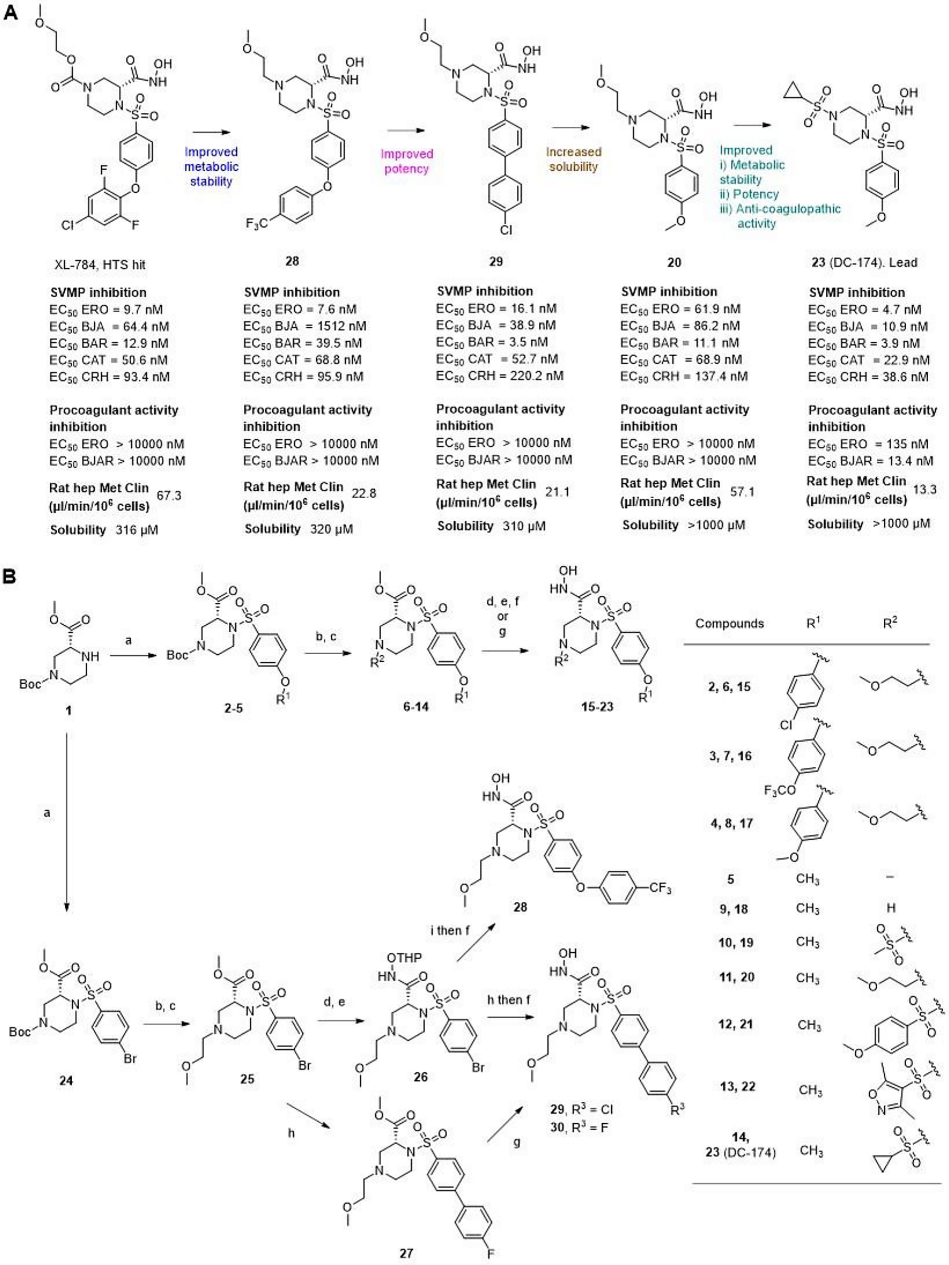
Hit-to-lead optimization toward DC-174 (23). (**A**) The structural progression from XL-784 to DC-174 (**23**). ERO, *E. romani*; BJA, *B. jararaca*; BAR, *Bitis arietans*; CAT, *Crotalus atrox*; CRH, *Calloselasma rhodostoma*. (**B**) Chemical synthetic schemes for compounds **15**-**23** and **28**-**30**. Reaction condition: a) sulfonyl chlorides, NEt_3_, DMAP, 1,4-dioxane, rt, 16 h, 67–95%; b) TFA, DCM, rt, 16 h; c) Br­(CH_2_)_2_OCH_3_, K_2_CO_3_, DMF, 60 °C, 16 h, 32–71% or sulfonyl chlorides, NEt_3_, DMAP, 1,4-dioxane, rt, 16 h, 70–93%; d) NaOH, MeOH, rt, 1 h; e) EDC, HOBt, DMF, NMM, THPO-NH_2_, rt, 16 h, 51–95%; f) 4 N HCl, 1,4-dioxane, rt, 1 h, 52–91%; g) NH_2_OH, KOCN, 1,4-dioxane, rt, 16 h, 47–94%; h) K_2_CO_3_, Pd­(PPh_3_)_4_, 1,4-dioxane, H_2_O, 80 °C, 79%; i) 4-(trifluoromethyl)­phenol, K_3_PO_4_, Pd­(OAc)_2_, di-tBuXPhos, toluene, 100 °C, 16 h, 15%.

The biphenyl scaffold was accessed using analogous chemistry: sulfonamide formation, *N*boc-deprotection, and *N*-alkylation to obtain aryl-bromide intermediate **25**. Due to limited commercial availability of trifluoromethyl sulfonyl chloride, hydroxamic acid **28** was synthesized via amide coupling, biphenyl ether formation, and THP deprotection using 4 N HCl. Additionally, para-halogenated biphenyl analogues **29** and **30** were prepared through Suzuki coupling reactions.

The para-substitution on the aromatic ring was initially accessed and hydroxamic acid **28** was able to retain similar potency to parent XL-784, while showing improvement in metabolic stability (rat hepatocyte clearance of 22.8 μL/min/10^6^ cells). Further SAR investigation of the ether linkage between the biphenyl motif showed that both para-halogenated **29** and **30** bolstered the overall SVMP potency profile, despite poor metabolic stability observed for the para-fluorinated **30**. The truncation of the biphenyl system demonstrated better aqueous solubility (>1000 μM) and significantly lower protein binding (82% free) for compound **20**. Next, the methoxy ethyl side chain was replaced with a sulfonamide group which revealed effective inhibition of procoagulant venom activity by compounds **19** and **21–23**. This distinct secondary activity assay measured inhibition of a key parameter of clinical envenoming, coagulopathy,^33^ which is often driven by SVMPs.^11^ All sulfonamide *N*-1 analogues displayed measurable inhibition of procoagulant venom activity (Table 2), unlike the parent molecule XL-784 (**1f**), monoaryl **20**, and biaryl analogues **29** and **30**, which were all inactive in this assay. The thiomorpholine-based biaryl prinomastat also demonstrated inhibition of procoagulant activity, suggesting inactivity of **20**, **29** and **30** could be due to the *N*-1 substituted methoxyethyl group. This dependence on the *N*-1 substituent was highlighted by the observation that EC_50s_ increased with steric bulk of the *N*-1 substituent from a minimum at **23** (cyclopropyl), increasing with **22** (dimethyl-isoxazole) and **21** (methoxyphenyl). Overall, the compact cyclopropyl sulfonamide **23** (DC-174) emerged as the most desirable analogue in the series with improved broad-spectrum potent inhibition of SVMP activity (4.7–38.6 nM EC_50s_), metabolic stability (rat hepatocyctes 13.3 μL/min/10^6^ cells) and inhibition of procoagulant venom activity (13.4 and 135.0 nM EC_50s_ in *B. jararaca* and *E. romani*, respectively).

**Table 2:** *In Vitro* Coagulation Assay of Selected compounds^a^

	Coagulation assay EC_50_ values (nM)	
Selected Inhibitors	*E. romani*	*B. jararaca*
**1d** (Prinomastat)	557.9 (490.7–625.1)	971.9 (892.2–1051.6)
**1f** (XL-784)	>10000	>10000
**19**	495.6 (447.4–543.8)	509.3 (421.7–596.8)
**20**	>10000	>10000
**21**	4586.6 (3786.6–5386.5)	3919.4 (3324.1–4514.6)
**22**	4852.0 (4220.3–5483.7)	1094.9 (926.0–1263.8)
**23** (DC-174)	135.0 (114.4–155.7)	13.4 (10.2–16.6)
**29**	>10000	>10000
**30**	>10000	>10000

a EC_50_ data represents means of *n* ≥ 3 repeats with range represented in brackets.

To explore whether **23** (DC174) inhibits the breadth of structurally distinct SVMP isoforms, we purified the P–I, P–II and P–III SVMPs from *E. romani* venom () and assessed their capacity to cleave the coagulation factors prothrombin and fibrinogen, before determining whether DC-174 (**23**) could prevent this coagulotoxic activity. All three SVMP classes degraded fibrinogen and prothrombin to varying extents (Figure 3A), except for the P–II on prothrombin (Figure S3). Comparisons of **23** against parent molecule XL-784 and prinomastat showed that all compounds protected against the degradation of fibrinogen, though XL-784 was inferior against P–II SVMP activity (Figure 3A, see lower molecular weight degradation bands). XL-784 was also unable to protect against prothrombin degradation by both P–I and P–III SVMPs, while **23** and prinomastat gave almost full protection, though **23** was superior based on visualization of fewer degradation products (Figure 3B). These data could explain the differences in potency in the earlier coagulation assay, since prothrombin activation is a critical driver of procoagulant venom activity.^11^

**Figure 3:**
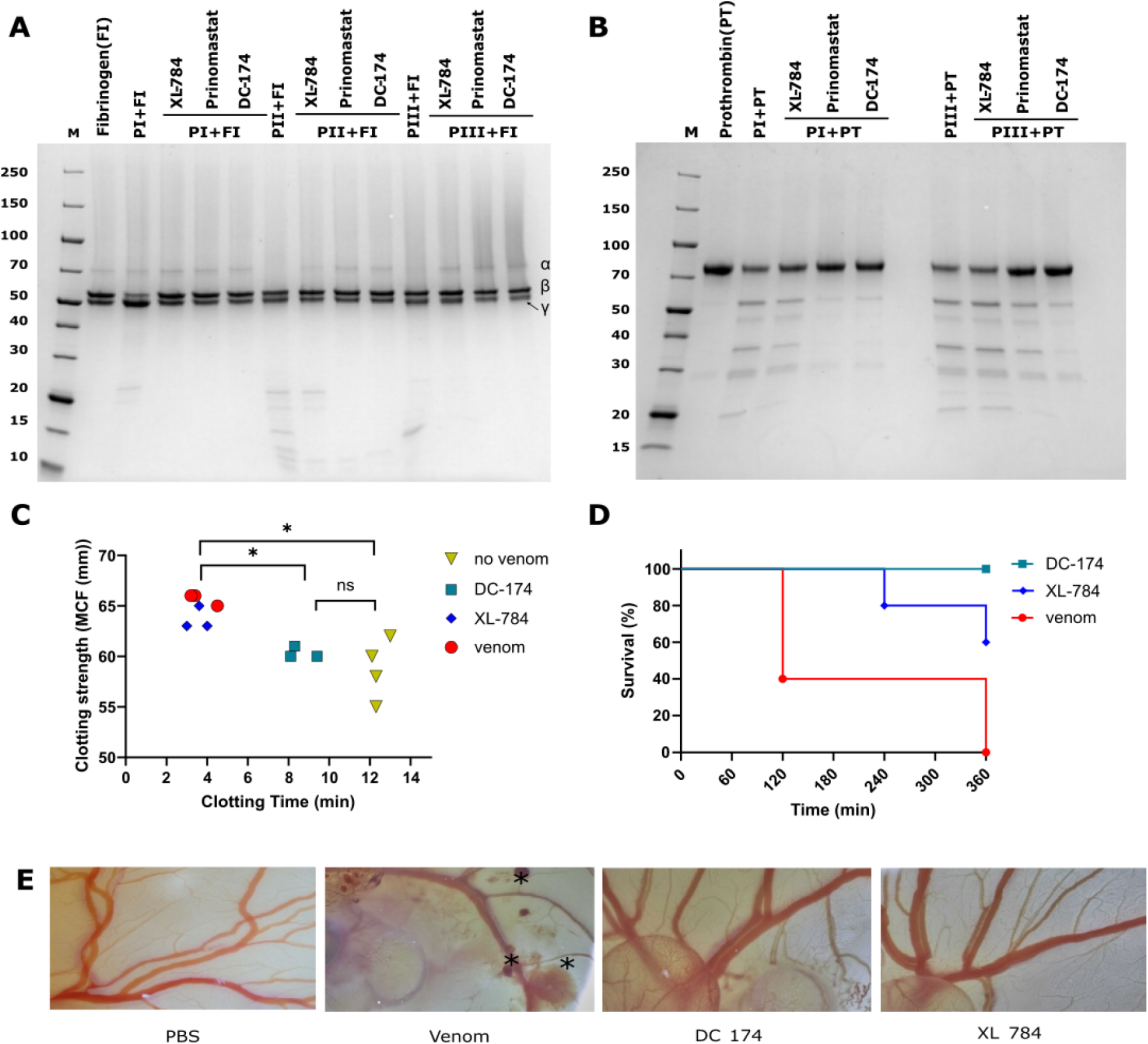
(DC-174) reduces SVMP isoform-mediated cleavage of clotting factors and venom-induced coagulopathy and protects against lethality and hemorrhage in a chicken embryo model of envenoming. Inhibition of the degradation of (**A**) fibrinogen and (**B**) prothrombin by XL-784, prinomastat, and **23**. The three different structural isoforms of *E. romani* SVMPs (P–I, P–II, P–III) were incubated for 30 min in the presence of each drug after which fibrinogen or prothrombin were added, the samples incubated for 1 h, before visualization of protein profiles via Coomassie staining of SDS-PAGE gel separations. The α, β and γ subunits of fibrinogen and indicated on the right side of the gel. (**C**) Thromboelastographic measurements of clotting time and MCF resulting from human whole blood treated with *E. romani* venom (60 ng) coadministered with vehicle (DMSO – red circles), DC-174 (**23**) (teal squares) and XL-784 (blue diamonds) compared with untreated controls (yellow triangles, *n* = 4). Treatments were dosed at 5 μM (*n* = 3). An ordinary one-way ANOVA was performed for all groups with significance indicated by asterisks (*, *p* < 0.05; F­(3,9) = 9.653, no venom vs XL-784, *p* = 0.029, venom vs DC-174 **(23)**, *p* = 0.027). (**D**) Survival curves of chicken embryos topically dosed with 10 μg of *E. romani* venom or venom immediately followed by 1 μg of either DC-174 (**23**) or XL-784 (*n* = 5). Embryos were observed at 1, 2, 4, and 6 h postexposure for the presence of an observable heartbeat. (**E**) Representative images of the dosed embryos from (d) at 1 h postvenom exposure. Images show *E. romani* venom only controls with extensive hemorrhage (asterisks), while those treated with DC-174 (**23**) or XL-784 (**1f**) displayed no evidence of hemorrhage at the same 1-h time point. A PBS-dosed (healthy) negative control is shown for comparative purposes.

To further explore the improved venom inhibition of **23** over its parent molecule XL-784 we used advanced assays measuring coagulation (thromboelastography^34^) and hemorrhage (*in vivo* chicken embryo model^35^), respectively. *E. romani* venom (60 ng) induced rapid clot formation in blood from healthy human donors (clotting time of 3.7 min [SD 0.7] vs 12.4 min [SD 0.4], nonvenom control) and induced a hypercoagulable state evidenced by increased maximum clot firmness (MCF) (65.7 mm [SD 0.6] vs 58.8 mm [SD 3.0]) (Figure 3C). While XL-784 had no effect on clotting time and only a modest reduction of venom-induced MCF (28.9% reduction), DC-174 (**23**) prolonged the mean clotting time (venom, 3.7 min [SD 0.7] vs venom and DC-174 (**23**), 8.6 min [SD 0.7]) and returned the MCF to the normal range (mean of 60.3 [SD 0.6] vs 58.8 [SD 3.0] nonvenom control, compared to 65.7 mm [SD 0.6] venom only control) (Figure 3C). To assess protection against venom-induced hemorrhage in an *in vivo* vascularized system, groups (*n* = 5) of six-day-old chicken embryos were topically dosed onto the vitelline vein with *E. romani* venom (10 μg/egg), followed by drug (1 μg/egg). Embryos dosed with venom died within 6 h (Figure 3D) and showed clear signs of hemorrhage as early as 1 h postdosing (Figure 3E). While XL-784 only provided partial protection against envenoming, with three (60% embryos surviving until the 6 h experimental end point, treatment with DC-174 (**23**) resulted in complete survival (Figure 3D) and also prevented venom-induced hemorrhage (Figure 3E).

We next progressed evaluation of DC-174 (**23**) into a modified version of the WHO-recommended, *in vivo* neutralization of venom-induced lethality murine model of snakebite envenoming.^14,15^ In these rescue experiments, groups of mice (*n* = 5) received a previously established lethal challenge dose of *E. romani* venom (90 μg^14^) intraperitoneally, followed by a 20 mg/kg oral dose of DC-174 (**23**) in standard suspension vehicle (SSV). A venom control group received SSV only instead of drug. All mice in the venom-only group succumbed to the lethal effects of envenoming within 161 min (111–161 min, mean 138 min), defined as implementation of euthanasia when humane end points associated with severe systemic envenoming were observed. **23** significantly prolonged survival times (*p* = 0.01), with three mice succumbing to the lethal effects of the venom much later in the experimental time frame (272, 370, and 372 min), while the remaining two mice survived until the 8-h experimental end point (Figure 4A).

**Figure 4:**
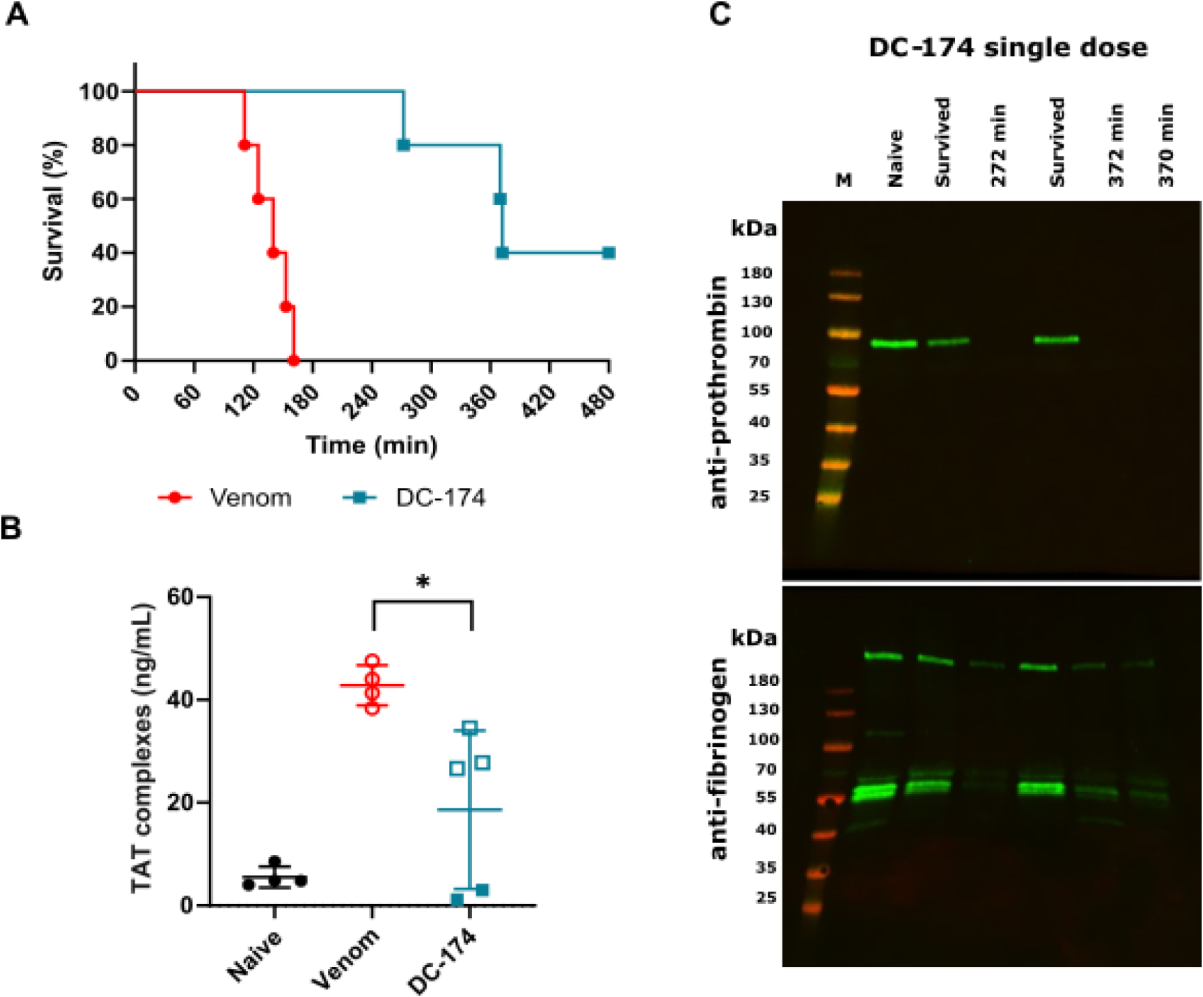
Oral DC-174 (**23**) prolongs survival in a murine preclinical model of severe envenoming. (**A**) Kaplan–Meier survival graph for mice (*n* = 5) dosed with *E. romani* venom (90 μg intraperitoneally) followed by immediate oral dosing with DC-174 (**23**) (20 mg/kg, teal squares) or SSV drug vehicle control (red circles). (**B**) ELISA quantification of TAT complexes from murine plasma samples from (A). Data from naïve nondosed mice (*n* = 4) are shown for comparison, and open and closed squares are used to discriminate between animals dosed with DC-174 (**23**) that died or survived, respectively. Error bars represent means ± SD. An ordinary one-way ANOVA was performed for all groups with significance indicated by an asterisk (F­(2,10) = 14.25, *p* = 0.012). (**C**) Antifibrinogen and antiprothrombin Western blots on murine plasma samples from (A) (*n* = 5), compared with samples from a naïve control mouse. Where animals succumbed to venom effects, they are annotated with time to death. M – molecular weight marker.

Blood samples were collected posteuthanasia for all experimental animals (at 8 h for survivors) and plasma isolated for biomarker analysis. We quantified thrombin-antithrombin (TAT) complexes, which are a marker of thrombin generation (i.e., proxy for coagulopathy) that has been shown to correlate with severity of envenoming.^14^ Resulting TAT levels were highly elevated in venom-only controls (mean 42.8 ng/mL vs 5.5 ng/mL in nonenvenomed mice). Mice receiving oral **23** displayed significant reductions in TAT levels (*p* = 0.01), with those that died during the experiment exhibiting modest reductions compared with the venom-only controls (mean 29.6 ng/mL vs 42.8 ng/mL, respectively), while the two survivors had TAT levels comparable to those of nonenvenomed mice (mean 2.1 ng/mL vs 5.5 ng/mL, respectively), suggesting protection against coagulopathic venom effects. Visualization of murine plasma prothrombin and fibrinogen via Western blotting showed a similar pattern. Venom only control mice had plasma depleted of prothrombin and almost completely degraded fibrinogen (Figure S4). Mice dosed orally with **23** that succumbed to envenoming showed similar profiles to the venom-only controls, with only traces of fibrinogen visible, but those that survived displayed intact prothrombin and fibrinogen profiles, comparable with those of naïve mice (Figure 4C).

While oral dosing of **23** significantly prolonged murine survival, the observed protection against severe envenoming was modest (257 min increase in mean survival times), which prompted us to evaluate the pharmacokinetic (PK) properties of the drug *in vivo*. PK analysis was evaluated over 24 h postoral administration (20 mg/kg) of **23** in mice (*n* = 9). Resulting data showed **23** had a rapid time to maximal drug concentration (*T*_max_, 0.5 h), but only a moderate half-life (*T*_1/2_, 1.41 h) and exposure (*C*_max_, 311 ng/mL) (Figure 5A). The moderate drug exposure could be the result of the low logD_7.4_ of DC-174 (**23**) (logD_7.4_ of 0.2), which can limit drug permeability and absorption.^36,37^ These data may explain the drop off in efficacy observed 4 h after oral dosing, since drug levels are predicted to be 26.1 ng/mL at this time point, an amount that equates to only 8.4% of the *C*_max_.

**Figure 5:**
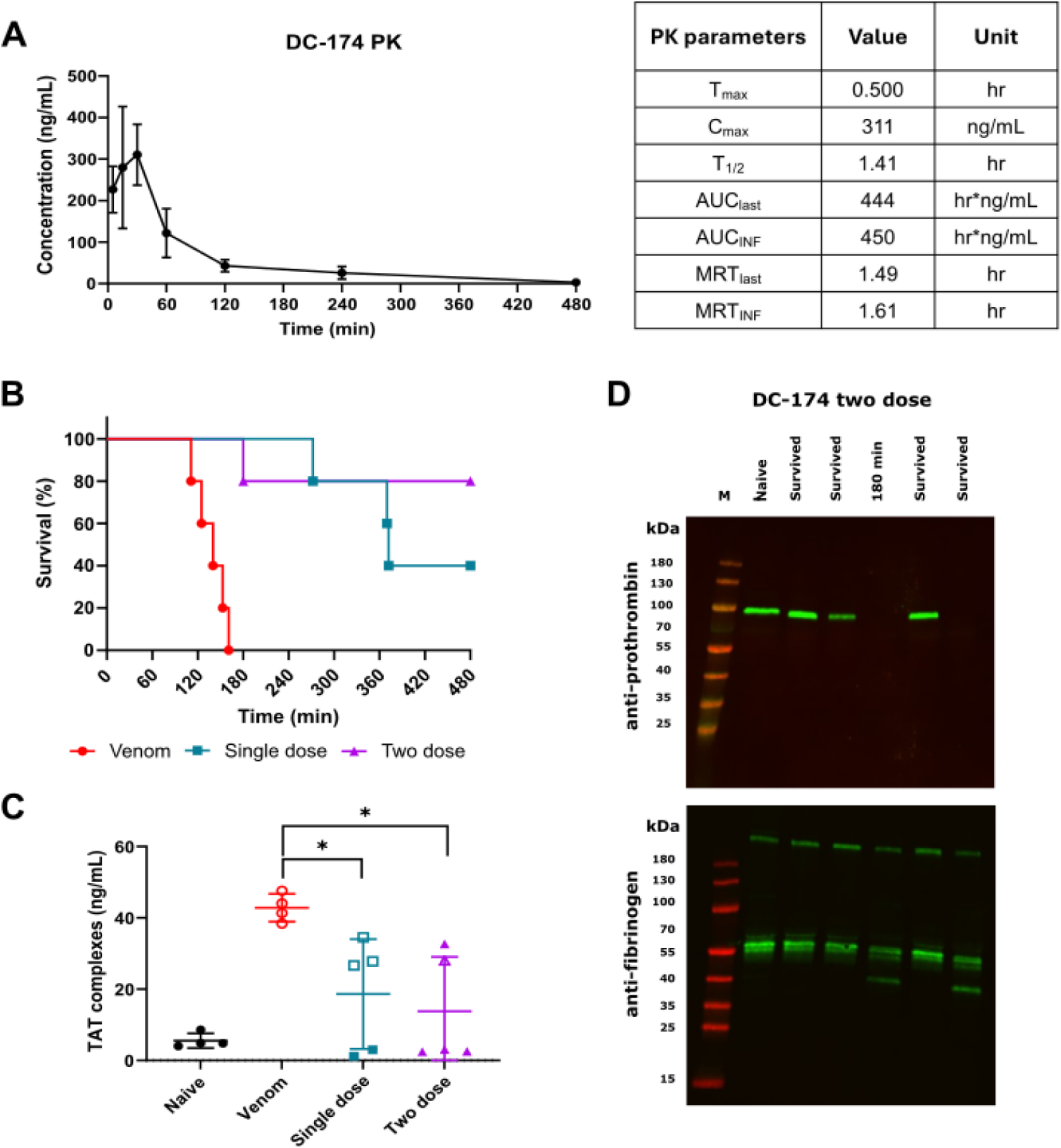
A two dose oral dosing regimen of 23 (DC-174) outperforms single oral dosing in a murine preclinical model of severe envenoming. (**A**) PK profiles of mice (*n* = 3) dosed orally with 20 mg/kg **23**. (**B**) Kaplan–Meier survival graph for mice (*n* = 5) dosed with *E. romani* venom (90 μg intraperitoneally) followed by oral dosing of **23** (20 mg/kg) immediately (single dose regimen) or immediately followed by a second 20 mg/kg dose 1.5 h later (two dose regimen). The venom vehicle controls received venom and a single dose of SSV drug vehicle control immediately thereafter. (**C**) ELISA quantification of TAT complexes from murine plasma samples from (B). Data from naïve nondosed mice (*n* = 4) are shown for comparison, and open and closed squares and triangles are used to discriminate between animals dosed with **23** that died or survived, respectively. Error bars represent means ± SD. An ordinary one-way ANOVA was performed for all groups with significance indicated by asterisks (*, F­(3,14) = 7.517, *p* < 0.05; venom vs single dose, *p* = 0.037, venom vs two dose, *p* = 0.011). (**D**) Antifibrinogen and antiprothrombin Western blots on mouse plasma samples collected from the repeat dosing of **23** (*n* = 5) vs a naïve control. Where animals succumbed to venom effects, they are annotated with time to lethality. M – molecular weight marker.

To test this hypothesis, we repeated the *in vivo* murine efficacy study using a multiple oral dosing regimen, with mice (*n* = 5) receiving the same 20 mg/kg oral dose of **23** immediately after venom challenge, followed by a second oral 20 mg/kg dose 1.5 h later. This multiple dosing regimen conferred an increase in efficacy, with only one experimental animal succumbing to the lethal effects of the venom (at 180 min), while the remaining four animals survived the duration of the 8 h experiment (Figure 5B). Mean survival times increased from 138 min (venom only control) to 420 min (two dose oral regimen). Analysis of resulting plasma samples revealed a further decrease in TAT levels when **23** was dosed twice (mean 13.7 ng/mL), lower than those observed with a single dose (mean 18.6 ng/mL) (Figure 5C). The number of mice with intact plasma prothrombin and fibrinogen also increased following repeat dosing (Figure 5D). Prothrombin was detectable in three of the four surviving mice, while fibrinogen was detectable in all multiply dosed animals, and with noticeably reduced levels of degradation compared with those dosed once (Figure 4C and Figure 5D). Collectively, this data suggests that the half-life of **23** may limit its efficacy, and we show that repeated oral administration provides a route to circumvent this limitation, resulting in increased efficacy against snakebite envenoming *in vivo*.

A major challenge in developing small molecule treatments for snakebite is achieving broad-spectrum activity across different toxin isoforms and venoms. Our *in vitro* results show that compound DC-174 (**23**) meets these criteria. To understand its binding, and that of the parent compound XL-784 (**1f**), molecular docking was performed using X-ray crystal structures of various SVMPs, including P–I and P–III classes (Figure 6 and Figure S5). No P–II SVMP structures were available for this analysis.

**Figure 6:**
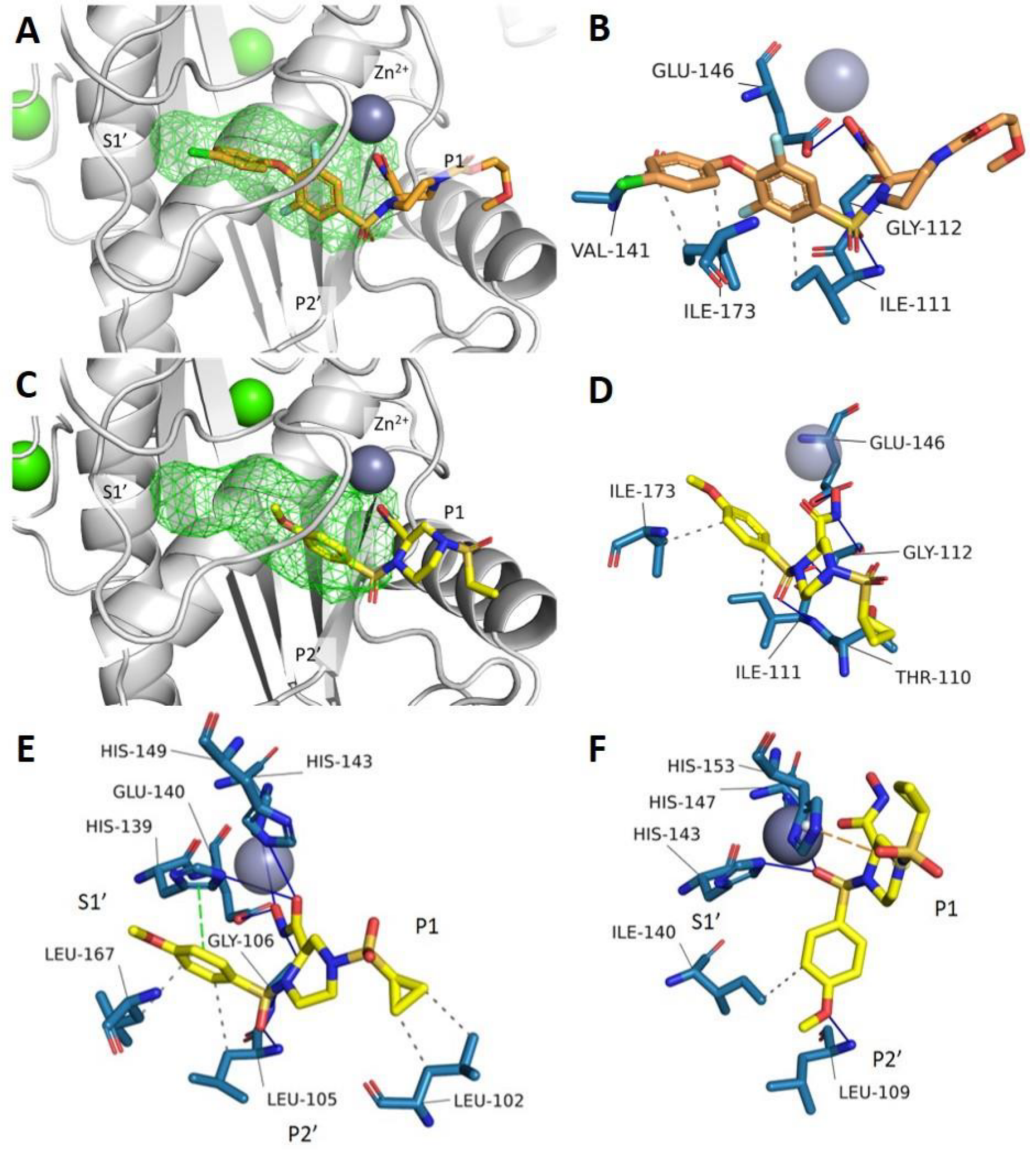
Molecular modeling predicted binding poses and interactions between SVMPs with XL-784 (**1f**) and DC-174 (**23**). (**A** and **B**) Docked poses of XL-784 (orange sticks), (**C** and **D**) **23** (yellow sticks) in the P–III SVMP bothropasin (PDB: 3DSL). (**E**) Docked poses of DC-174 (**23**) in RVV-X (PDB: 2E3X). (**F**) Docked poses of DC-174 (**23**) in ecarin (PDB: 9CLP) SVMPs. (A) and (C) Structure of the docked inhibitors in the SVMP binding site (gray cartoon), with the S1′ pocket highlighted in green wireframe. Zn^2+^ and Ca^2+^ ions are shown in deep blue and green, respectively. (B), (D), (E) and (F) Analysis of noncovalent interactions formed by inhibitors in the docked structures. Binding residues are shown in blue, hydrophobic interactions are shown as gray dashed lines, π-cation interactions are shown as orange dashed lines, π-stacking interaction are shown as green dashed lines, and hydrogen bonding interactions are shown as blue solid lines.

We explored structure–activity relationships (SAR) assuming XL-784 (**1f**) binds similarly to prinomastat (**1d**). Molecular docking of **1f** in the P–III SVMP bothropasin supported this (Figure 6A and B), showing a binding pose where its hydroxamic acid coordinates with the catalytic Zn^2+^ and its hydrophobic biaryl ether fits into the S1′ pocket,^38^ consistent with prinomastat (**1d**) binding to MMP13. The binding pocket primarily involves hydrophobic interactions with aliphatic residues Ile111, Val414, and Ile173. Hydrogen bonds form between the sulfone oxygen and hydroxamic acid with nearby backbone NH, carbonyl groups, and a glutamate in the Zn^2+^ binding domain. In the P–I SVMP atrolysin C, similar interactions occur, along with halogen bonding to backbone carbonyls and π-stacking with His142 in the Zn^2+^ domain (Figure S5A and B). In the P–I SVMP BaP1, although the core piperazine H-bonding is conserved, the S1′ pocket is too small for the biaryl ether of XL-784, which instead extends into the P2′ pocket (Figure S5C and D). This behavior is also seen in the P–III SVMP ecarin, where a stabilizing salt bridge forms (Figure S5e). In the P–III SVMP RVV-X, XL-784 adopts a reversed pose, avoiding the S1′ pocket (Figure S5f). These findings across SVMP isoforms suggest that the size of the S1′ substituent strongly influences the binding mode and inhibitory spectrum of the molecule.

**23** was docked into SVMP crystal structures to compare its interaction profile with XL-784 (**1f**). In bothropasin (Figure 6C and D), the hydrogen bonding between the piperazine-hydroxamic acid and key residues, including glutamate in the Zn^2+^ binding domain, was preserved with additional interactions with sulfone and backbone NH groups observed. The methoxyphenyl ring of DC-174 (**23**) fits well into the S1 pocket, forming hydrophobic interactions with Ile173 and Ile111, while all key hydrogen bonds from XL-784 (**1f**) were conserved with additional ones formed. Docking studies of **23** in BaP1 (Figure S6A and B), atrolysin C (Figure S6C and D), and RVV-X (Figure 6E) show that its truncated monoaryl ring is well tolerated across multiple SVMP isoforms, unlike XL-784 (**1f**), which was hindered by the small S1′ pocket in BaP1 (Figure S5C). In ecarin, **23** adopted an outward pose along the P1 and P2′ pockets, stabilized by π-cation and hydrogen bond interactions with Leu109 (Figure 6F). However, its piperazine-hydroxamic acid hydrogen bonding was not conserved, instead forming a bond via the sulfone group. Interaction analysis indicates that key hydrogen bonding motifs are conserved in both P–I SVMP isoforms (Figure S6B and D). Unique π-stacking interactions with His142 was only observed in BaP1 (Figure S6B). Unlike XL-784 (**1f**), the cyclopropyl sulfonamide moiety in **23** forms hydrophobic interactions with residues in atrolysin C, BaP1, and RVV-X, and an additional hydrogen bond with His152 in atrolysin C.

Further docking and molecular dynamic studies (see Supporting Information for molecular modeling) collectively provide a structural basis for the broader interspecies and cross-isoform inhibitory activity of **23** over its parent compound XL-784 (**1f**). The predicted binding mode of **23** across structurally diverse SVMPs also indicates its venom-neutralizing effect stems from direct SVMP inhibition.

## Discussion and Conclusion

Herein, we have described the first experimental design, synthesis and evaluation of SVMP inhibitory properties, resulting in an orally bioavailable precandidate for development of a snakebite drug. We showed that a rationally designed small molecule inhibitor can effectively neutralize diverse SVMP toxins from a wide range of medically important snake venoms. Unlike the parent molecule XL-784 (**1f**) and other previously described SVMP inhibitors (i.e., prinomastat (**1d**)),^24,39,40^ inhibitor DC-174 (**23**) neutralizes all three of the structurally diverse SVMP subclasses (i.e., P–I, P–II and P–III), likely as the result of its truncated monoaryl ring system. Further, inhibitor **23** is capable of fully or near fully preventing the generation of thrombin by inhibiting prothrombin proteolysis by *E. romani* P–I and P–III SVMPs, respectively (Figure 3). The ability of certain SVMPs to generate thrombin and activate the clotting cascade forms the basis of venom-induced consumption coagulopathy (VICC), whereby venom metalloproteinases cleave and deplete the internal pool of clotting factors leading to incoagulable blood. As such, **23** can prevent the initiation of one of the main pathways responsible for causing coagulopathy, which in turn exacerbates hemorrhage, in snakebite patients. These findings are reinforced from the *in vivo* models applied, with **23** preventing hemorrhage in an *in vivo* egg embryo model and significantly prolonging survival and reducing coagulopathy when administered orally following *E. romani* venom challenge in a murine model of envenoming (Figures 3–5).

The murine PK data obtained for **23** shows that it only takes 30 min for drug levels to reach their peak following oral dosing, but that the half-life is rather short at 1.41 h (Figure 5A). The clearance of **23** may underlie the intermediate level of protection observed following single oral administration in mice (i.e., significant increase in survival times, but only 40% survival at 8 h). In agreement with this hypothesis, we showed that repeated administration of **23**, with a second dose 1.5 h after the first, improves drug efficacy, resulting in 80% survival at the end of the 8 h experiment. Plasma biomarkers of coagulation also demonstrated that repeat dosing of **23** increased efficacy, with TAT, prothrombin and fibrinogen levels superior to those observed with single dosing.

To contextualize these findings, the metal chelator DMPS – an SVMP inhibiting molecule that is currently in clinical trials for snakebite indication^41^ – is the only other SVMP inhibiting drug with demonstrated efficacy via oral dosing.^14^ **23** outperforms DMPS both in terms of *in vitro* potency (ERO EC_50_s, 9.7 nM vs 1.65 μM)^14^ and *in vivo* efficacy (oral dosing murine survival of 80% vs 40% at 8 h with 20 mg/kg twice dosing vs 30 mg/kg single dosing, respectively^14^). Future efficacy comparisons of **23** with other MMP inhibitors, such as marimastat (**1c**) and prinomastat (**1d**), will be valuable, particularly since these molecules have yet to be evaluated against snakebite envenoming *in vivo* via the oral route. Irrespectively, these results collectively underscore the increased efficacy of **23** relative to its parent molecule, XL-784 (**1f**) and other lead SVMP-inhibiting drugs, and emphasizes the importance of applying rational, structure-informed chemical modification to improve PK and PD parameters of next-generation treatments for snakebite envenoming.

This work establishes a novel drug discovery pipeline for SVMP inhibitors that provide reassurance of “on-target” mechanism of action. Further, the highly efficient chemical synthetic strategy for production of **23** only requires three chemical reaction steps and utilizes affordable, commercially available materials for synthesis, highlighting the potential future cost-effectiveness of oral drugs as alternatives to the costly polyclonal antibody-based antivenom therapies currently available. Additional development work on **23** will next be required to establish robust preclinical toxicology and off-target specificity profiles (e.g., various MMPs), and expand PK profiling to predict a suitable human dosing regimen. In parallel, our future medicinal chemistry efforts will focus on maximizing the metabolic stability characteristics of this scaffold to provide a broad-spectrum efficacious molecule compatible with a single dose regimen, as well as evaluating the potential of **23** as a component of combination therapies with other oral drugs that target different venom toxins,^15,16^ such as the PLA_2_ inhibitor varespladib.^19^

DC-174 (**23**) broadens the current narrow portfolio of only two SVMP inhibiting drugs progressing into clinical development for snakebite, thus helping to avoid attrition as the field moves toward an optimized oral treatment. This novel SVMP inhibitor represents one of several unique molecules currently being developed through our discovery platform with the long-term goal of accelerating treatment options available for the tropical communities most afflicted by life-threatening snakebite. Future efficacy comparisons of **23** with other MMP inhibitors, such as marimastat (**1c**) and prinomastat (**1d**), will be valuable, particularly since these molecules have yet to be evaluated against snakebite envenoming *in vivo* via the oral route.

## Experimental Section

### Chemistry

Chemicals were purchased from Sigma-Aldrich, Fluorochem, or Alfa Aesar and used without further purification. Unless otherwise stated, anhydrous solvents and dry atmospheric conditions were used in all reactions. Unless otherwise stated, all reactions were carried out at room temperature (rt) under atmospheric pressure. Thin layer chromatography (TLC) was carried out on silica gel plates 60 F_254_ and visualized under UV light at 254 nm. Potassium permanganate dip was used to visualize non-UV active compounds. Flash column chromatography separations using 40–63 μm silica were performed by the gradient elution method, and the elution solvent system is given in each instance. Reverse-phase high-performance liquid chromatography (RP-HPLC) was carried out on an Agilent 1260 Infinity system, using a ZORBAX SB-C18 (9.4 mm × 250 mm, 5 μm) column at a rate of 4 mL/min for semipreparative RP-HPLC and a ZORBAX Eclipse Plus C18 (4.6 mm × 100 mm, 3.5 μm) column at a rate of 1 mL/min for analytical RP-HPLC. The following mobile phases were used A, H_2_O (+0.1% v/v formic acid), and B, ACN (+0.1% v/v formic acid). The standard analytical method for analytical RP-HPLC (for which retention times of compounds are given) was 5% B for 1 min and a linear gradient from 5% to 95% A from 1 to 12 min (followed by a 3 min hold at 95% B and then a 1 min linear gradient from 95% to 5% B and a 1 min re-equilibration at 5% B). All final compounds were >95% RP-HPLC analytically pure as assessed by peak integration at 254 nm (Supporting Information). A typical semipreparative HPLC method was carried out as described: 5% B for 1 min and a linear gradient from 5% to 95% B from 1 to 12 min (followed by a 2 min hold at 95% B and then a 1 min linear gradient from 95% to 5%. High-resolution electrospray ionization mass spectra (HRMS-ESI) were recorded on an Agilent QTOF 6540 spectrometer. Proton (^1^H) and carbon (^13^C) nuclear magnetic resonance (NMR) spectroscopy were carried out using either a Bruker AMX 400 (^1^H, 400 MHz; ^13^C, 101 MHz) or 500 MHz (^1^H, 500 MHz; ^13^C, 126 MHz) spectrometer. Chemical shifts are listed on the δ scale in parts per million, referenced to CDCl_3_ (^1^H NMR δ7.26; ^13^C NMR δ77.16) or MeOD-*d*_4_ (^1^H NMR δ3.31; ^13^C NMR δ49.03) with residual solvent as the internal standard and coupling constants (*J*) recorded in hertz. Note that not all magnetically nonequivalent carbons were observed in the ^13^C NMR spectrum for all compounds. Signal multiplicities are assigned as follows: s, singlet; d, doublet; t, triplet; q, quartet; dd, doublet of doublets; br, broad; m, multiplet. Single-crystal X-ray diffraction was mounted on a MiTGen tip via Parbol oil and data were collected on a Bruker D8 Venture Photon III diffractometer. The crystal was kept at 200.0 K during data collection. Using Olex2, the structure was solved with the XT structure solution program using Intrinsic Phasing and refined with the XL refinement package using Least Squares minimization. Crystallographic data for DC-174 (**23**) have been deposited in CCDC database (Deposition number: 2451930).

### General Procedure for the Synthesis of Sulfonamides **2**–**5**, **10**, **12**–**14** and **24**

1-(*tert*-Butyl) 3-methyl (*R*)-piperazine-1,3-dicarboxylate (SM) and 4-dimethylaminopyridine (DMAP) was charged into a 100 mL round-bottom flask, sealed with a septum, evacuated and backfilled with nitrogen (2 times). 1,4-Dioxane and triethylamine is added and the reaction mixture was stirred. Appropriate sulfonyl chloride (dissolved in 1,4-dioxane) was added dropwise and the reaction mixture was stirred for 16 h. Water was added and the aqueous solution was extracted with ethyl acetate (2 × 50 mL). The pooled organic solution was dried with MgSO_4_, filtered and concentrated *in vacuo*. The crude product was purified by column chromatography.

#### 1-(*tert*-Butyl) 3-Methyl (R)-4-((4-(4-chlorophenoxy)­phenyl)­sulfonyl)­piperazine-1,3-dicarboxylate (**2**)

SM (1.00 g, 4.09 mmol), DMAP (50 mg, 0.41 mmol), 1,4-dioxane (30 mL), triethylamine (1.71 mL, 12.28 mmol), 4-(4-chlorophenoxy)­benzenesulfonyl chloride (1.24 g, 4.09 mmol, in 5 mL of 1,4-dioxane) following column elution from 0% to 20% ethyl acetate in hexane to give **2** (1.73 g, 3.38 mmol, 83% as colorless liquid. ^1^H NMR (400 MHz, CDCl_3_) δ ^1^H NMR (400 MHz, CDCl_3_) δ 7.74–7.69 (d, *J* = 8.8 Hz, 2H), 7.39–7.33 (d, *J* = 8.8 Hz, 2H), 7.07–6.94 (m, 4H), 4.62–4.56 (m, 2H), 4.56–4.51 (m, 1H), 4.22–3.92 (m, 1H), 3.69–3.59 (m, 1H), 3.56 (s, 3H), 3.50–3.30 (m, 1H), 3.23–3.04 (m, 1H), 3.03–2.77 (m, 1H), 1.40 (s, 9H).

#### 1-(*tert*-Butyl) 3-Methyl (R)-4-((4-(4-(trifluoromethoxy)­phenoxy)­phenyl)­sulfonyl)­piperazine-1,3-dicarboxylate (**3**)

SM (128 mg, 0.52 mmol), DMAP (7 mg, 0.057 mmol), 1,4-dioxane (5 mL), triethylamine (0.15 mL, 1.57 mmol), 4-(4-(trifluoromethoxy)­phenoxy)­benzenesulfonyl chloride (185 mg, 0.52 mmol, in 2 mL of 1,4-dioxane) following column elution from 0% to 20% ethyl acetate in hexane to give **3** (220 mg, 0.39 mmol, 75% as pale-yellow liquid. ^1^H NMR (400 MHz, CDCl_3_) δ 7.78–7.70 (d, *J* = 8.8 Hz, 2H), 7.24 (d, *J* = 9.2 Hz, 2H), 7.07 (d, *J* = 9.2 Hz, 2H), 7.04 (d, *J* = 8.8 Hz, 4H), 4.62–4.57 (m, 1H), 4.57–4.44 (m, 1H), 4.20–3.99 (m, 1H), 3.68–3.60 (m, 1H), 3.56 (s, 3H), 3.49–3.30 (m, 1H), 3.23–3.05 (m, 1H), 3.02–2.78 (m, 1H), 1.40 (s, 9H).

#### 1-(*tert*-Butyl) 3-Methyl (R)-4-((4-(4-methoxyphenoxy)­phenyl)­sulfonyl)­piperazine-1,3-dicarboxylate (**4**)

SM (189 mg, 1.23 mmol), DMAP (14 mg, 0.11 mmol), 1,4-dioxane (10 mL), triethylamine (0.49 mL, 3.69 mmol), 4-(4-methoxyphenoxy)­benzenesulfonyl chloride (350 mg, 4.32 mmol, in 5 mL of 1,4-dioxane) following column elution from 0% to 20% ethyl acetate in hexane to give **4** (400 mg, 0.79 mmol, 64% as pale-yellow liquid. ^1^H NMR (400 MHz, CDCl_3_) δ 7.68 (d, *J* = 8.8 Hz, 2H), 7.04–6.88 (m, 6H), 4.61–4.55 (m, 1H), 4.55–4.43 (m, 1H), 4.20–3.93 (m, 1H), 3.83 (s, 3H), 3.66–3.58 (m, 1H), 3.55 (s, 3H), 3.49–3.30 (m, 1H), 3.21–3.04 (m, 1H), 3.01–2.78 (m, 1H), 1.40 (s, 9H).

#### 1-(*tert*-Butyl) 3-Methyl (R)-4-((4-methoxyphenyl)­sulfonyl)­piperazine-1,3-dicarboxylate (**5**)

SM (1.00 g, 4.09 mmol), DMAP (50 mg, 0.41 mmol), 1,4-dioxane (30 mL), triethylamine (1.71 mL, 12.28 mmol), 4-methoxybenzenesulfonyl chloride (1.25 g, 4.09 mmol, in 10 mL of 1,4-dioxane) following column elution from 0% to 20% ethyl acetate in hexane to give **5** (1.61 g, 3.88 mmol, 95% as colorless liquid. ^1^H NMR (400 MHz, CDCl_3_) δ 7.69 (d, *J* = 9.2 Hz, 2H), 6.95 (d, *J* = 9.2 Hz, 4H), 4.60–4.53 (m, 1H), 4.54–4.39 (m, 1H), 4.20–3.93 (m, 1H), 3.86 (s, 3H), 3.67–3.57 (m, 1H), 3.53 (s, 3H), 3.47–3.29 (m, 1H), 3.20–3.02 (m, 1H), 2.99–2.75 (m, 1H), 1.39 (s, 9H).

#### Methyl (R)-1-((4-Methoxyphenyl)­sulfonyl)-4-(methylsulfonyl)­piperazine-2-carboxylate (**10**)

Boc-protected **5** (467 mg, 1.12 mmol) was first deprotected with TFA (10 equiv) in DCM (15 mL). The crude reaction mixture was carefully neutralized with saturated sodium bicarbonate solution to give crude methyl *(R)*-1-((4-methoxyphenyl)­sulfonyl)­piperazine-2-carboxylate. Following that, methyl *(R)*-1-((4-methoxyphenyl)­sulfonyl)­piperazine-2-carboxylate (356 mg, 1.12 mmol), DMAP (14 mg, 0.11 mmol), 1,4-dioxane (10 mL), triethylamine (0.47 mL, 3.39 mmol), methanesulfonyl chloride (92 μL, 1.19 mmol) following column elution from 0% to 50% ethyl acetate in hexane to give **10** (412 mg, 1.05 mmol, 93% as colorless liquid. ^1^H NMR (400 MHz, CDCl_3_) δ 7.73 (d, *J* = 9.2 Hz, 2H), 6.97 (d, *J* = 9.2 Hz, 2H), 4.85–4.75 (m, 1H), 4.25–4.17 (m, 1H), 3.87 (s, 3H), 3.81–3.67 (m, 2H), 3.62 (s, 3H), 3.40 (m, 1H), 3.02 (m, 1H), 2.84 (m, 1H), 2.78 (s, 3H).

#### Methyl (R)-1,4-Bis­((4-methoxyphenyl)­sulfonyl)­piperazine-2-carboxylate (**12**)

Boc-protected **5** (421 mg, 1.02 mmol) was first deprotected with TFA (10 equiv) in DCM (15 mL). The crude reaction mixture was carefully neutralized with saturated sodium bicarbonate solution to give crude methyl *(R)*-1-((4-methoxyphenyl)­sulfonyl)­piperazine-2-carboxylate. Following that, methyl *(R)*-1-((4-methoxyphenyl)­sulfonyl)­piperazine-2-carboxylate (318 mg, 1.02 mmol), DMAP (12 mg, 0.098 mmol), 1,4-dioxane (10 mL), triethylamine (0.42 mL, 3.03 mmol), 4-methoxybenzenesulfonyl chloride (310 mg, 1.02 mmol) following column elution from 0% to 50% ethyl acetate in hexane to give **12** (384 mg, 0.79 mmol, 78% as colorless liquid. ^1^H NMR (400 MHz, CDCl_3_) δ 7.67 (d, *J* = 9.2 Hz, 2H), 7.64 (d, *J* = 9.2 Hz, 2H), 6.99 (d, *J* = 8.8 Hz, 2H), 6.93 (d, *J* = 8.8 Hz, 2H), 4.75–4.70 (m, 1H), 4.19–4.13 (m, 1H), 3.88 (s, 3H), 3.85 (s, 3H), 3.74–3.62 (m, 2H), 3.62 (s, 3H), 3.47–3.37 (m, 1H), 2.53 (dd, *J* = 11.7, 4.0 Hz, 1H), 2.32–2.30 (m, 1H).

#### Methyl (R)-4-((3,5-Dimethylisoxazol-4-yl)­sulfonyl)-1-((4-methoxyphenyl)­sulfonyl)­piperazine-2-carboxylate (**13**)

Boc-protected **5** (696 mg, 1.68 mmol) was first deprotected with TFA (10 equiv) in DCM (10 mL). The crude reaction mixture was carefully neutralized with saturated sodium bicarbonate solution to give crude methyl (R)-1-((4-methoxyphenyl)­sulfonyl)­piperazine-2-carboxylate. Following that, methyl (R)-1-((4-methoxyphenyl)­sulfonyl)­piperazine-2-carboxylate (528 mg, 2.16 mmol), DMAP (26 mg, 0.21 mmol), 1,4-dioxane (15 mL), triethylamine (0.90 mL, 6.48 mmol), 3,5-dimethylisoxazole-4-sulfonyl chloride (423 mg, 2.16 mmol) following column elution from 0% to 50% ethyl acetate in hexane to give **13** (561 mg, 1.18 mmol, 55% as yellow liquid. ^1^H NMR (400 MHz, CDCl_3_) δ 7.70 (d, *J* = 9.2 Hz, 2H), 6.96 (d, *J* = 9.2 Hz, 2H), 4.81–4.74 (m, 1H), 4.10–4.03 (m, 1H), 3.87 (s, 3H), 3.82–3.74 (m, 1H), 3.72–3.64 (m, 1H), 3.57 (s, 3H), 3.40 (td, *J* = 12.0, 3.2 Hz, 1H), 2.94–2.85 (m, 1H), 2.72 (td, *J* = 12.0, 3.2 Hz, 1H), 2.62 (s, 3H), 2.32 (s, 3H).

#### Methyl (R)-4-(Cyclopropylsulfonyl)-1-((4-methoxyphenyl)­sulfonyl)­piperazine-2-carboxylate (**14**)

Boc-protected **5** (466 mg, 1.12 mmol) was first deprotected with TFA (10 equiv) in DCM (10 mL). The crude reaction mixture was carefully neutralized with saturated sodium bicarbonate solution to give crude methyl (R)-1-((4-methoxyphenyl)­sulfonyl)­piperazine-2-carboxylate. Following that, methyl (R)-1-((4-methoxyphenyl)­sulfonyl)­piperazine-2-carboxylate (354 mg, 1.45 mmol), DMAP (18 mg, 0.15 mmol), 1,4-dioxane (10 mL), triethylamine (0.61 mL, 4.35 mmol), cyclopropanesulfonyl chloride (148 μL, 1.45 mmol) following column elution from 0% to 50% ethyl acetate in hexane to give **14** (365 mg, 0.87 mmol, 60% as colorless liquid. ^1^H NMR ^1^H NMR (400 MHz, CDCl_3_) δ 7.73 (d, *J* = 9.2 Hz, 2H), 6.97 (d, *J* = 9.2 Hz, 2H), 4.80–4.75 (m, 1H), 4.26–4.17 (m, 1H), 3.87 (s, 3H), 3.81–3.66 (m, 2H), 3.62 (s, 3H), 3.41 (td, *J* = 12.4, 3.4 Hz, 1H), 3.17–3.10 (m, 1H), 2.96 (td, *J* = 12.4, 3.4 Hz, 1H), 2.26–2.15 (m, 1H), 1.19–1.10 (m, 2H), 1.05–0.94 (m, 2H).

#### 1-(*tert*-Butyl) 3-Methyl (R)-4-((4-bromophenyl)­sulfonyl)­piperazine-1,3-dicarboxylate (**24**)

SM (1.00 g, 4.09 mmol), DMAP (50 mg, 0.41 mmol), 1,4-dioxane (30 mL), triethylamine (1.71 mL, 12.28 mmol), 4-bromobenzenesulfonyl chloride (1.05 g, 4.09 mmol, in 5 mL of 1,4-dioxane) following column elution from 0% to 20% ethyl acetate in hexane to give **24** (1.34 g, 2.89 mmol, 70% as pale-yellow liquid. ^1^H NMR (400 MHz, CDCl_3_) δ 7.67–7.58 (m, 4H), 4.62–4.57 (m, 2H), 4.57–4.45 (m, 1H), 4.24–3.93 (m, 1H), 3.70–3.59 (m, 1H), 3.54 (s, 3H), 3.47–3.28 (m, 1H), 3.21–3.04 (m, 1H), 3.02–2.75 (m, 1H), 1.40 (s, 9H).

### General Procedure for the Alkylation of *N*-Boc Piperazine **6**–**8**, **11** and **25**

In a 50 mL round-bottom flask, N-Boc piperazine was treated with trifluoroacetic acid (TFA) in DCM and the reaction mixture was stirred for 16 h. The reaction mixture was concentrated and redissolved in DCM. The organic solution was washed carefully with saturated solution of sodium bicarbonate, dried with MgSO_4_, filtered and concentrated *in vacuo*. To the resulting crude mass, potassium carbonate is added and the round-bottom flask was sealed with a septum. Anhydrous DMF was added and the reaction mixture was stirred for 30 min. 2-Bromoethyl methyl ether was added dropwise and the reaction mixture was stirred for 30 min before being heated to 60 °C for 16 h. The reaction mixture was concentrated *in vacuo* and water was added. The aqueous solution was extracted with ethyl acetate (3 times). The pooled organic solution was dried with MgSO_4_, filtered and concentrated *in vacuo*. The crude product was purified by column chromatography.

#### Methyl (R)-1-((4-(4-chlorophenoxy)­phenyl)­sulfonyl)-4-(2-methoxyethyl)­piperazine-2-carboxylate (**6**)

**2** (0.79 g, 1.54 mmol), TFA (1.18 mL, 15.40 mmol), DCM (10 mL) to give crude mass of 634 mg. Potassium carbonate (426 mg, 3.08 mmol), DMF (6 mL), 2-bromoethyl methyl ether (290 μL, 3.08 mmol) following column elution from 10% to 60% ethyl acetate in hexane to give **6** (512 mg, 1.09 mmol, 71% as colorless liquid. ^1^H NMR (400 MHz, CDCl_3_) δ 7.75 (d, *J* = 8.8 Hz, 2H), 7.35 (d, *J* = 8.8 Hz, 2H), 7.06–6.96 (m, 4H), 4.64–4.59 (m, 1H), 3.61 (s, 3H), 3.48–3.33 (m, 4H), 3.30 (s, 3H), 2.81–2.74 (m, 1H), 2.63–2.45 (m, 2H), 2.42–2.32 (m, 1H), 2.26 (td, *J* = 11.5, 3.6 Hz, 1H).

#### Methyl (R)-1-((4-bromophenyl)­sulfonyl)-4-(2-methoxyethyl)­piperazine-2-carboxylate (**25**)

**3** (750 mg, 1.85 mmol), TFA (1.42 mL, 18.50 mmol), DCM (10 mL) to give crude mass of 606 mg. Potassium carbonate (461 mg, 3.34 mmol), DMF (6 mL), 2-bromoethyl methyl ether (314 μL, 2.27 mmol) following column elution from 40% to 100% ethyl acetate in hexane to give 2**5** (393 mg, 0.93 mmol, 50% as colorless liquid. ^1^H NMR (400 MHz, CDCl_3_) δ 7.68–7.60 (m, 4H), 4.64–4.60 (m, 1H), 3.65–3.59 (m, 1H), 3.58 (s, 3H), 3.46–3.31 (m, 4H), 3.29 (s, 3H), 2.83–2.74 (m, 1H), 2.63–2.46 (m, 2H), 2.37 (dd, *J* = 11.5, 3.8 Hz, 1H), 2.25 (td, *J* = 10.9, 5.4 Hz, 1H).

#### Methyl (R)-4-(2-methoxyethyl)-1-((4-(4-(trifluoromethoxy)­phenoxy)­phenyl)­sulfonyl)­piperazine-2-carboxylate (**7**)

**3** (579 mg, 1.03 mmol), TFA (0.79 mL, 10.30 mmol), DCM (10 mL) to give crude mass of 429 mg. Potassium carbonate (386 mg, 2.79 mmol), DMF (5 mL), 2-bromoethyl methyl ether (131 μL, 1.39 mmol) following column elution from 40% to 100% ethyl acetate in hexane to give **7** (270 mg, 0.52 mmol, 50% as colorless liquid. ^1^H NMR (400 MHz, CDCl_3_) δ 7.77 (d, *J* = 8.8 Hz, 2H), 7.26–7.20 (m, 2H), 7.12–7.00 (m, 4H), 4.67–4.58 (m, 1H), 3.71–3.53 (m, 4H), 3.51–3.33 (m, 4H), 3.31 (s, 3H), 2.85–2.71 (m, 1H), 2.66–2.46 (m, 2H), 2.45–2.34 (m, 1H), 2.33–2.20 (m, 1H).

#### Methyl (R)-4-(2-methoxyethyl)-1-((4-(4-methoxyphenoxy)­phenyl)­sulfonyl)­piperazine-2-carboxylate (**8**)

**4** (400 mg, 0.79 mmol), TFA (0.60 mL, 7.90 mmol), DCM (10 mL) to give crude mass of 235 mg. Potassium carbonate (120 mg, 0.87 mmol), DMF (5 mL), 2-bromoethyl methyl ether (82 μL, 0.87 mmol) following column elution from 40% to 100% ethyl acetate in hexane to give **8** (155 mg, 0.33 mmol, 42% as pale-yellow liquid. ^1^H NMR (400 MHz, CDCl_3_) δ 7.73 (d, *J* = 8.8 Hz, 2H), 7.06–6.91 (m, 6H), 4.65–4.61 (m, 1H), 3.85 (s, 3H), 3.66–3.56 (m, 1H), 3.63 (s, 3H), 3.50–3.35 (m, 4H), 3.32 (s, 3H), 2.82–2.75 (m, 1H), 2.65–2.48 (m, 2H), 2.40 (dd, *J* = 11.5, 3.9 Hz, 1H), 2.26 (td, *J* = 11.2, 5.6 Hz, 1H).

#### Methyl (R)-4-(2-methoxyethyl)-1-((4-methoxyphenyl)­sulfonyl)­piperazine-2-carboxylate (**11**)

**5** (675 mg, 2.56 mmol), TFA (1.96 mL, 25.60 mmol), DCM (10 mL) to give crude mass of 456 mg. Potassium carbonate (300 mg, 2.17 mmol), DMF (10 mL), 2-bromoethyl methyl ether (177 μL, 1.88 mmol) following column elution from 40% to 100% ethyl acetate in hexane to give **11** (329 mg, 0.88 mmol, 34% as colorless liquid. ^1^H NMR (400 MHz, CDCl_3_) δ 7.72 (d, *J* = 8.8 Hz, 2H), 6.95 (d, *J* = 8.8 Hz, 2H), 4.63–4.58 (m, 1H), 3.86 (s, 3H), 3.59 (s, 3H), 3.58–3.52 (m, 1H), 3.48–3.38 (m, 2H), 3.38–3.31 (m, 2H), 3.29 (s, 3H), 2.79–2.71 (m, 1H), 2.61–2.44 (m, 2H), 2.36 (dd, *J* = 11.5, 3.9 Hz, 1H), 2.22 (td, *J* = 11.5, 3.6 Hz, 1H).

#### Methyl (R)-1-((4-methoxyphenyl)­sulfonyl)­piperazine-2-carboxylate (**9**)

In a 50 mL round-bottom flask, N-Boc protected **5** (1.60 g, 3.86 mmol) was treated with trifluoroacetic acid (5 mL) in DCM (10 mL) and the reaction mixture was stirred for 16 h. The reaction mixture was concentrated and redissolved in DCM. The organic solution was washed carefully with saturated solution of sodium bicarbonate, dried with MgSO_4_, filtered and concentrated *in-vacuo* to give **9** (1.08 g, 3.44 mmol, 89% as white crystals. ^1^H NMR (400 MHz, CDCl_3_) δ 7.74–7.68 (d, *J* = 8.8 Hz, 2H), 7.00–6.91 (d, *J* = 8.8 Hz, 2H), 4.55 (m, 1H), 3.86 (s, 3H), 3.62–3.52 (m, 4H), 3.42–3.35 (m, 1H), 3.33–3.24 (m, 1H), 3.02–2.91 (m, 2H), 2.77 (td, *J* = 12.2, 3.7 Hz, 1H).

#### (R)-1-((4-(4-Chlorophenoxy)­phenyl)­sulfonyl)-*N*-hydroxy-4-(2-methoxyethyl)­piperazine-2-carboxamide (**15**)

In a 25 mL round-bottom flask, methyl ester **4** (36 mg, 0.076 mmol) was dissolved in methanol (1 mL). Aqueous 2 M sodium hydroxide (0.38 mL, 0.76 mmol) solution was added and the reaction was stirred for 1 h. The reaction mixture was concentrated *in vacuo* and water was added. The aqueous was washed with ethyl acetate (2 times). The aqueous solution was acidified to pH 3–4 with aqueous HCl (2 M) and the reaction mixture was extracted with ethyl acetate (3 times). The pooled organic solution was dried with MgSO_4_, filtered and concentrated *in vacuo* to give crude carboxylic acid (28 mg, 0.061 mmol, 80% and was used without further purification. A 25 mL round-bottom flask was charge with the carboxylic acid (28 mg, 0.061) starting material, hydroxybenzotriazole (25.0 mg, 0.185 mmol), 1-ethyl-3-(3-(dimethylamino)­propyl)­carbodiimide (0.185 mmol, 35 mg) and THP-protected hydroxylamine (22 mg, 0.185 mmol). Anhydrous DMF (5 mL) and *N*-methylmorpholine (20 μL, 0.185 mmol) were sequentially added and the reaction was stirred overnight. Water was added and the aqueous solution was extracted with ethyl acetate (3 times). The pooled organic solution was dried with MgSO_4_, filtered and concentrated *in vacuo*. The crude product was purified by column chromatography following column elution from 50% to 100% ethyl acetate in hexane to give OTHP-amide coupled product (30 mg, 0.055 mmol, 89% as colorless liquid. In a 25 mL round-bottom flask, the resulting OTHP-protected hydroxyl amine (30 mg, 0.054 mmol) was dissolved in 1,4-dioxane (5 mL). Four N HCl (1 mL in dioxane) was added and the reaction was stirred for 1 h. The reaction mixture was neutralized to pH 7 with saturated solution of sodium bicarbonate. The solution was extracted with ethyl acetate (3 times). The pooled organic solution was dried with MgSO_4_, filtered and concentrated *in vacuo*. The crude product was purified by column chromatography following column elution of 10% methanol in DCM to give **15** (151 mg, 0.32 mmol, 52% as white crystals. ^1^H NMR (400 MHz, CDCl_3_) δ 7.85 (d, *J* = 9.2 Hz, 2H), 7.35 (d, J = 9.2 Hz, 2H), 7.05–6.96 (m, 4H), 4.69–4.64 (m, 1H), 3.73–3.64 (m, 1H), 3.55–3.41 (m, 2H), 3.37 (s, 3H), 3.22–3.15 (m, 1H), 3.07 (td, *J* = 12.8, 3.1 Hz, 1H), 2.82–2.75 (m, 1H), 2.72–2.63 (m, 1H), 2.54–2.46 (m, 1H), 2.41–2.31 (m, 2H). ^13^C NMR (126 MHz, CDCl_3_) δ 165.43, 161.25, 154.00, 133.62, 130.30, 130.26, 130.13, 121.63, 117.68, 69.21, 59.15, 56.52, 55.49, 52.85, 52.40, 42.62. HMRS (ESI) calculated for C_20_H_24_ClN_3_O_6_S^+^ [M + H]^+^ *m*/*z* 470.1147, found 470.1148. HPLC purity: 95.5% (RT: 8.51 min).

### General Procedure for the Conversion of Methyl Ester to Hydroxylamine **16**–**23** and **30**

Methyl ester was first dissolved 1,4-dioxane. Potassium cyanate (KOCN) followed by aqueous 50% aqueous hydroxylamine was added and the reaction was stirred for 16 h. The reaction mixture was then concentrated, water and brine were added. The aqueous solution was extracted with ethyl acetate (3 times). The pooled organic solution was dried with MgSO_4_, filtered and concentrated *in-vacuo*. The crude product was purified by column chromatography. Unless otherwise stated, the purified mass undergoes subsequent semipreparative HPLC purification to achieve product purity above 95%.

#### (R)-*N*-Hydroxy-4-(2-methoxyethyl)-1-((4-(4-(trifluoromethoxy)­phenoxy)­phenyl)­sulfonyl)­piperazine-2-carboxamide (**16**)

Methyl ester **7** (165 mg, 0.32 mmol), 1,4-dioxane (8 mL), KOCN (36 mg, 0.44 mmol), 50% aqueous hydroxylamine (8 mL) following column elution from 60% to 100% ethyl acetate in hexane to give **16** (85 mg, 0.16 mmol, 52% as a colorless liquid. ^1^H NMR (400 MHz, CDCl_3_) δ 7.87 (d, *J* = 8.9 Hz, 2H), 7.24 (d, *J* = 8.9 Hz, 2H), 7.07 (d, *J* = 9.2, 2H), 7.03 (d, *J* = 9.2 Hz, 2H), 4.69–4.65 (m, 1H), 3.74–3.65 (m, 1H), 3.57–3.41 (m, 2H), 3.36 (s, 3H), 3.30–3.22 (m, 1H), 3.18–3.12 (m, 1H), 2.86–2.80 (m, 1H), 2.73–2.67 (m, 1H), 2.59–2.52 (m, 1H), 2.41–2.34 (m, 2H). ^13^C NMR (101 MHz, CDCl_3_) δ 165.44, 161.16, 153.85, 145.79 (q, *J* = 1.9 Hz), 133.74 (m), 130.29, 123.04, 121.34, 120.36 (q, *J* = 256.84 Hz), 117.86, 68.89, 58.95, 56.40, 55.12, 52.59, 52.25. HRMS (ESI) calculated for C_21_H_25_F_3_N_3_O_7_S^+^ [M + H]^+^ *m*/*z* 520.1360, C_42_H_48_F_6_N_6_NaO_14_S_2_^+^ [2M+Na]^+^ *m*/*z* 1061.2466, found 520.1374 and 1061.2479. HPLC purity: 96.7% (RT: 7.73 min).

#### (R)-*N*-Hydroxy-4-(2-methoxyethyl)-1-((4-(4-methoxyphenoxy)­phenyl)­sulfonyl)­piperazine-2-carboxamide (**17**)

Methyl ester **8** (150 mg, 0.32 mmol), 1,4-dioxane (7 mL), KOCN (37 mg, 0.45 mmol), 50% aqueous hydroxylamine (7 mL) following column elution from 60% to 100% ethyl acetate in hexane to give **17** (112 mg, 0.24 mmol, 75% as a colorless liquid. ^1^H NMR (400 MHz, CDCl_3_) δ 7.79 (d, *J* = 8.8 Hz, 2H), 7.05–6.87 (m, 6H), 4.65–4.60 (m, 1H), 3.82 (s, 3H), 3.69–3.62 (m, 1H), 3.54–3.40 (m, 2H), 3.36 (s, 3H), 3.23–3.16 (m, 1H), 3.15–3.05 (m, 1H), 2.79–2.71 (m, 1H), 2.68–2.59 (m, 1H), 2.53–2.44 (m, 1H), 2.33–2.22 (m, 2H). ^13^C NMR (101 MHz, CDCl_3_) δ 165.72, 162.65, 156.97, 148.31, 132.57, 130.05, 121.86, 116.74, 115.29, 69.10, 58.94, 56.44, 55.79, 55.28, 52.49, 52.13, 42.44. HRMS (ESI) calculated for C_21_H_28_N_3_O_7_S^+^ [M + H]^+^ *m*/*z* 466.1642, found 466.1693. HPLC purity: 99.7% (RT: 9.44 min).

#### (R)-*N*-Hydroxy-1-((4-methoxyphenyl)­sulfonyl)­piperazine-2-carboxamide hydrochloride salt (**18**)

Methyl ester **9** (218 mg, 0.69 mmol), 1,4-dioxane (7 mL), KOCN (79 mg, 0.97 mmol), 50% aqueous hydroxylamine (7 mL). The reaction mixture was concentrated and redissolved in ethyl acetate (20 mL). The organic solution was washed with water (2 × 20 mL), dried with MgSO_4_, filtered and concentrated. The resulting crude was treated with 4 N HCl (5 mL in dioxane) and stirred for 15 min. The reaction mixture was concentrated and redissolved in methanol (1 mL). Dichloromethane was added dropwise and the precipitate formed was collected by filtration to give **18** (195 mg, 0.55 mmol, 80% as a white solid. ^1^H NMR (500 MHz, MeOD) δ 7.83 (d, *J* = 8.9 Hz, 2H), 7.12 (d, *J* = 8.9 Hz, 2H), 4.65–4.60 (m, 1H), 3.95–3.86 (m, 4H), 3.70–3.58 (m, 2H), 3.36–3.32 (m, 1H), 3.15–3.09 (m, 1H), 2.96 (td, *J* = 12.8, 4.1 Hz, 1H). ^13^C NMR (126 MHz, MeOD) δ 165.34, 130.91, 130.87, 115.84, 56.32, 50.44, 45.30, 43.69, 40.60. HRMS (ESI) calculated for C_12_H_18_N_3_O_5_S^+^ [M + H]^+^ *m*/*z* 316.0962, found 316.0968. HPLC purity: 98.7% (RT: 0.87 min).

#### (R)-*N*-Hydroxy-1-((4-methoxyphenyl)­sulfonyl)-4-(methylsulfonyl)­piperazine-2-carboxamide (**19**)

Methyl ester **10** (412 mg, 1.05 mmol), 1,4-dioxane (10 mL), KOCN (119 mg, 1.47 mmol), 50% aqueous hydroxylamine (10 mL) following column elution from 60% to 100% ethyl acetate in hexane to give **19** (194 mg, 0.49 mmol, 47% as a white solid. ^1^H NMR (500 MHz, CDCl_3_) δ 7.78 (d, *J* = 8.9 Hz, 2H), 7.02 (d, *J* = 8.9 Hz, 2H), 4.68–4.62 (m, 1H), 4.23–4.16 (m, 1H), 3.89 (s, 3H), 3.88–3.80 (m, 1H), 3.62–3.54 (m, 1H), 3.39–3.29 (m, 1H), 2.83 (s, 3H), 2.71–2.62 (m, 1H), 2.61–2.52 (m, 1H). ^13^C NMR (101 MHz, CDCl_3_) δ 165.57, 163.92, 130.27, 129.65, 115.10, 55.92, 54.31, 44.61, 43.50, 43.08, 37.60. HRMS (ESI) calculated for C_13_H_20_N_3_O_7_S_2_^+^ [M + H]^+^ *m*/*z* 394.0737 and C_13_H_19_N_3_NaOS_2_^+^ [M + Na]^+^ *m*/*z* 416.0557, found 394.0742 and 416.0558. HPLC purity: 99.6% (RT: 6.55 min).

#### (R)-*N*-Hydroxy-4-(2-methoxyethyl)-1-((4-methoxyphenyl)­sulfonyl)­piperazine-2-carboxamide (**20**)

Methyl ester **11** (153 mg, 0.41 mmol), 1,4-dioxane (7 mL), KOCN (46 mg, 0.57 mmol), 50% aqueous hydroxylamine (7 mL) following column elution from 60% to 100% ethyl acetate in hexane to give **20** (101 mg, 0.27 mmol, 66% as a colorless liquid. ^1^H NMR (400 MHz, CDCl_3_) δ 7.81 (d, *J* = 8.9 Hz, 2H), 6.96 (d, *J* = 8.9 Hz, 2H), 4.66–4.58 (m, 1H), 3.86 (s, 3H), 3.71–3.62 (m, 1H), 3.54–3.39 (m, 2H), 3.36 (s, 3H), 3.24–3.16 (m, 1H), 3.16–3.05 (m, 1H), 2.77–2.70 (m, 1H), 2.66–2.57 (m, 1H), 2.51–2.43 (m, 1H), 2.29–2.17 (m, 2H). (101 MHz, CDCl_3_) δ 165.77, 163.18, 131.19, 129.99, 114.27, 69.13, 58.94, 56.52, 55.73, 55.25, 52.39, 52.06, 42.46. HRMS (ESI) calculated for C_15_H_24_N_3_O_6_S^+^ [M + H]^+^ *m*/*z* 374.1380, found 374.1376. HPLC purity: 97.5% (RT: 4.98 min).

#### (R)-*N*-Hydroxy-1,4-bis­((4-methoxyphenyl)­sulfonyl)­piperazine-2-carboxamide (**21**)

Methyl ester **12** (380 mg, 0.78 mmol), 1,4-dioxane (19 mL), KOCN (46 mg, 0.57 mmol), 50% aqueous hydroxylamine (19 mL) following column elution from 60% to 100% ethyl acetate in hexane, and subsequently recrystallized using DCM to give **21** (277 mg, 0.57 mmol, 73% as a white solid. ^1^H NMR (400 MHz, DMSO) δ 10.77 (br s, 1H), 9.01 (br s, 1H), 7.64 (d, *J* = 8.8 Hz, 2H), 7.47 (d, *J* = 8.8 Hz, 2H), 7.10 (d, *J* = 9.0 Hz, 2H), 6.92 (d, *J* = 9.0 Hz, 2H), 4.46–4.44 (m, 1H), 3.88 (s, 3H), 3.84–3.71 (m, 5H), 3.57–3.46 (m, 1H), 3.32–3.25 (m, 1H), 1.85–1.78 (m, 1H), 1.71–1.61 (m, 1H). ^13^C NMR (101 MHz, DMSO) δ 164.16, 162.96, 162.67, 131.07, 129.70, 129.05, 124.87, 114.37, 55.67, 55.58, 53.00, 45.93, 43.81, 42.14. HRMS (ESI) calculated for C_19_H_24_N_3_O_8_S_2_^+^ [M + H]^+^ *m*/*z* 486.0999 and C_19_H_23_N_3_NaO_8_S_2_^+^ [M + Na]^+^ *m*/*z* 508.0819, found 486.1003 and 508.0817. HPLC purity: 96.1% (RT: 8.01 min).

#### (R)-4-((3,5-Dimethylisoxazol-4-yl)­sulfonyl)-*N*-hydroxy-1-((4-methoxyphenyl)­sulfonyl)­piperazine-2-carboxamide (**22**)

Methyl ester **13** (555 mg, 1.17 mmol), 1,4-dioxane (28 mL), KOCN (133 mg, 1.64 mmol), 50% aqueous hydroxylamine (28 mL) following column elution from 60% to 100% ethyl acetate in hexane to give **22** (356 mg, 0.75 mmol, 64% as a colorless liquid. ^1^H NMR (400 MHz, MeOD) δ 7.77 (d, *J* = 9.0 Hz, 2H), 7.03 (d, *J* = 9.0 Hz, 2H), 4.67–4.62 (m, 1H), 4.04–3.92 (m, 2H), 3.90 (s, 3H), 3.57–3.45 (m, 1H), 3.43–3.36 (m, 1H), 2.54 (s, 3H), 2.32–2.23 (m, 4H), 2.16–2.07 (m, 1H). (101 MHz, MeOD) δ 175.94, 166.74, 165.18, 159.17, 132.34, 130.75, 115.65, 113.14, 56.28, 55.11, 45.71, 44.29, 43.52, 12.95, 11.25. HRMS (ESI) calculated for C_17_H_23_N_4_O_8_S_2_^+^ [M + H]^+^ *m*/*z* 475.0952, found 475.0959. HPLC purity: 99.5% (RT: 7.82 min).

#### (R)-4-(Cyclopropylsulfonyl)-*N*-hydroxy-1-((4-methoxyphenyl)­sulfonyl)­piperazine-2-carboxamide (**23**) (DC-174)

Methyl ester **14** (360 mg, 0.86 mmol), 1,4-dioxane (18 mL), KOCN (98 mg, 1.21 mmol), 50% aqueous hydroxylamine (18 mL) following column elution from 60% to 100% ethyl acetate in hexane to give **23** (DC-174) (338 mg, 0.80 mmol, 94% as a colorless liquid. ^1^H NMR (500 MHz, MeOD) δ 7.82 (d, *J* = 8.9 Hz, 2H), 7.10 (d, *J* = 8.9 Hz, 2H), 4.57–4.54 (m, 1H), 4.06–4.01 (m, 1H), 3.88 (s, 3H), 3.87–3.83 (m, 1H), 3.62–3.48 (m, 2H), 2.86 (dd, *J* = 12.6, 4.3 Hz, 1H), 2.75–2.64 (td, *J* = 11.5, 3.0 Hz, 1H), 2.40–2.32 (m, 1H), 1.02–0.90 (m, 4H). ^13^C NMR (126 MHz, MeOD) δ 167.39, 165.04, 132.31, 130.76, 115.70, 56.27, 55.20, 47.67, 45.63, 43.97, 27.01, 4.93, 4.74. HRMS (ESI) calculated for C_15_H_22_N_3_O_7_S_2_^+^ [M + H]^+^ *m*/*z* 420.0894, found 420.0899. HPLC purity: 99.8% (RT: 7.16 min). Elemental Analysis calculated for C_15_H_21_N_3_O_7_S_2_ requires C, 42.96%, H, 4.99%, N, 9.74%, S, 15.14%. Found C, 42.99%, H, 5.03%, N, 9.70%, S, 15.19%. See for X-ray crystallography data (CCDC deposition number: 2451930).

#### (R)-1-((4′-Fluoro-[1,1′-biphenyl]-4-yl)­sulfonyl)-*N*-hydroxy-4-(2-methoxyethyl)­piperazine-2-carboxamide (**30**)

Methyl ester **27** (166 mg, 0.38 mmol), 1,4-dioxane (8 mL), KOCN (36 mg, 0.44 mmol), 50% aqueous hydroxylamine (8 mL) following column elution from 60% to 100% ethyl acetate in hexane to give **30** (130 mg, 0.30 mmol, 78% as a colorless liquid. ^1^H NMR (400 MHz, CDCl_3_) δ 7.95 (d, *J* = 8.4 Hz, 2H), 7.66 (d, *J* = 8.4 Hz, 2H), 7.61–7.54 (m, 2H), 7.20–7.11 (m, 2H), 4.74–4.70 (m, 1H), 3.77–3.70 (m, 1H), 3.56–3.41 (m, 2H), 3.37 (s, 3H), 3.24–3.18 (m, 1H), 3.16–3.06 (m, 1H), 2.83–2.75 (m, 1H), 2.72–2.62 (m, 1H), 2.54–2.45 (m, 1H), 2.41–2.30 (m, 2H). ^13^C NMR (101 MHz, CDCl_3_) δ 165.60, 163.17 (d, *J* = 249.5 Hz), 144.75, 138.29, 135.55 (d, *J* = 3.3 Hz), 129.19 (d, *J* = 9.1 Hz), 128.46, 127.49, 116.15 (d, *J* = 21.2 Hz), 69.02, 58.95, 56.39, 55.34, 52.60, 52.16, 42.52. HRMS (ESI) calculated for C_20_H_25_FN_3_O_5_S^+^ [M + H]^+^ *m*/*z* 438.1493, C_20_H_24_FN_3_NaO_5_S^+^ [M + Na]^+^ *m*/*z* 460.1313, C_40_H_48_F_2_N_6_NaO_10_S_2_^+^ [2M+Na]^+^ *m*/*z* 897.2734, found 438.1496, 460.1307, and 897.2734. HPLC purity: 95.9% (RT: 6.77 min).

#### (2R)-1-((4-Bromophenyl)­sulfonyl)-4-(2-methoxyethyl)-*N*-((tetrahydro-2H-pyran-2-yl)­oxy)­piperazine-2-carboxamide (**26**)

In a 25 mL round-bottom flask, methyl ester 2**5** (625 mg, 1.48 mmol) was dissolved in methanol (20 mL). Aqueous 2 M sodium hydroxide (8.50 mL, 17.0 mmol) solution was added and the reaction was stirred for 1 h. The reaction mixture was concentrated *in vacuo* and water was added. The aqueous was washed with ethyl acetate (2 times). The aqueous solution was acidified to pH 3–4 with aqueous 2 M HCl and the reaction mixture was extracted with ethyl acetate (3 times). The pooled organic solution was dried with MgSO_4_, filtered and concentrated *in-vacuo* to give a crude carboxylic acid (493 mg, 1.21 mmol, 82% as white solid. The crude carboxylic acid was used without further purification. A 25 mL round-bottom flask was charge with the resulting carboxylic acid (493 mg, 1.21 mmol), hydroxybenzotriazole (490 mg, 3.63 mmol), 1-Ethyl-3-(3-(dimethylamino)­propyl)­carbodiimide (696 mg, 3.63 mmol) and THP-protected hydroxylamine (425 mg, 3.63 mmol). Anhydrous DMF (20 mL) and *N*-methylmorpholine (400 μL, 3.63 mmol) were sequentially added and the reaction was stirred overnight. Water was added and the aqueous solution was extracted with ethyl acetate (3 times). The pooled organic solution was dried with MgSO_4_, filtered and concentrated *in vacuo*. The crude product was purified by column chromatography following column elution from 50% to 100% ethyl acetate in hexane to give **26** (1.01 g, 1.99 mmol, 51% as pale-yellow liquid. ^1^H NMR (400 MHz, CDCl_3_) δ 7.80–7.71 (m, 2H), 7.62 (d, *J* = 8.1 Hz, 2H), 4.90–4.85 (m, 1Ha), 4.61–4.55 (m, H), 4.33–4.26 (m, 1Hb), 3.91–3.65 (m, 2H), 3.52–3.42 (m, 2H), 3.32 (s, 3H), 3.26–3.10 (m, 2H), 2.91–2.78 (m, 1H), 2.70–2.48 (m, 2H), 2.44–2.28 (m, 2H), 1.84–1.46 (m, 7H).

#### Methyl (R)-1-((4′-fluoro-[1,1′-biphenyl]-4-yl)­sulfonyl)-4-(2-methoxyethyl)­piperazine-2-carboxylate (**27**)

A pressure tube was charged with an aryl-bromide **25**, 1,4-dioxane (4 mL), tetrakis­(triphenylphosphine)­palladium(0) (20.0 mg, 0.017 mmol), (4-fluorophenyl)­boronic acid (133 mg, 0.64 mmol) and aqueous 2 M K_2_CO_3_ (1.65 mL). The reaction mixture was heated at 80 °C for 16 h. Water was added and the mixture was extracted with ethyl acetate (3 times). The pooled organic solution was dried with MgSO_4_, filtered and concentrated *in vacuo*. The crude product was purified by column chromatography following column elution from 10% to 50% ethyl acetate in hexane to give **27** (173 mg, 0.40 mmol, 69% as a colorless liquid. ^1^H NMR (400 MHz, CDCl_3_) δ 7.84 (d, *J* = 8.8 Hz, 2H), 7.65 (d, *J* = 8.8 Hz, 2H), 7.60–7.54 (m, 2H), 7.20–7.12 (m, 2H), 4.68–4.63 (m, 1H), 3.69–3.62 (m, 1H), 3.57 (s, 3H), 3.46–3.35 (m, 4H), 3.29 (s, 3H), 2.82–2.75 (m, 1H), 2.62–2.46 (m, 2H), 2.43–2.36 (m, 1H), 2.27 (td, *J* = 11.5, 3.6 Hz, 1H).

#### (R)-*N*-Hydroxy-4-(2-methoxyethyl)-1-((4-(4-(trifluoromethyl)­phenoxy)­phenyl)­sulfonyl)­piperazine-2-carboxamide (**28**)

A seal tube was charged with aryl bromide **26** (148 mg, 0.29 mmol), 4-(trifluoromethyl)­phenol (57 mg, 0.35 mmol), di-*t*-BuXphos (13 mg, 0.030 mmol), palladium acetate (7 mg, 0.031 mmol), potassium phosphate (124 mg, 0.58 mmol) in anhydrous toluene (5 mL) and the reaction mixture was stirred at 100 °C for 16 h. The reaction mixture was cooled to room temperature and water (30 mL) was added. The aqueous solution was extracted with ethyl acetate (2 × 50 mL). The pooled organic solution was dried with MgSO_4_, filtered and concentrated *in-vacuo*. The resulting crude biphenyl material was treated with 4 N HCl (2 mL) and stirred for 1 h. The reaction mixture was neutralized to pH 7 with saturated solution of sodium bicarbonate. The solution was extracted with ethyl acetate (3 times). The pooled organic solution was dried with MgSO_4_, filtered and concentrated *in vacuo*. The crude product was purified by column chromatography with column elution from 50% to 90% ethyl acetate in hexane, followed by preparative HPLC purification to **28** (18 mg, 0.031 mmol, 91% as a colorless liquid. ^1^H NMR (400 MHz, CDCl_3_) δ 8.02 (br s, 1H), 7.89 (d, *J* = 8.8 Hz, 2H), 7.65 (d, *J* = 8.8 Hz, 2H), 7.13 (d, *J* = 8.4 Hz, 2H), 7.08 (d, *J* = 8.4 Hz, 2H), 4.75–4.66 (m, 1H), 3.79–3.70 (m, 1H), 3.57–3.43 (m, 2H), 3.39 (s, 3H), 3.14–3.03 (m, 1H), 2.88–2.80 (m, 1H), 2.76–2.67 (m, 1H), 2.58–2.49 (m, 1H), 2.48–2.39 (m, 2H). HMRS (ESI) calculated for C_21_H_25_F_3_N_3_O_6_S^+^ [M + H]^+^ *m*/*z* 504.1411, found 504.1413. HPLC purity: 95.0% (RT: 7.65 min).

#### (R)-1-((4′-Chloro-[1,1′-biphenyl]-4-yl)­sulfonyl)-*N*-hydroxy-4-(2-methoxyethyl)­piperazine-2-carboxamide (**29**)

A pressure tube was charged with an aryl-bromide **26** (109 mg, 0.215 mmol), 1,4-dioxane (4 mL), tetrakis­(triphenylphosphine)­palladium(0) (8 mg, 0.0070 mmol), (4-chlorophenyl)­boronic acid (37 mg, 0.24 mmol) and aqueous 2 M K_2_CO_3_ (0.6 mL). The reaction mixture was heated at 80 °C for 16 h. Water was added and the mixture was extracted with ethyl acetate (3 times). The pooled organic solution was dried with MgSO_4_, filtered and concentrated *in-vacuo*. The crude product was purified by column chromatography following column elution from 50% to 90% ethyl acetate in hexane to give a biphenyl product (91 mg, 0.17 mmol, 79% as a colorless liquid. The resulting biphenyl material (87 mg, 0.16 mmol) was treated with 4 N HCl (2 mL in 1,4-dioxane) and the reaction mixture was stirred for 1 h. The reaction mixture was neutralized to pH 7 with saturated solution of sodium bicarbonate. The solution was extracted with ethyl acetate (3 times). The pooled organic solution was dried with MgSO_4_, filtered and concentrated *in-vacuo*. The crude product was purified by column chromatography following column elution of 10% methanol in DCM and subsequent semipreparative HPLC purification to give **29** (60 mg, 0.13 mmol, 81% as colorless liquid. ^1^H NMR (400 MHz, CDCl_3_) δ 8.00 (br s, 1H), 7.95 (d, *J* = 8.3 Hz, 2H), 7.66 (d, *J* = 8.3 Hz, 2H), 7.54 (d, *J* = 8.4 Hz, 2H), 7.44 (d, *J* = 8.4 Hz, 2H), 4.76–4.70 (m, 1H), 3.81–3.72 (m, 1H), 3.56–3.41 (m, 2H), 3.37 (s, 3H), 3.26–3.18 (m, 1H), 3.18–3.07 (m, 1H), 2.87–2.78 (m, 1H), 2.74–2.64 (m, 1H), 2.57–2.47 (m, 1H), 2.46–2.35 (m, 2H). HRMS (ESI) calculated for C_20_H_25_ClN_3_O_5_S^+^ [M + H]^+^ *m*/*z* 454.1198, C_20_H_24_ClN_3_NaO_5_S^+^ [M + Na]^+^ *m*/*z* 476.1017, C_40_H_48_Cl_2_N_6_NaO_10_S_2_^+^ [2M+Na]+ *m*/*z* 929.2143, found 454.1196, 476.1010, and 929.2136. HPLC purity: 96.7% (RT: 7.19 min).

### Molecular Modeling and Docking

Computational chemistry procedures can be found in the Supporting Information.

### Biology

Venoms were extracted from specimens of *E. romani* (Nigeria [formerly *E. ocellatus*]), *C. atrox* (United States of America) and *B. arietans* (Nigeria) maintained within the herpetarium at the Center for Snakebite Research and Interventions (CSRI) at the Liverpool School of Tropical Medicine (LSTM). Crude venoms were pooled by species and lyophilized for long-term storage at 2–8 °C. Venoms from *C. rhodostoma* (Thailand) and *B. jararaca* (Brazil) were sourced from the historical collection of lyophilized venoms held at LSTM. Venoms were reconstituted to 10 mg/mL in sterile phosphate buffered saline (PBS, pH 7.4) (cat. no. 10010-015, Gibco) prior to use.

### *In Vitro* Inhibition of Enzymatic SVMP Activity

Compounds of interest were stamped onto 384-well plates at a volume of 0.91 μL at a concentration of 1 mM in 100% DMSO (D2650, Sigma-Aldrich). Positive and negative control wells were stamped with 0.91 μL of 100% DMSO. To the test wells and the venom-only positive control wells, 15 μL of venom diluted to 0.066 μg/mL in PBS was added, giving a final amount of 1 μg/well. In the no venom negative control wells, 15 μL of PBS was added. The plates were then sealed, span briefly in a Platefuge (C2000, Benchmark Scientific) and incubated at 37 °C for 25 min to allow venom-inhibitor interaction. The plates were removed from the incubator for 5 min to allow acclimation to room temperature, then 75 μL of Mca-KPLGL-Dpa-AR-NH2 fluorogenic peptide substrate (ES010, BioTechne), diluted to a concentration of 9.1 μM in SVMP substrate buffer (150 mM NaCl, 50 mM Tris HCl pH 7.5), was added to every well using a ViaFlo 384 (Integra). The final assay volume was 91 μL, yielding a final drug concentration of 10 μM, a final DMSO concentration of 1%, and a final SVMP substrate concentration of 7.5 μM. The plates were then read kinetically using a fluorescence intensity protocol on a CLARIOStar microplate reader (BMG Labtech) at 320 nm excitation, 420 nm emission, for 60 min. At a time point appropriate for the venom to have cleaved all substrate in the well (*E. romani* and *C. rhodostoma* 20 min; *B. jararaca* and *C. atrox* 30 min; *B. arietans* 40 min), raw fluorescence values were normalized to be a percentage of the positive and negative control values, with complete inhibition of SVMP activity displaying as 100% inhibition and no inhibition of SVMP activity displaying as 0% inhibition. Dose response plates were conducted identically and the % inhibition values at each dose were used to draw EC_50_ curves to estimate the EC_50_ value of each compound tested. All SVMP experiments were repeated independently a minimum of three times.

### *In Vitro* Inhibition of Coagulopathic Venom Activity

This assay consists of preincubating venom (or venom vehicle control) at a species-specific dose with the compound of interest (or compound vehicle control), then adding citrated bovine plasma to the wells and activating the clotting cascade with calcium chloride. The selected venoms (*E. romani* and *B. jararaca*) are procoagulant, resulting in clot formation within 10 min compared to ∼30–45 min with recalcified normal plasma. As *E. romani* is a more potently procoagulant venom, only 10 ng per reaction was required to initiate rapid clotting, whereas 100 ng of *B. jararaca* venom was required to induce a similarly rapid clotting curve. Compounds of interest were stamped onto 384-well plates at a volume of 0.5 μL and a concentration of 10 mM in 100% DMSO. Positive and negative control wells were stamped with 0.5 μL of 100% DMSO. To the test wells and the venom-only positive control wells, 10 μL of venom diluted to 1 μg/mL (*E. romani*) or 10 μg/mL (*B. jararaca*) in PBS was added, giving a final well amount of 10 ng (*E. romani*) or 100 ng (*B. jararaca*) per well. In the no venom negative control wells, 10 μL of PBS was added. The plates were then sealed, span briefly in a Platefuge and incubated at 37 °C for 25 min to allow venom-inhibitor interaction. While the plates were incubating, sufficient citrated bovine plasma (S0260, BioWest) to allow 20 μL/well was centrifuged at 3120 RCF for 5 min to remove any residual cellular debris and the supernatant was collected. The 384-well plates were removed from the incubator for 5 min to allow acclimation to room temperature, then 20 μL/well of 20 mM CaCl_2_ was added and immediately followed by the addition of 20 μL/well of centrifuged citrated bovine plasma, both via a Multidrop Combi reagent dispenser (5840330, Thermo Fisher Scientific). The plates were then read kinetically using an absorbance protocol on a CLARIOStar microplate reader at 595 nm for 60 min. The time, in minutes, required to achieve half the maximal absorbance value was calculated for each well using Prism (GraphPad, v10.2.3), and then were normalized to be a % of the positive and negative control values, with complete inhibition of procoagulant activity displaying as 100% inhibition and no inhibition of procoagulant activity displaying as 0% inhibition. Dose response plates were conducted identically and the % inhibition values at each dose were used to draw EC_50_ curves to estimate the EC_50_ value of each compound tested. All coagulation experiments were repeated independently a minimum of three times.

### Degradation of Coagulation Factors

Evaluation of SVMP toxin activity of human prothrombin and fibrinogen and inhibition by test compounds was performed by SDS-PAGE degradation gel electrophoresis using venom-purified toxins. P–I, P–II and P–III SVMPs were isolated using an adaptation of the method of Howes et al.^42^ Whole *E. romani* venom (15 mg) was initially separated using size exclusion chromatography (SEC) on a 120 mL column of Superdex 75 (Cytiva). The P–II SVMP (22 kDa) was pure and ready to use at this stage. P–I and P–III SVMP containing peaks were separately applied to a 1 mL high resolution hydrophobic interaction chromatography (HIC) column (SOURCE 15PHE, Cytiva). The proteins were separated using a 10-column volume gradient of 1.0 M ammonium sulfate in 50 mM Tris-Cl, pH 8.8 to 25% ethylene glycol in 50 mM Tris-Cl, pH 8.8. The P–I eluted as two peaks of proteins with identical molecular weight (21 kDa) which were combined back together. The P–III-containing fraction from SEC eluted from HIC as multiple peaks of P–III SVMP isoforms, well separated from the earlier eluting L-amino acid oxidase and high molecular weight C-type lectin-like proteins. The proteins in the PIII-containing peaks were pooled back together. Finally, both the P–I and P–III SVMPs were dialyzed into TBS (25 mM Tris-Cl, pH 7.8, 0.15 M NaCl) ready for degradation assays.

Prothrombin degradation was measured by incubating 2 μg of human prothrombin (Hematologic Technologies INC) in the presence or absence of drug and the P–I, P–II, and P–III SVMPs. As the P–II SVMP did not cleave the prothrombin substrate, it was excluded from inhibitory assaying. Briefly, drug or DMSO and 200 ng of purified SVMP isoform were incubated for 30 min at 37 °C in a water bath, after which 2 μg of human prothrombin were added and the samples incubated for another hour at 37 °C. Drug concentrations used were 2 μM for P–I and P–III SVMPs. Samples were then processed for loading onto a 4–20% SDS-PAGE gel (Biorad) and were run under reducing conditions for 45 min at 180 V. The gels were stained with Coomassie Brilliant Blue R250 and degradation profiles were visualized and images captured using a GelDoc (Biorad).

Degradation of human fibrinogen was measured in a similar manner. Human fibrinogen (Merck) was added at 5 μg/reaction and incubated with the different SVMP isoforms in the presence or absence of drugs. Drug concentrations used were 10 μM for P–I and P–II SVMP and 400 nM for P–III SVMP. The reactions consisted of 200 ng SVMP isoform and the appropriate concentration of drug, which were then incubated for 30 min at 37 °C in a water bath. Five micrograms of fibrinogen were then added and the samples incubated for another hour at 37 °C, before separation by SDS-PAGE as described above.

### Thromboelastography

We measured the coagulation profile of whole human blood using thromboelastography, and compared with venom stimulated, and venom and drug stimulated, clotting profiles. Blood samples were obtained according to ethically approved protocols (LSTM research tissue bank, REC ref 11/H1002/9) from consenting healthy volunteers who confirmed they had not taken any anticoagulant treatments for at least three months prior to blood collection. Blood samples were collected in ACD-A blood tubes. Using a ROTEM delta (Werfen) a final volume of 300 μL blood per reaction was pipetted into prewarmed cups (37 °C), with four channels per run. Whole blood was warmed for 5 min at 37 °C, during which time 12 μL of *E. romani* venom (5 ng/nL concentration, 60 ng dose), 15 μL of DMSO vehicle or test compounds (5 μM final concentration, 0.2% DMSO), and 20 μL of Startem reagent (CaCl_2_, Werfen) was prepared. The maximum incubation time of venom and inhibitors was 2 min, before 253 μL of whole blood were added and mixed before reading on the ROTEM delta. The commonly used parameters of clotting time and maximum clot firmness (MCF) were reported, from a minimum of three independent measurements per experimental group.

### Chicken Egg Embryo Model

To compare the *in vivo* efficacy of **23** (DC-174) with **1f** (XL-784), a chicken embryo model was applied.^35^ Fertile hens eggs were supplied on day 1 post fertilization from UK supplier MedEggs Ltd. Eggs were immediately placed horizontally to incubate at 37 °C until day 5 of development. Candling was conducted to mark the position of the embryo to guide windowing. Any infertile eggs were removed at this stage. To access the embryos, all fertile eggs were then windowed under laminar flow. In brief, 6–8 mL of albumin was removed using a 23G needle. The area marking the position of the embryo was then covered with medical tape, and a dish of shell removed using sharp dissection scissors. The exposed embryo was then covered using parafilm and the egg returned to the incubator overnight. Surviving embryos were then randomly allocated into groups of *n* = 5. Embryos then received either *E. romani* venom 10 μg/egg or *E. romani* venom immediately followed by XL-784 or DC-174 (1 μg/egg). Treatments were given in a total volume of 10 μL, administered directly onto (i.e., topically) the vitelline vein. Embryos were returned to the incubator immediately following dosing, and then observed at 1, 2, 4, and 6 h. Survival was monitored via the observation of a heartbeat. Venom pathology was captured using a Motic SMZ 171-TLED microscope system. In brief, at each observation time point (1, 2, 4, and 6 h post envenoming) images were captured using a Moticam X5 Wi-Fi camera. To track changes across the experimental time course, the imaging position was kept consistent at each time point for individual embryos.

### *In Vivo* Protection against Venom-Induced Lethality

Murine preclinical studies were conducted under protocols approved by Animal Welfare and Ethical Review Boards of the Liverpool School of Tropical Medicine and University of Liverpool under project license (PPL No. P5846F90) approved by the UK Home Office in accordance with the UK (Scientific Procedures) Act 1986. Male CD-1 mice (18–20 g) were purchased from Janvier (France) and acclimatized for 7 days prior to experimentation. Mice were housed in groups of 5 in Tecniplast GM500 cages within a specific pathogen-free facility. Room conditions were set at 20–24 °C and 45–65% humidity, 12/12 h light cycles. Mice were allowed ad libitum access to Picolab 5R53 food (Lab Diet, USA) and reverse osmosis water and housed with nesting material and environmental enrichment materials.

Mice weighed 22–28 g at the start of study. Five milligram or kilogram morphine was administered subcutaneously as an analgesic 15 min prior to venom administration. To determine the efficacy of **23** (DC-174), a modified version of the WHO neutralization assay was used.^43^ Following analgesia, mice received an intraperitoneal injection of 90 μg *E. romani* venom in 100 μL PBS, corresponding to 5× the intravenous LD50 dose.^44^ DC-174 was solubilized in SSV (carboxymethyl cellulose (0.5%, benzyl alcohol (0.5%, Tween 80 (0.4% v/v) and NaCl (0.9%) and gently sonicated at 24 °C for 5 min. Mice received an oral dose of either 20 mg/kg **23** (DC-174) in SSV, or SSV-only (*n* = 5/group, 100 μL/dose), immediately following venom administration. A third group received venom followed by two oral doses of **23** (DC-174), one immediately after venom administration and a second identical dose 1.5 h later. No randomization was used to allocate experimental groups – mice were randomly allocated into cages of five prior to the experiment, and each cage formed one treatment group. No criteria for including or excluding animals was applied, and all data points were included in analyses. Animals were monitored for humane end points (loss of righting reflex, seizure, external hemorrhage) for 8 h to encompass the drug exposure time frame, and any animals showing such signs were immediately euthanized by rising concentrations of carbon dioxide. All observations were performed by mixed gender experimenters who were blinded to the drug group allocation. Time of death, number of deaths and number of survivors were recorded, where deaths and times of death represent implementation of humane end point-dictated euthanasia. Kaplan–Meier survival plots were generated using GraphPad Prism 9.0 (GraphPad Software, San Diego, USA).

### Envenoming Biomarker Analysis

Blood samples were collected from mice via cardiac puncture immediately following euthanasia. Samples were centrifuged at 1400 × *g* for 10 min at 4 °C, and the plasma supernatant stored at −80 °C until use. Plasma TAT levels were determined using a sandwich ELISA provided as a commercial kit (Abcam, ab137994). Mouse plasma samples were diluted 1:150 using Diluent M and were run in duplicate alongside a standard curve. Each well received 50 μL diluted plasma and was incubated for 2 h at room temperature to allow for the reaction with the TAT complexes antibodies coated onto the plate. After five washes, 50 μL of biotinylated mouse TAT complexes antibodies were added to the plate and the reaction allowed to proceed for another 2 h. Following another five washes, 50 μL of a streptavidin-peroxidase conjugate were added to the plate and incubated for another 30 min. Next, 50 μL of a chromogenic substrate (3,3′,5,5′-tetramethylbenzidine) was added to the plate and the peroxidase-dependent reaction allowed to proceed until a clear blue color formed in the well. At this point 50 μL of stop solution was added and the absorbance read immediately at 450 and 570 nm. The 570 nm absorbance readings were subtracted from the 450 nm ones, and the levels of TAT complexes (ng/mL) extrapolated based on the standard curve. Data was further plotted as means with SD for each mouse sample and *n* = 4–5 samples were used for each experimental condition.

Antifibrinogen and antiprothrombin Western blots were performed using mouse plasma to detect the integrity of mouse fibrinogen or prothrombin following envenoming in the presence or absence of **23** (DC-174). Mouse plasma samples were diluted 1:50 (antifibrinogen blots) and 1:30 (antiprothrombin blots) and 5 μL was run on 4–20% SDS-PAGE gels (BioRad) at 180 V for 1 h. The separated proteins were then transferred onto nitrocellulose membranes using semidry transfer on a Trans-Blot Turbo (BioRad) using the following conditions: 1.3 A, 25 V, 7 min. Blots were then stained for 5 min with Revert 700 Total Protein Stain (LICORbio) following the manufacturer’s protocol and imaged on the 700 nm channel on an Odyssey Fc Imaging System to detect protein abundance and ensure equal loading. Membranes were then destained in 0.1 M NaOH, 30% methanol for no longer than 10 min, then blocked in 5% milk TBST for 1 h. The primary antibodies: antifibrinogen (ab34269, rabbit polyclonal, Abcam) and antiprothrombin (PA5-77976, rabbit polyclonal, Thermo Fisher) were diluted 1:1,500 and 1:1,000, respectively, in 5% milk TBST. The membranes were incubated with the appropriate primary antibody for 1.5 h at room temperature, followed by three 5 min washes in TBST. Blots were then incubated for 1 h at room temperature with an IRDye 800CW donkey anti-rabbit IgG secondary antibody (1:15000, LICORbio), followed by three 5 min washes in TBS. The blots were imaged on an Odyssey Fc Imaging System at both 700 and 800 nm.

### Drug Metabolism Pharmacokinetic Studies (DMPK) Properties Assays

The DMPK properties data described in the manuscript were measured via a high-throughput platform by AstraZeneca (United Kingdom). The methods used for the five assays, including logD_7.4_, solubility, plasma protein binding, microsomal and hepatocyte clearance measurements, have previously been reported.^45^

### *In Vivo* Pharmacokinetic (PK) Studies in Mice

The *in vivo* PK study was performed by ChemPartner (Shanghai, China). The study protocol is summarized as followed: The PK of **23** (DC-174) was studied in CD1 male mice (28–29 g, 6–8 weeks) purchased from JiHui Laboratory Animal Co. Ltd. (Qualification No.: SCXK (Hu) 2022–0009 20220009018616). Mice had access to food and water throughout the pre- and postdose sampling period. Compound DC-174 was formulated in 0.5% MC (Sigma, SLCL7954) and 0.1% Tween 20 (Sigma, BCCF2036) in water (RephiLe/S21RDB0304) and administered at 20 mg/kg via oral gavage (*N* = 9). Approximately 110 μL whole blood/time point (sampling at 0.083, 0.25, 0.5, 1, 2, 4, 8, and 24 h) was collected via serial bleeding in K_2_EDTA tube via facial vein. The blood sample was kept on ice and centrifuged (2,000 × *g*, 4 °C, 5 min) within 15 min postsampling. The plasma drug concentrations were determined by LC-MS/MS (ESI, Triple Quad 5500 System) on positive ion mode and using dexamethasone as internal standard. Liquid chromatography (LC) was performed using a 2.5 μm Xbridge BEH-C18 column (2.1 mm × 50 mm) at 60 °C with a rate of 0.6 mL/min. The following mobile phases were used; A: Water (+0.025% Formic acid and 1.0 mM ammonium acetate), B: Methanol (+0.025% Formic acid and 1.0 mM ammonium acetate). The LC method used was 5% B for 0.20 min, linear gradient from 5% to 60% B from 0.20 to 1.40 min, linear gradient from 60% to 65% B from 1.40 to 2.00 min, then hold 90% B from 2.01 to 2.30 min, followed by re-equilibration at 5% from 2.31 to 2.60 min.

### Statistical Information

For EC_50_ data, experiments were performed as independent (biological) repeats at least in triplicate with two technical repeats per biological repeat. EC_50_ curves were generated in Graphpad Prism on data normalized to the positive and negative controls (100% inhibition positive control/0% inhibition negative control) using the linear regression function. Ranges were calculated as mean ± SEM. Ordinary one-way ANOVAs with Tukey’s multiple comparisons test were performed for ROTEM thromboelastography data and TAT ELISA data, with statistical outputs reported in the relevant figure legends. All error bars represent the mean ± SD.

## Supplementary Material







## Notes

Supporting Information file containing compound analytical data, supplementary figures, additional computational studies, and X-ray crystallography data of DC-174 (PDF)
Molecular formula strings (CSV)
PDBs of homology modeling (ZIP)

## Abbreviations

WHO: World Health Organisation
SVMP: Snake venom metalloproteinase
SVSP: Snake venom serine proteinase
PLA_2_: Phospholipase A2
3FTX: Three-finger toxin
DMPS: 2,3-Dimercapto-1-propanesulfonic acid
MMP: Matrix metalloproteinase
HTS: High-throughput screening
DMPK: Drug metabolism and pharmacokinetics
SAR: Structure–activity relationship;
PK: Pharmacokinetic
ERO: *Echis romani*
BJA: *Bothrops jararaca*
BAR: *Bitis arietans*
CAT: *Crotalus atrox*
CRH: *Calloselasma rhodostoma*
TFA: Trifluoroacetic acid
NEt_3_: Triethylamine
DMAP: Dimethylaminopyridine
EDC: 1-Ethyl-3-(3-(dimethylamino)­propyl)­carbodiimide
HOBt: Hydroxybenzotriazole
NMM: *N*-Methylmorpholine
OTHP: O-(Tetrahydropyran-2-yl)­hydroxylamine
NH_2_OH: Hydroxylamine
KOCN: Potassium cyanate
MCF: Maximum clot firmness
PBS: Phosphate-buffered saline
SSV: Standard suspension vehicle
TAT: Thrombin–antithrombin
VICC: Venom-induced consumption coagulopathy
ANOVA: Analysis of variance
*T*_1/2_: Half-life
SDS-PAGE: Sodium dodecyl sulfate-polyacrylamide gel electrophoresis
TBS: Tris-buffered saline
ROTEM: Rotational thromboelastrometry
TBST: Tris-buffered saline with Tween 20
