# Supplementary material for: Identification of DC-174 as a Novel Hydroxamic Precandidate for the Development of an Oral Snakebite Treatment
Source: J Med Chem. 2026 Apr 9;69(8):9386–405. doi: 10.1021/acs.jmedchem.6c00064 (PMC13126677; doi:10.1021/acs.jmedchem.6c00064)
Supplement: Supplementary file 1 [file jm6c00064_si_001.pdf]

Supporting Information

Identification of DC-174 as a Novel Hydroxamic Pre-Candidate for the Development of an Oral Snakebite Treatment

Daniel J.W. Chong[1,#], Laura-Oana Albulescu[2,#], Adam Westhorpe[2,#], Rachel Clare[2], Amy Marriott[2], Christopher M. Woodley[1], Ramachandran Gunasekar[1], Nada Mosallam[1], Edouard Crittenden[2], Emma Stars[2], Charlotte Dawson[2], Jeroen Kool[3], Mark Wilkinson[2], Suet C. Leung[1], Neil G. Berry[1], Nicholas R. Casewell[2]*, & Paul M. O'Neill[1]*.

[1]Department of Chemistry, University of Liverpool, Grove Street, Liverpool, L69 7ZD, UK.

[2]Centre for Snakebite Research & Interventions, Liverpool School of Tropical Medicine, Pembroke Place, Liverpool, L3 5QA, UK.

[3]Department of Chemistry and Pharmaceutical Sciences, Amsterdam Institute for Molecular and Life Sciences, Vrije Universiteit Amsterdam, De Boelelaan 1105, Amsterdam 1081 HV, the Netherlands.

[#] These authors contributed equally

* Corresponding authors

Paul M. O'Neill – Department of Chemistry, University of Liverpool; Grove Street, Liverpool, L69 7ZD, United Kingdom; Email: P.M.Oneill01@liverpool.ac.uk, ORCID: 0000-0003-4338-0317

Nicholas R. Casewell – Centre for Snakebite Research & Interventions, Liverpool School of Tropical Medicine; Pembroke Place, Liverpool, L3 5QA, United Kingdom; Email: Nicholas.Casewell@lstmed.ac.uk, ORCID: 0000-0002-8035-4719

	Table of Contents	Page
1.	Analytical Data/ NMR	S2
2.	HPLC traces of the samples	S13
3.	Supplementary Figures	S25
4.	Molecular Modelling and Docking	S31
5.	Supplementary Computational Studies	S32
6.	X-ray Crystallography data of DC-174	S38
7.	Supplementary References	S40

Analytical Data/ NMR

^{1}H NMR spectrum of **15**

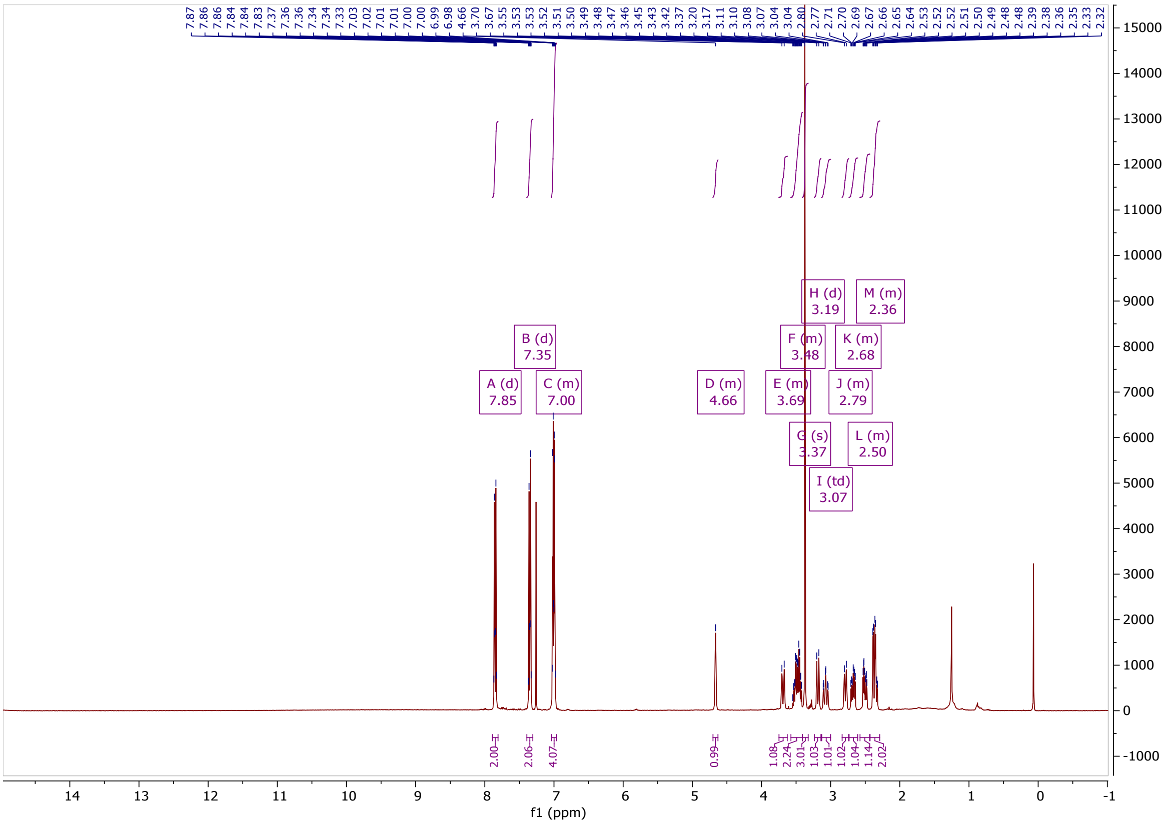

^{13}C NMR spectrum of **15**

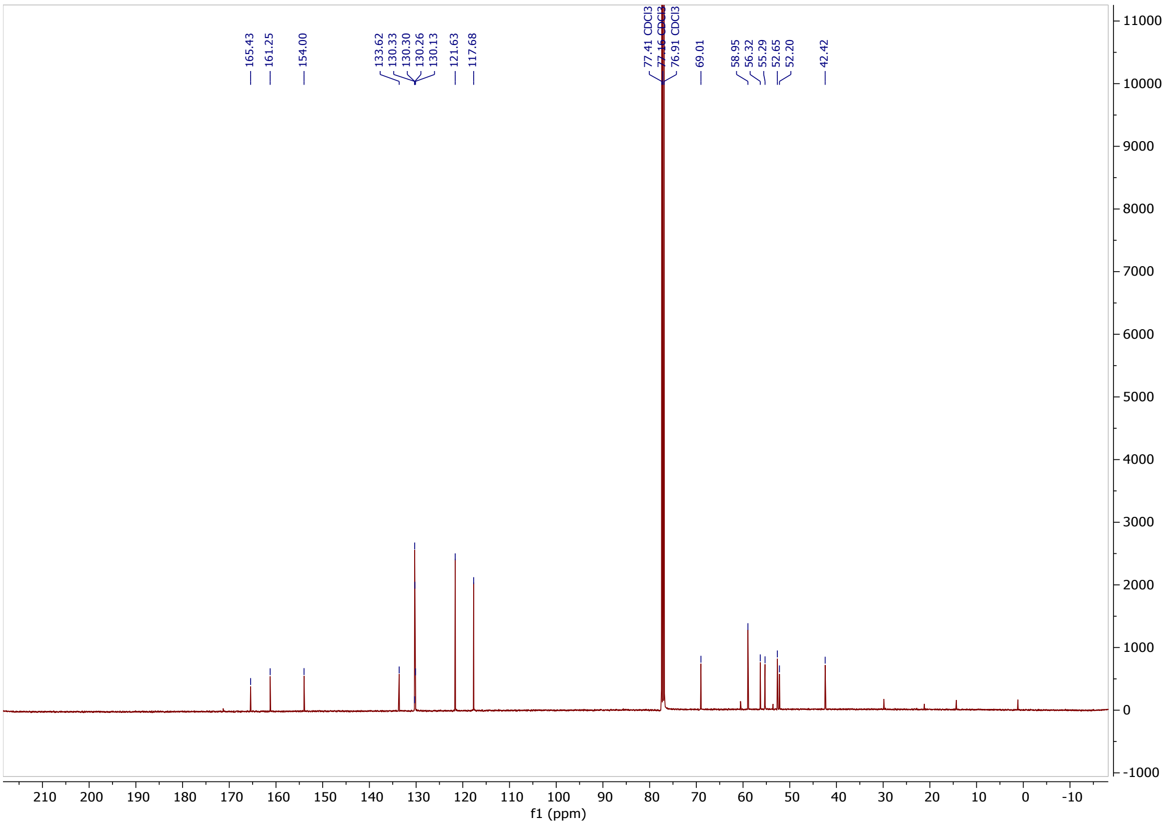

^{1}H NMR spectrum of **16**

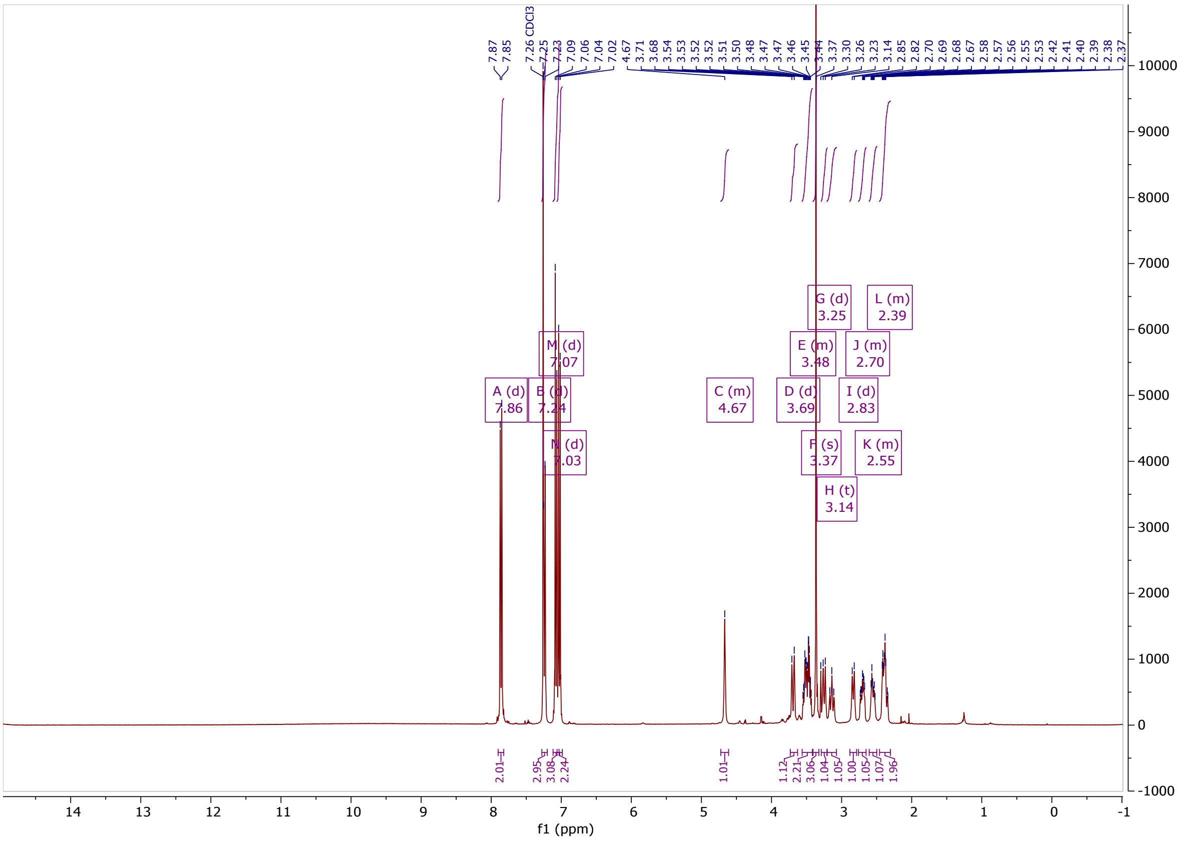

^{13}C NMR spectrum of **16**

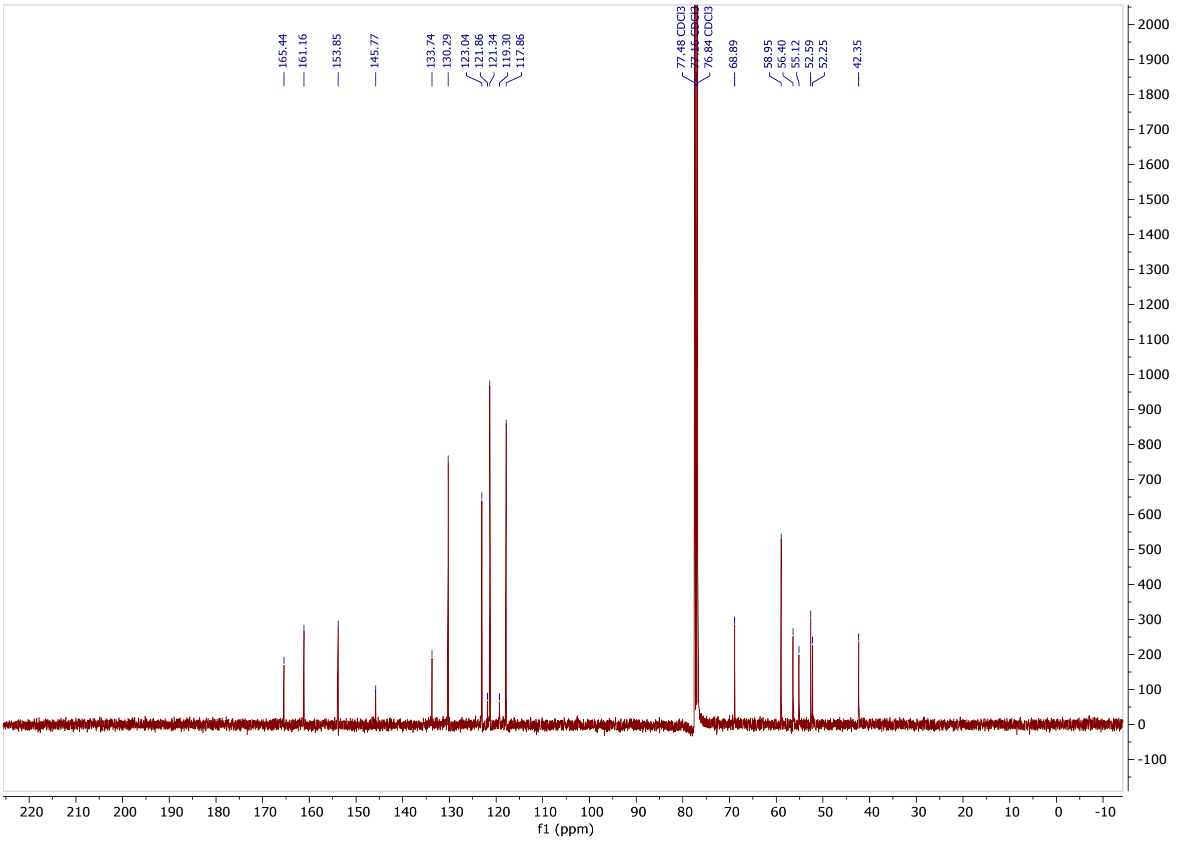

^{1}H NMR spectrum of **17**

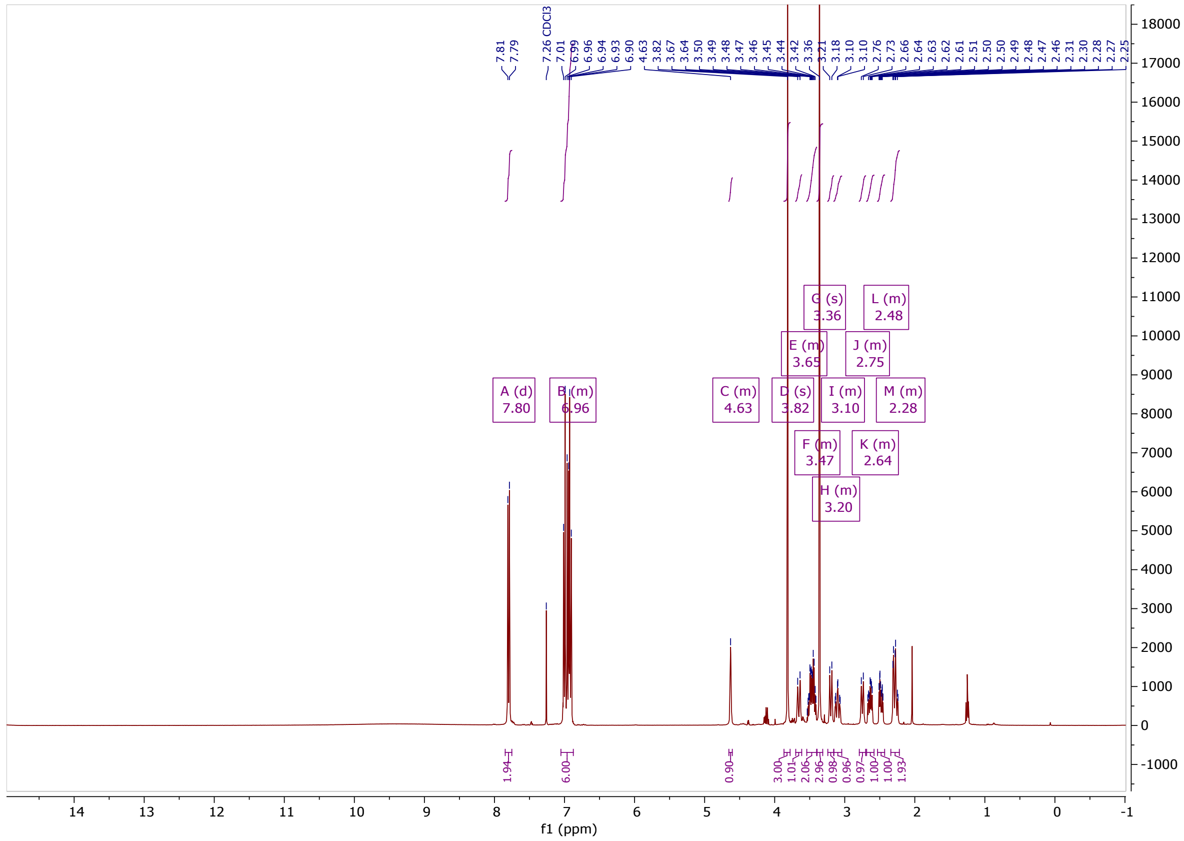

^{13}C NMR spectrum of **17**

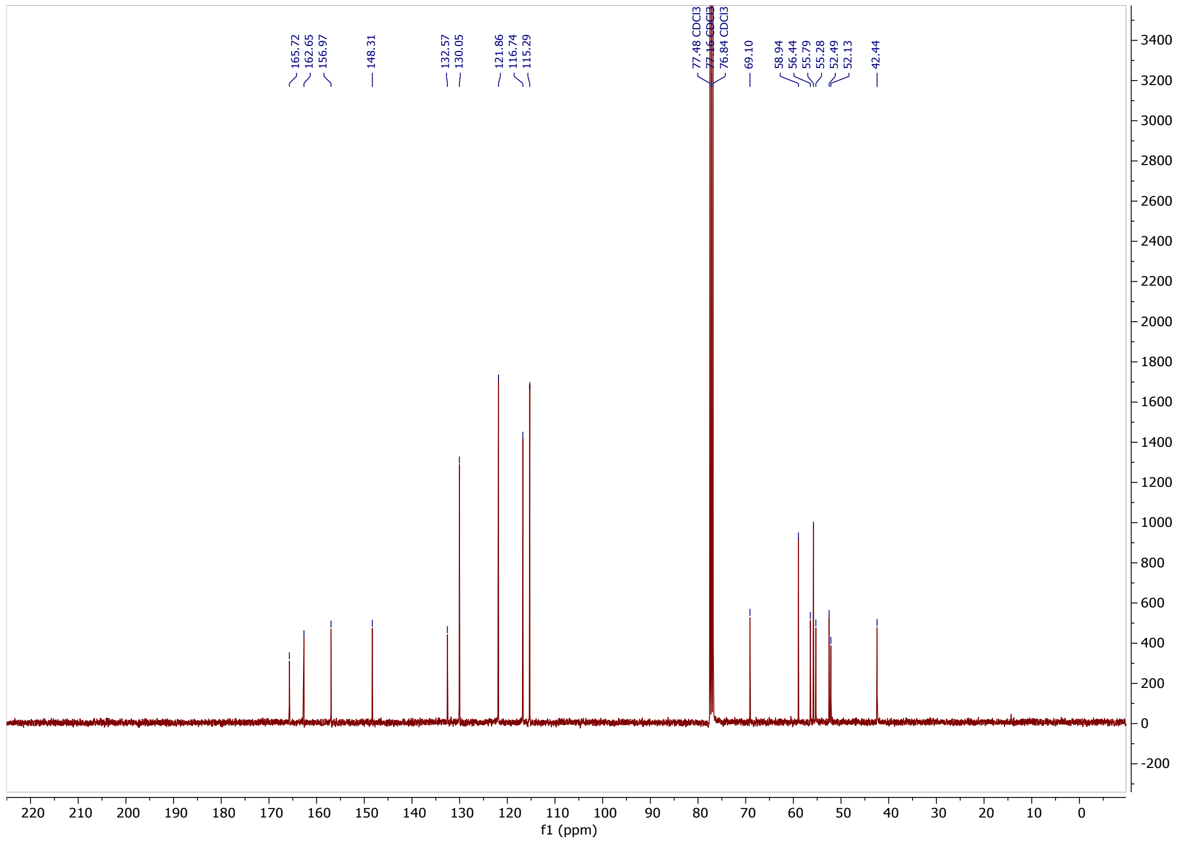

^{1}H NMR spectrum of **18**

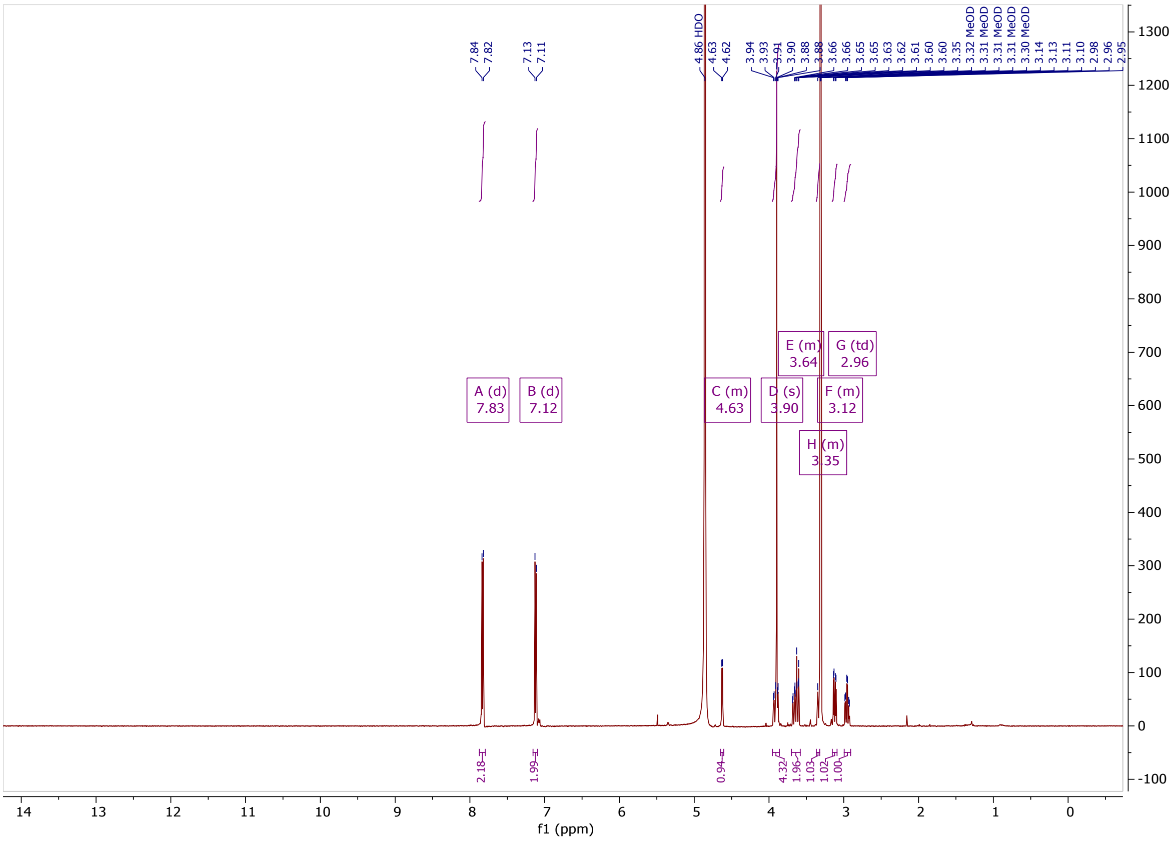

^{13}C NMR spectrum of **18**

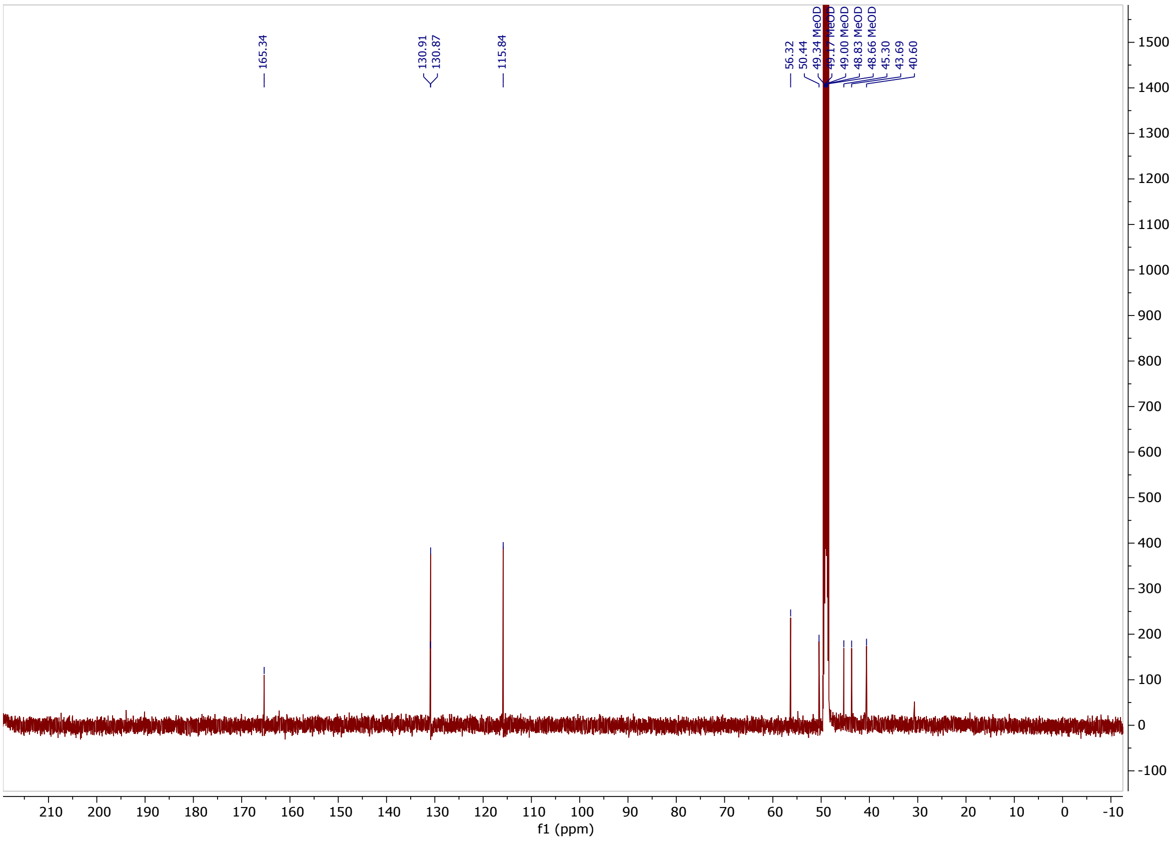

^{1}H NMR spectrum of **19**

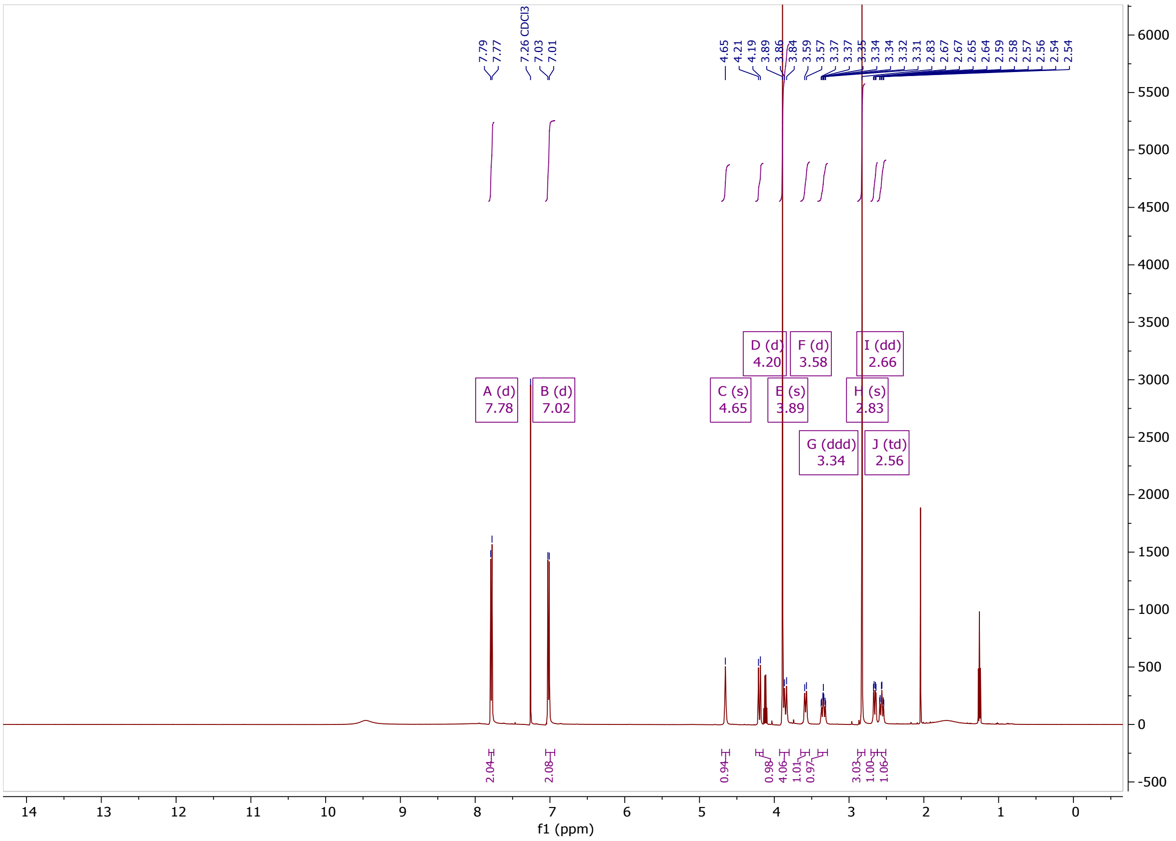

^{13}C NMR spectrum of **19**

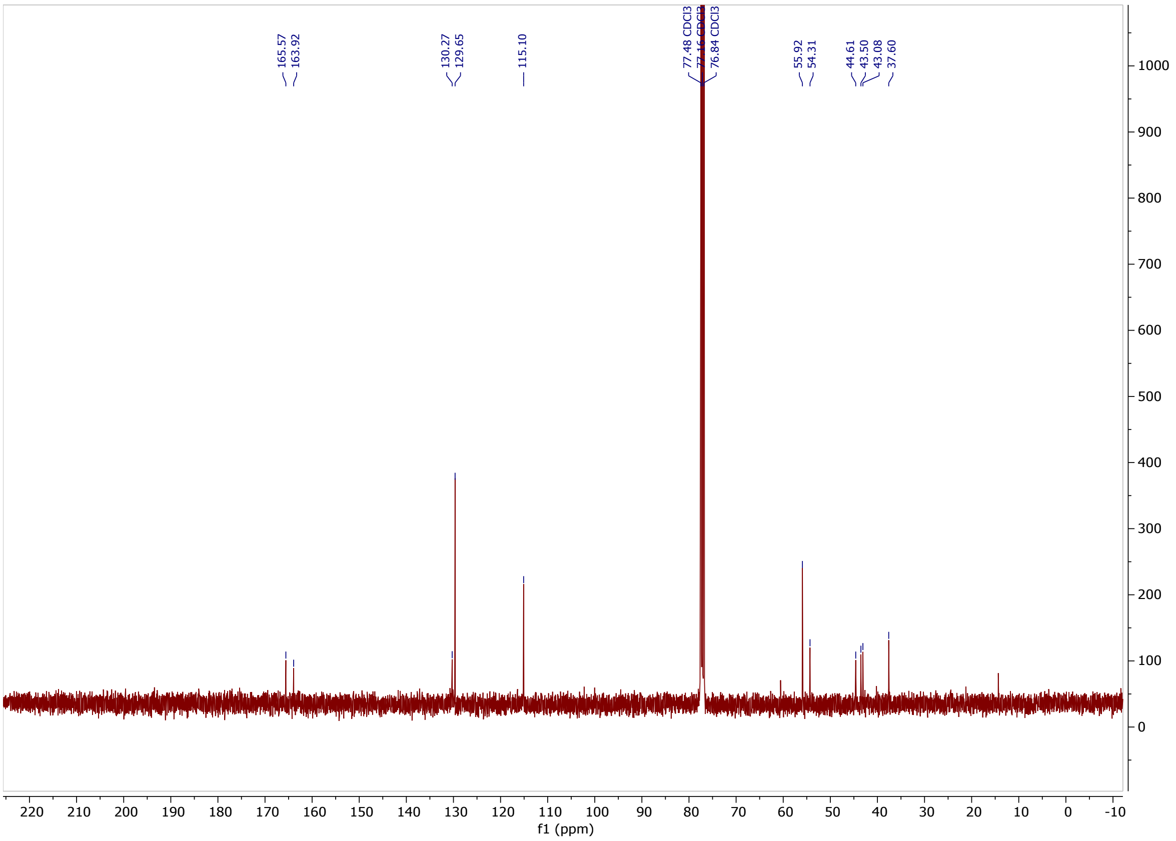

^{1}H NMR spectrum of **20**

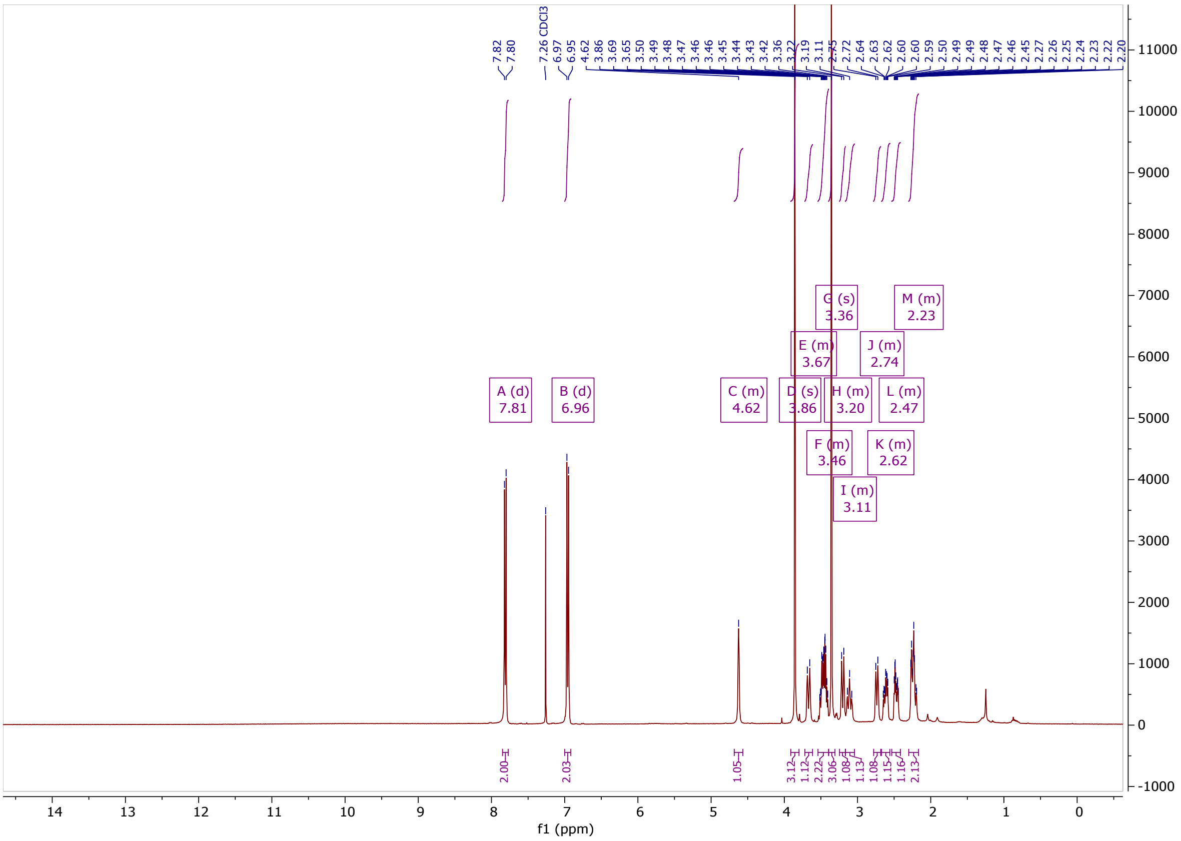

^{13}C NMR spectrum of **20**

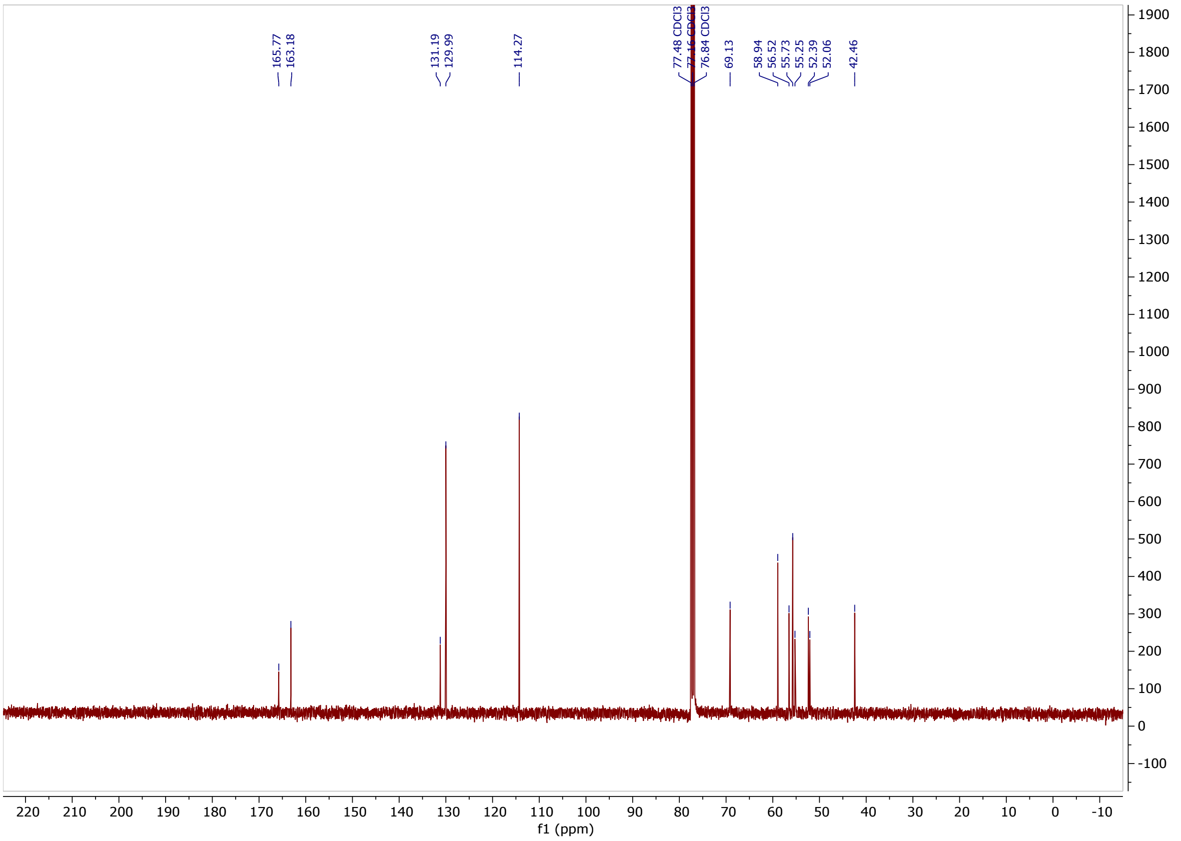

^{1}H NMR spectrum of **21**

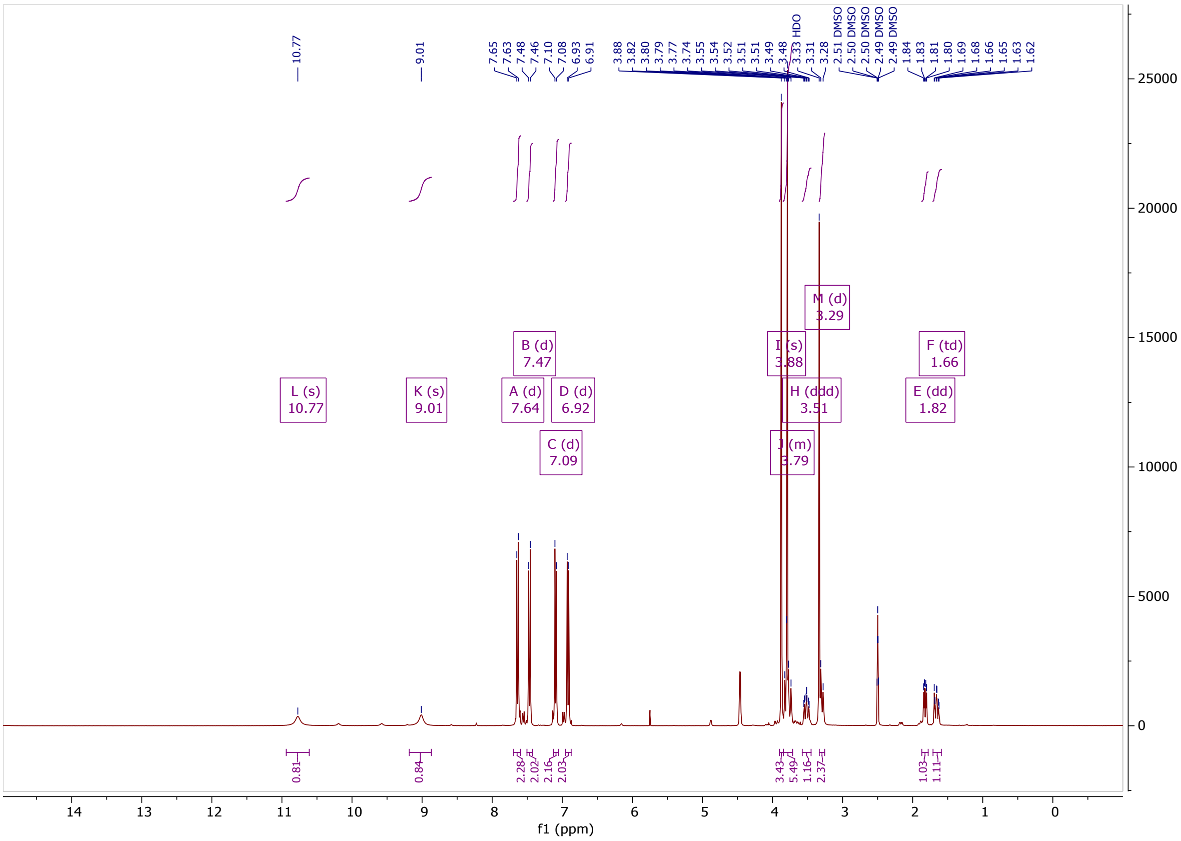

^{13}C NMR spectrum of **21**

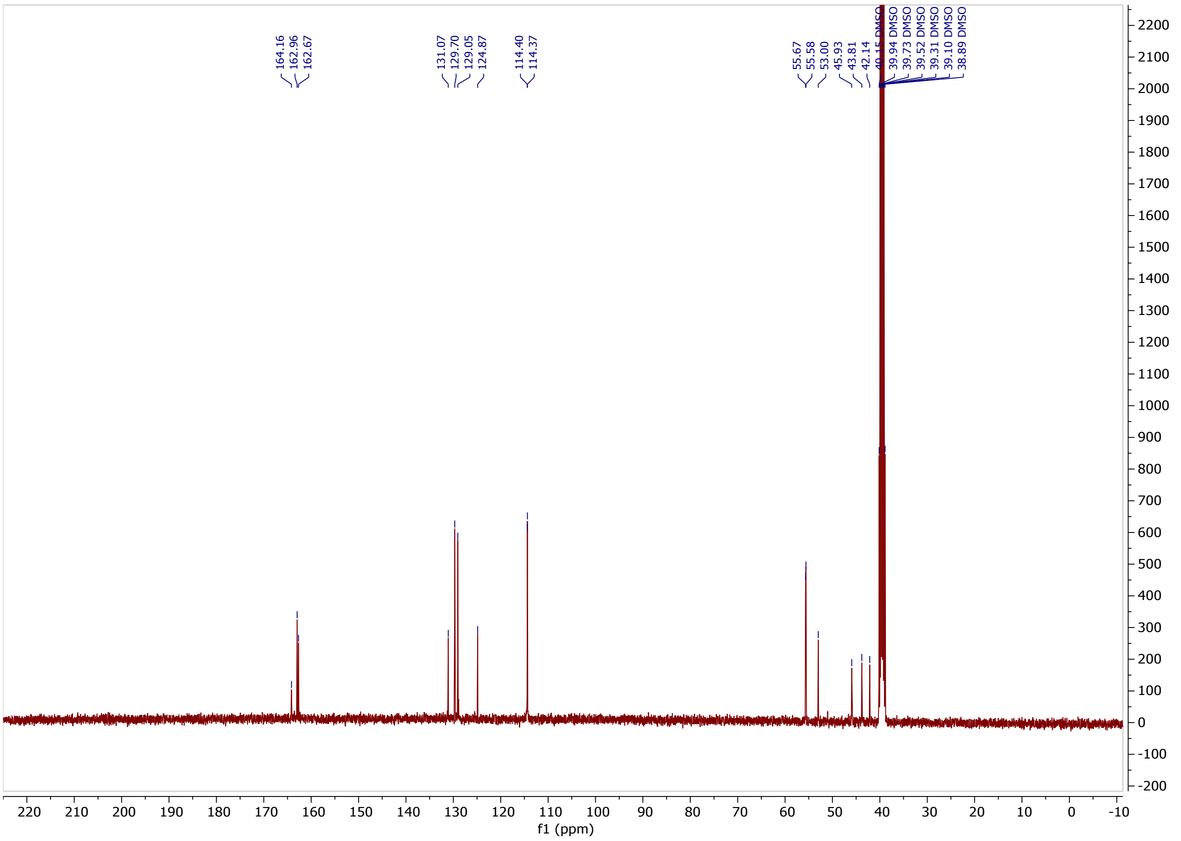

^{1}H NMR spectrum of **22**

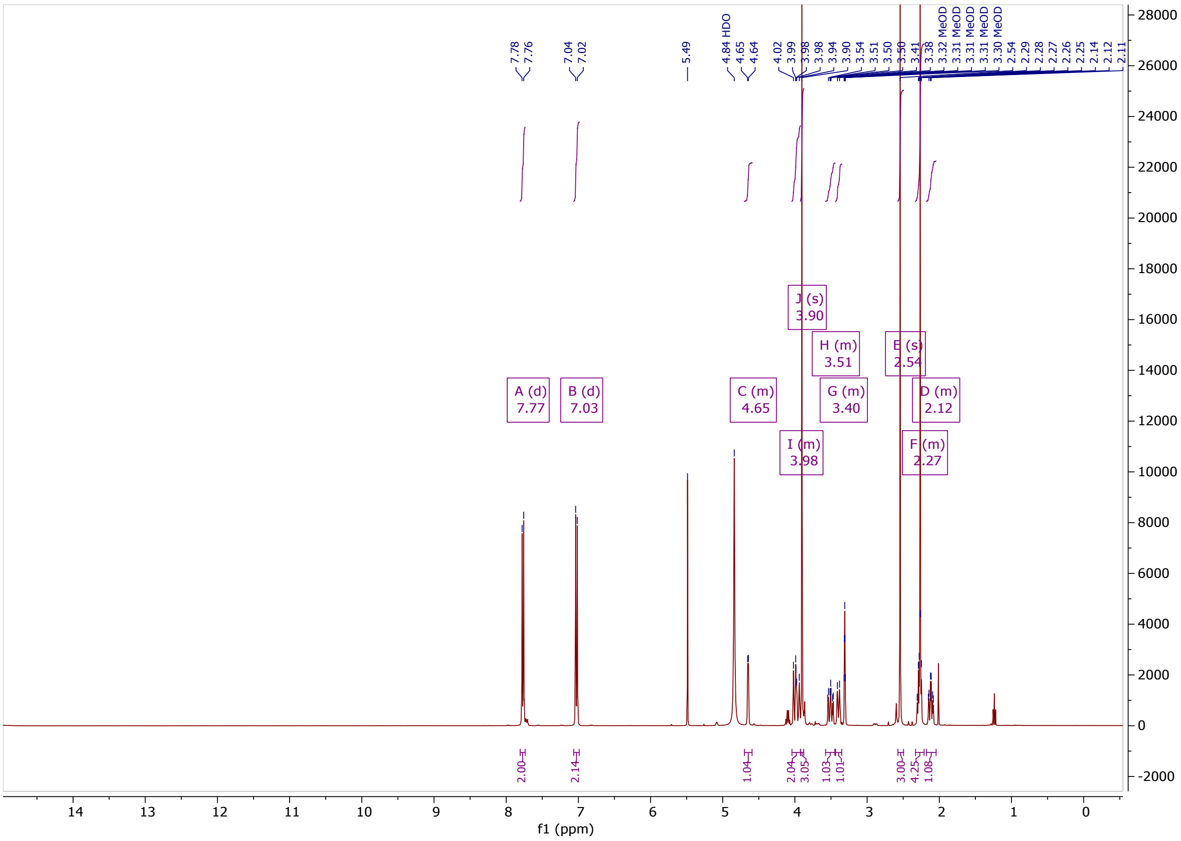

^{13}C NMR spectrum of **22**

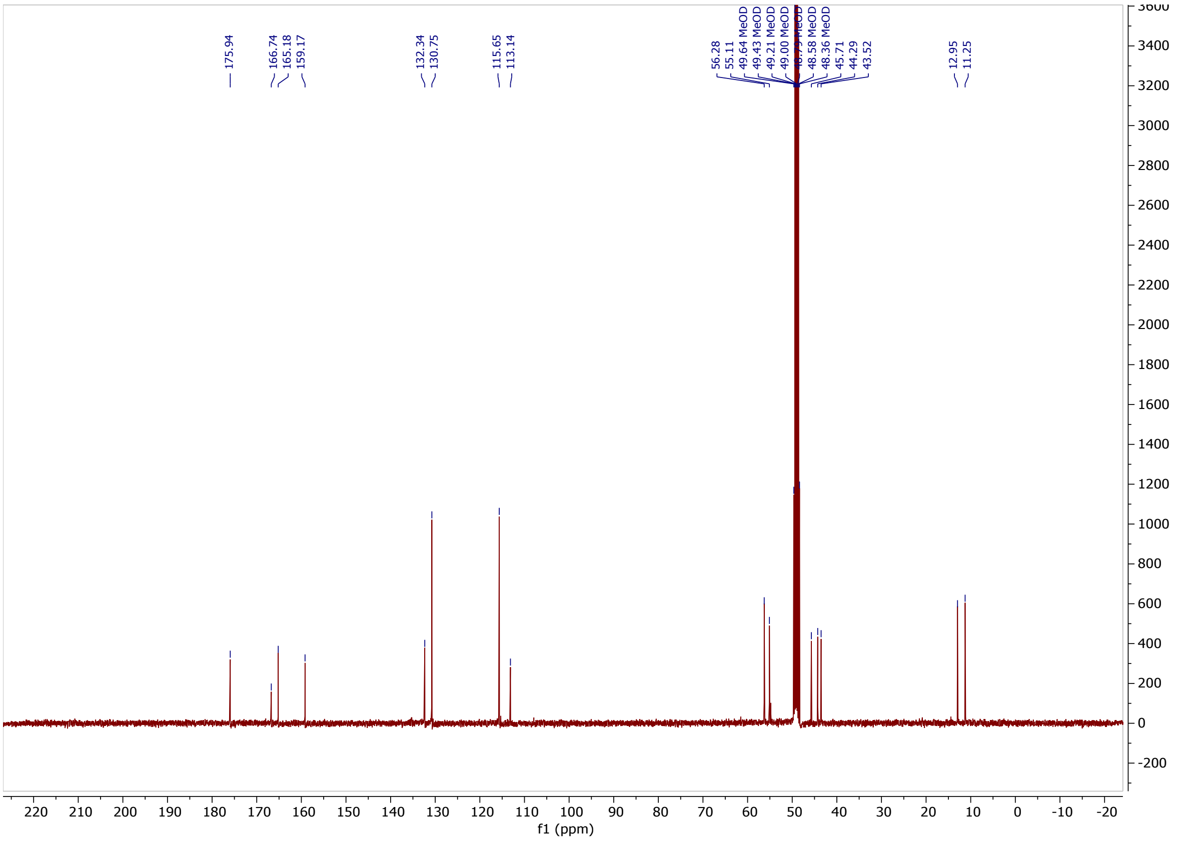

^{1}H NMR spectrum of **23** (DC-174)

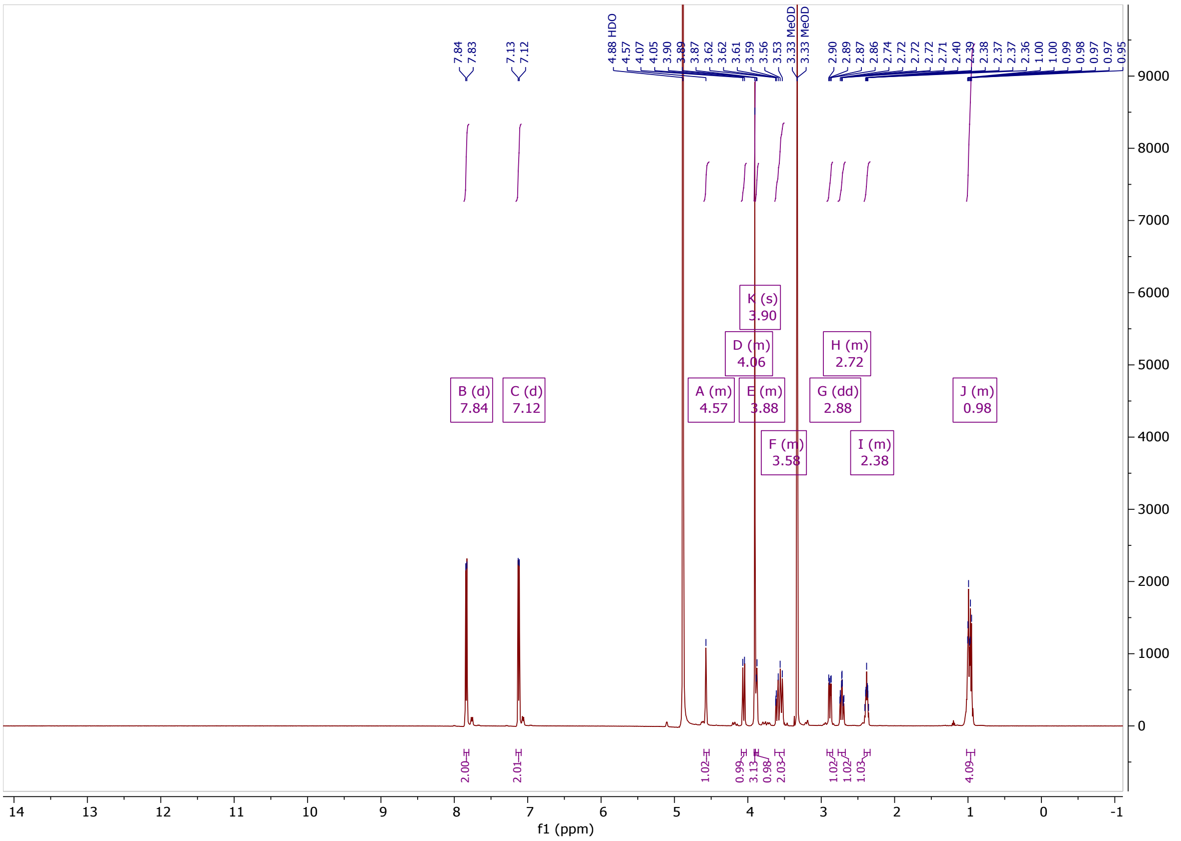

^{13}C NMR spectrum of **23** (DC-174)

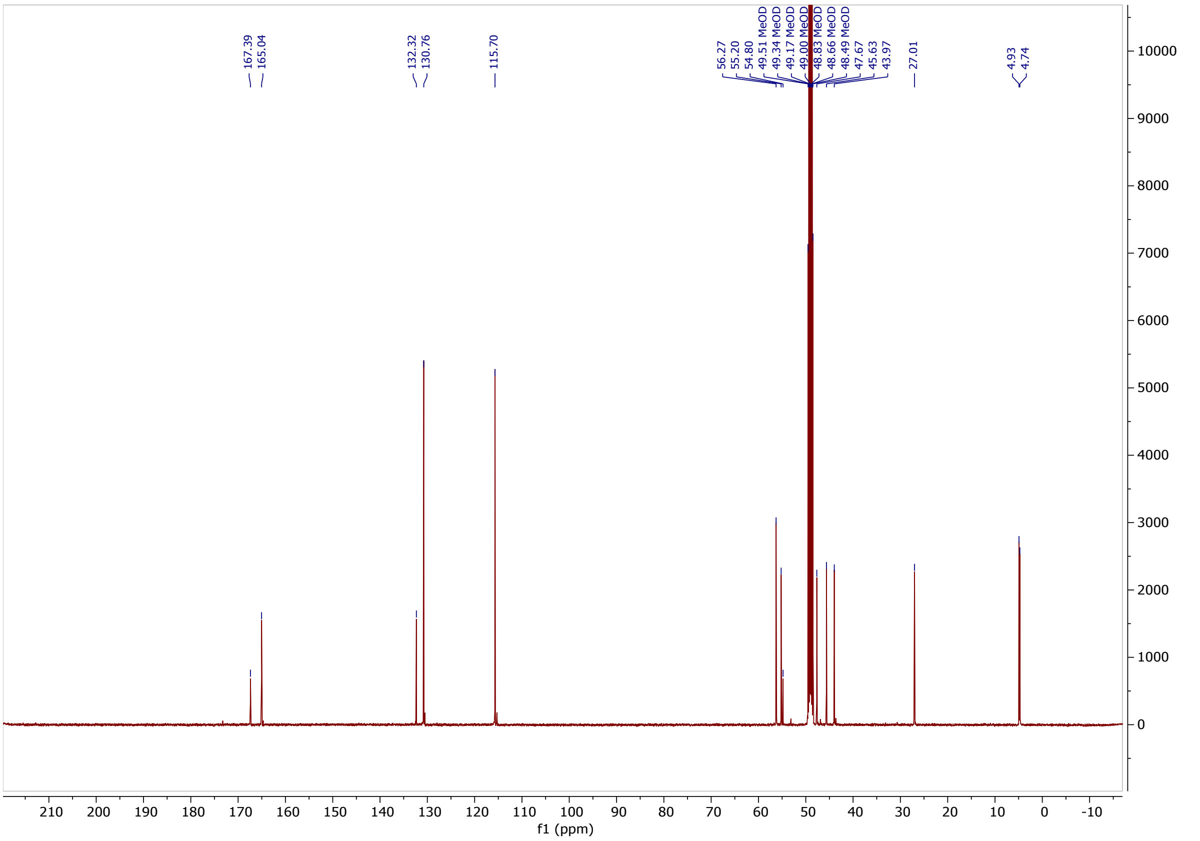

^{1}H NMR spectrum of **28**

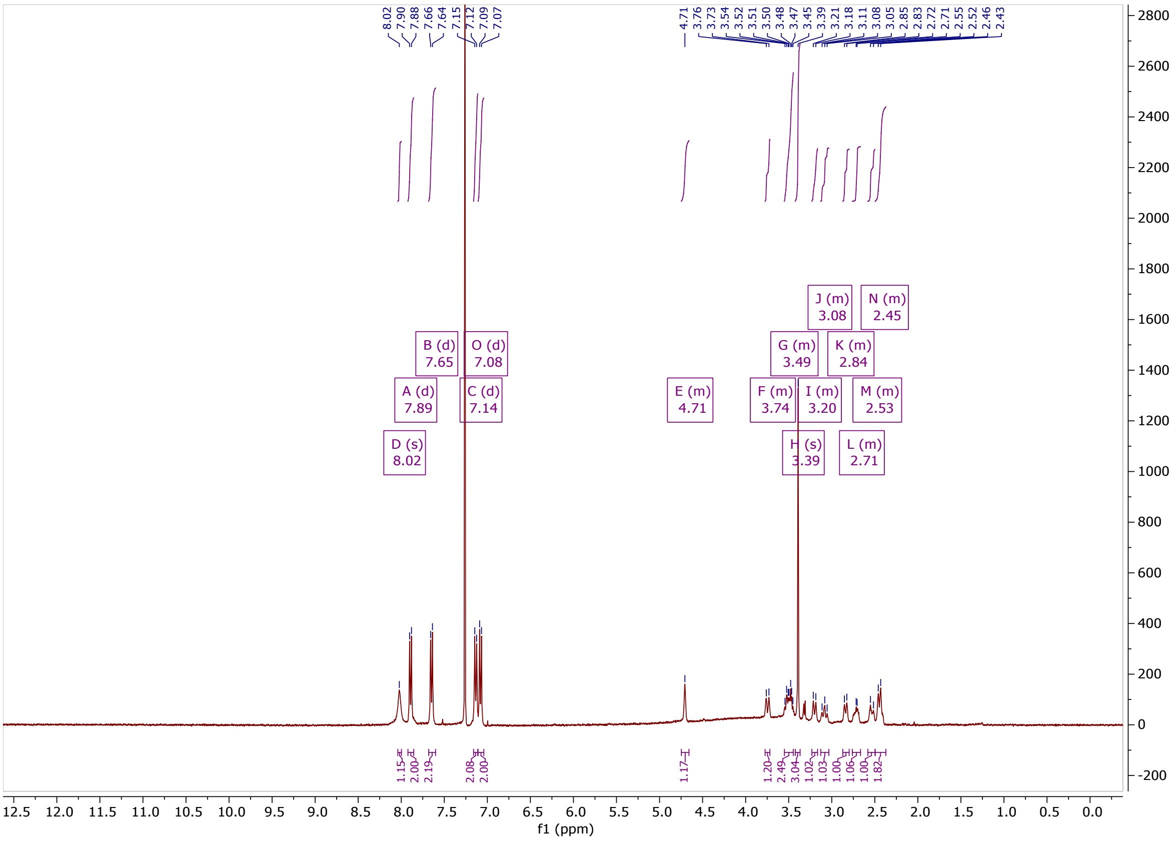

^{1}H NMR spectrum of **29**

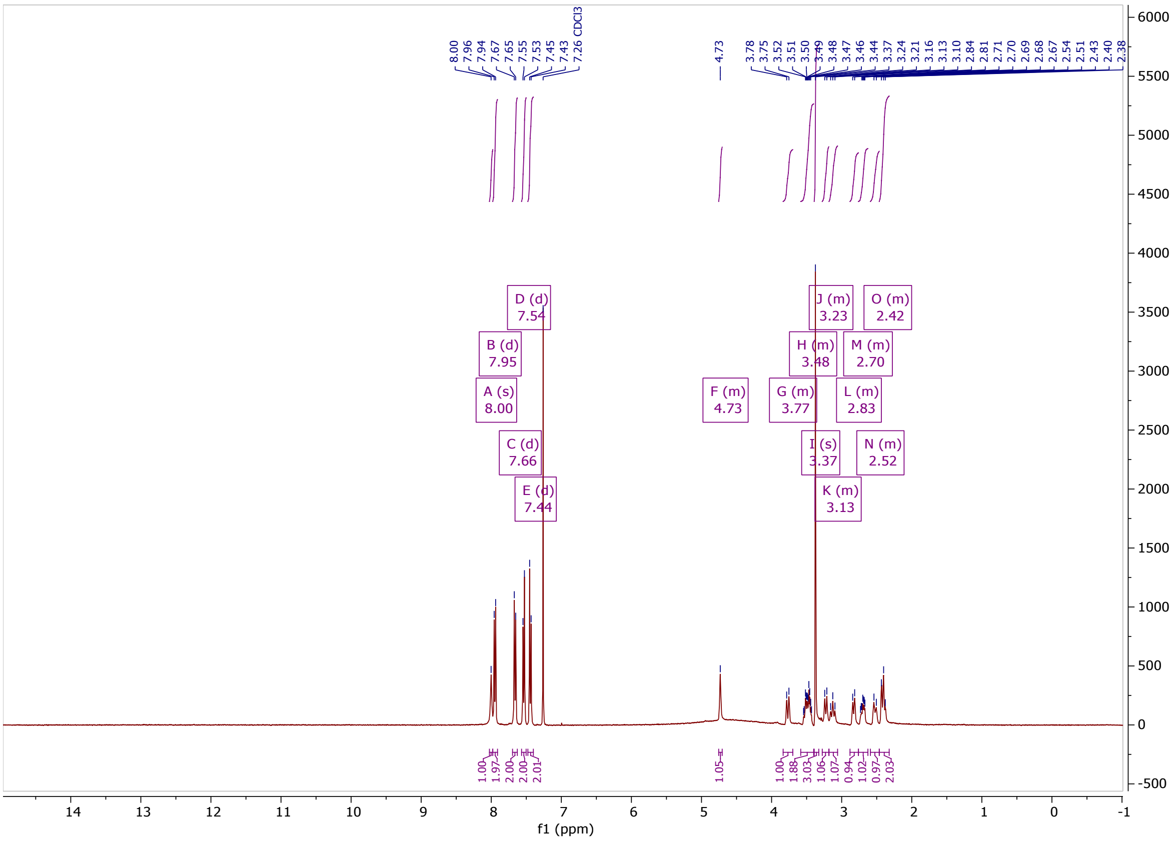

^{1}H NMR spectrum of **30**

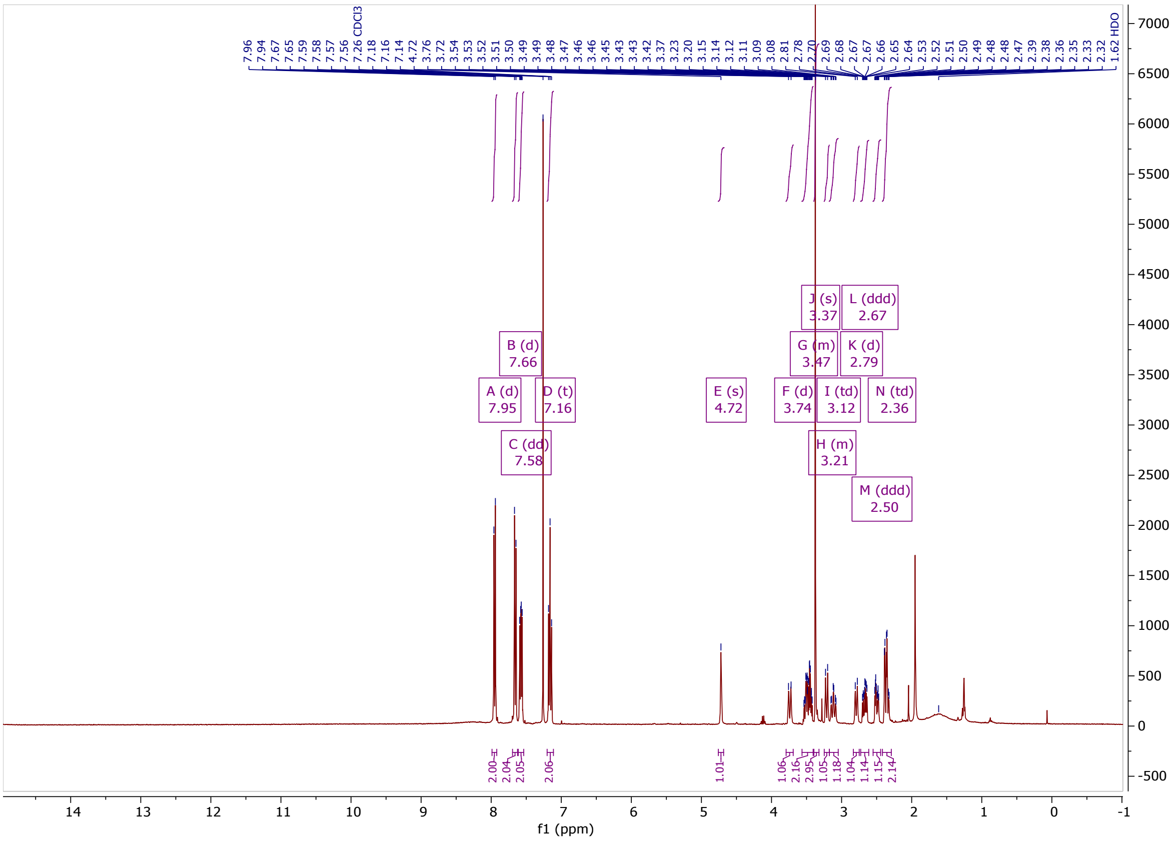

^{13}C NMR spectrum of **30**

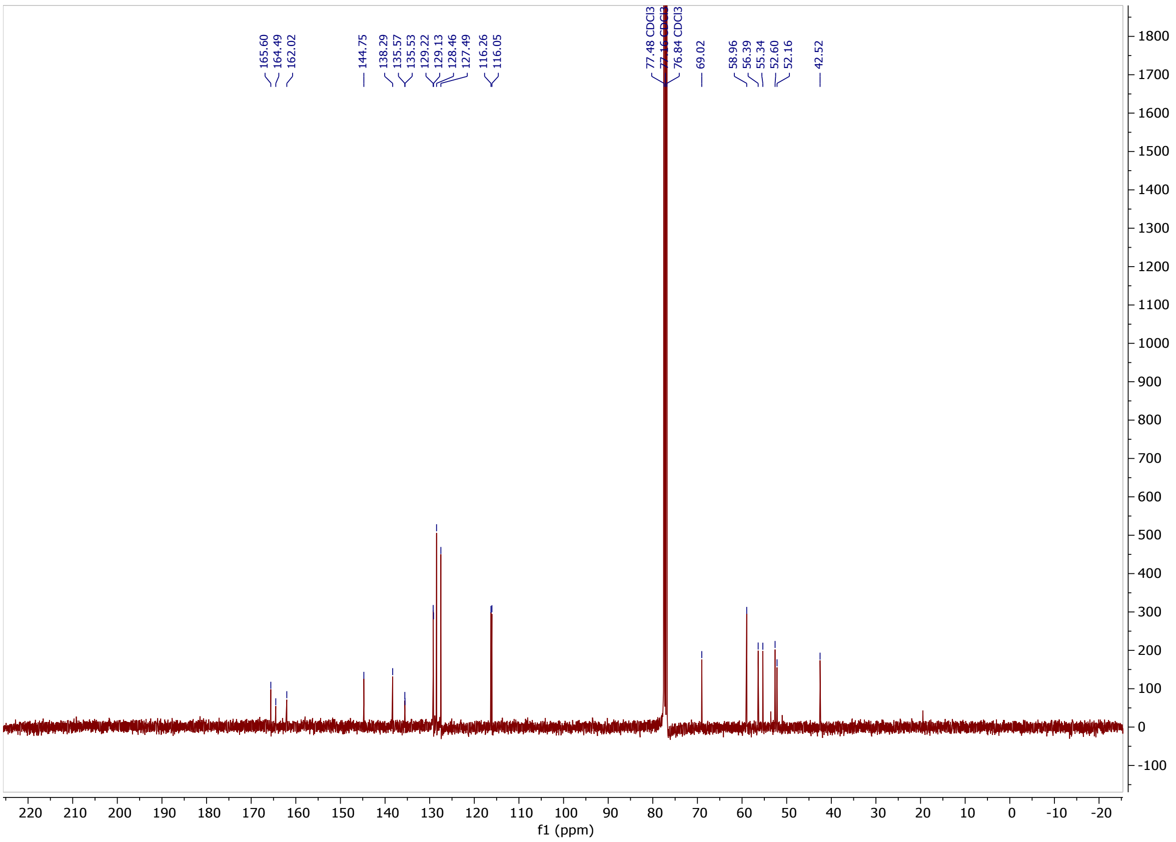

HPLC traces of the samples

15

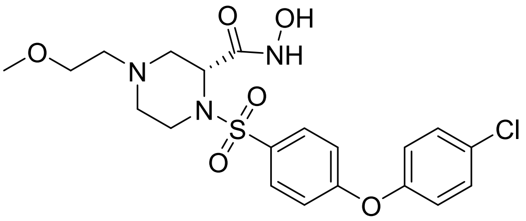

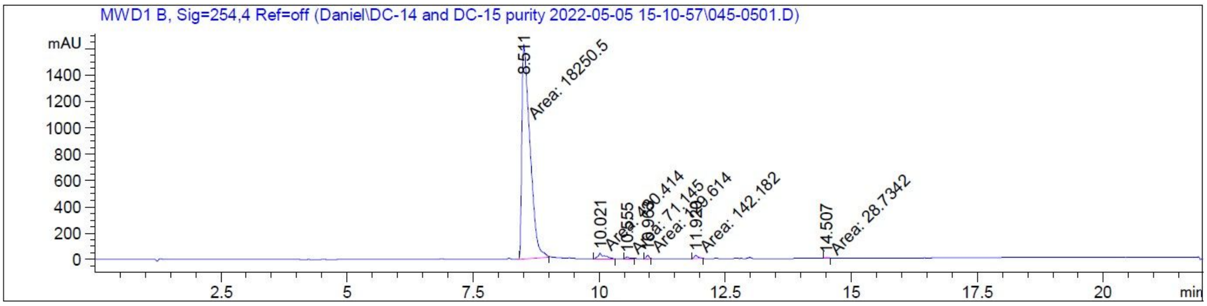

Signal 1: MWD1 B, Sig=254,4 Ref=off

Peak #	RetTime [min]	Type	Width [min]	Area [mAU*s]	Height [mAU]	Area %
1	8.511	MM	0.1867	1.82505e4	1629.58655	95.5394
2	10.021	MM	0.1743	480.41357	45.94951	2.5149
3	10.555	MM	0.0812	71.14499	14.60292	0.3724
4	10.963	MM	0.0753	129.61421	28.70428	0.6785
5	11.920	MM	0.0943	142.18156	25.12724	0.7443
6	14.507	MM	0.0841	28.73417	5.69592	0.1504
Totals :				1.91026e4	1749.66641	

16

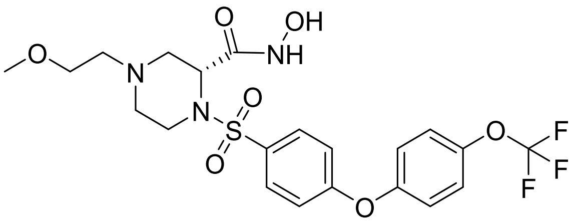

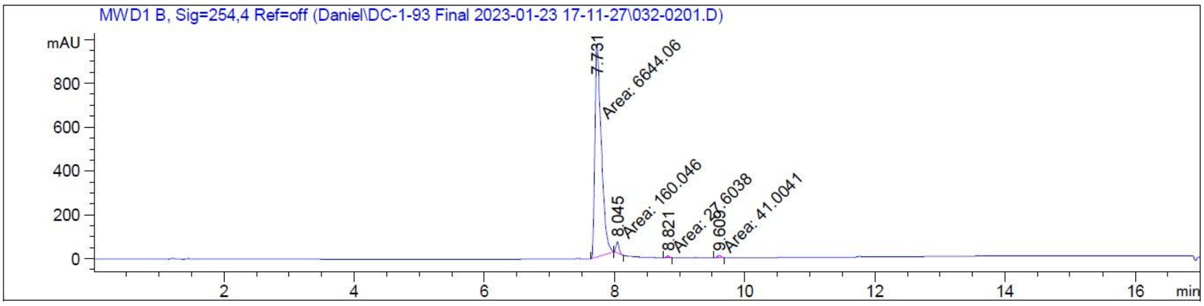

Signal 1: MWD1 B, Sig=254,4 Ref=off

Peak #	RetTime [min]	Type	Width [min]	Area [mAU*s]	Height [mAU]	Area %
1	7.731	MM	0.1140	6644.06104	971.28638	96.6730
2	8.045	MM	0.0511	160.04649	52.21545	2.3287
3	8.821	MM	0.0559	27.60382	8.23467	0.4016
4	9.609	MM	0.0582	41.00407	11.74232	0.5966
Totals :				6872.71542	1043.47882	

17

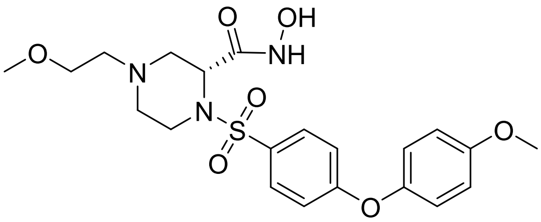

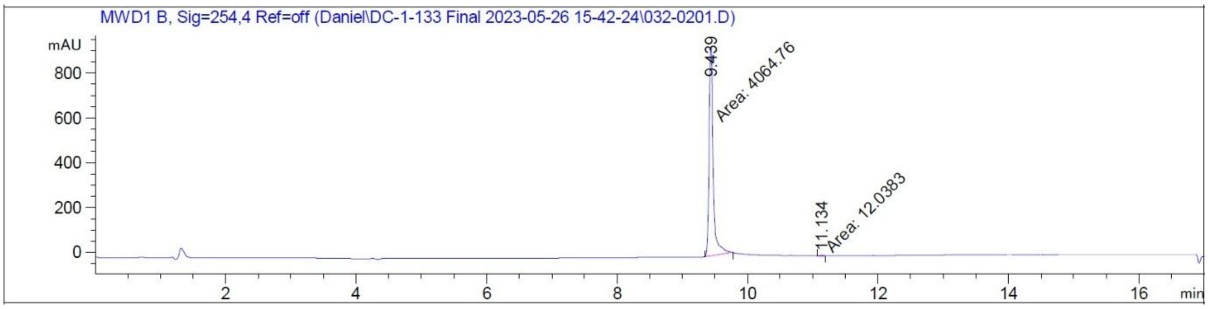

Signal 2: MWD1 B, Sig=254,4 Ref=off

Peak #	RetTime [min]	Type	Width [min]	Area [mAU*s]	Height [mAU]	Area %
1	9.439	MM	0.0716	4064.75952	946.13342	99.7047
2	11.134	MM	0.0807	12.03830	2.48760	0.2953
Totals :				4076.79782	948.62102	

18

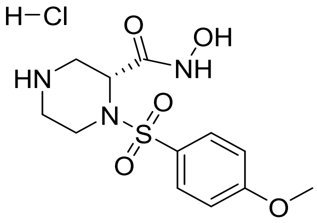

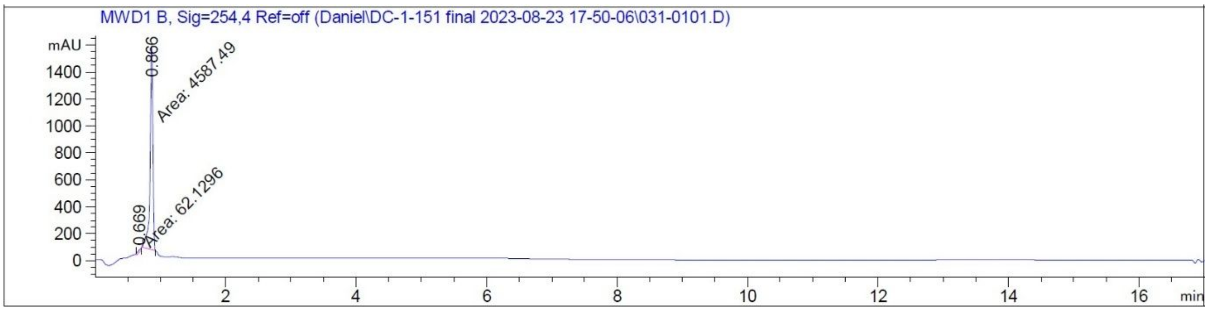


```
Signal 2: MWD1 B, Sig=254,4 Ref=off

Peak RetTime Type  Width     Area      Height     Area
  #   [min]         [min]   [mAU*s]     [mAU]       %
----|-------|----|-------|----------|----------|--------|
   1    0.669 MM    0.0649   62.12963   15.94834   1.3362
   2    0.866 MM    0.0502 4587.49072 1523.10852  98.6638

Totals :                   4649.62035 1539.05686
```

19

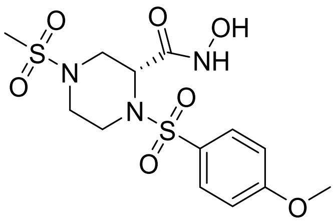

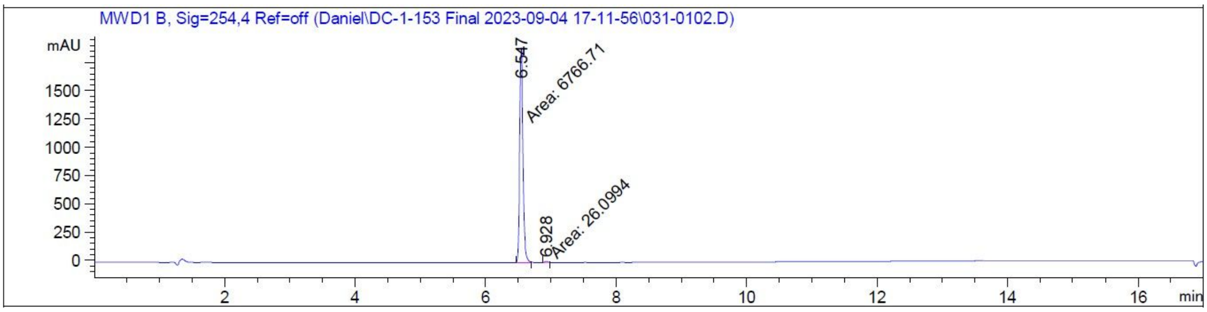

Signal 2: MWD1 B, Sig=254,4 Ref=off

Peak #	RetTime [min]	Type	Width [min]	Area [mAU*s]	Height [mAU]	Area %
1	6.547	MM	0.0590	6766.70605	1911.71704	99.6158
2	6.928	MM	0.0493	26.09939	8.82774	0.3842
Totals :				6792.80545	1920.54478	

20

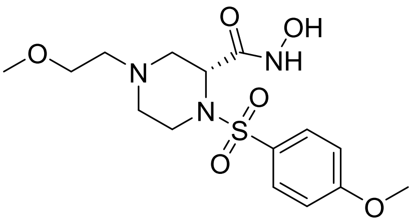

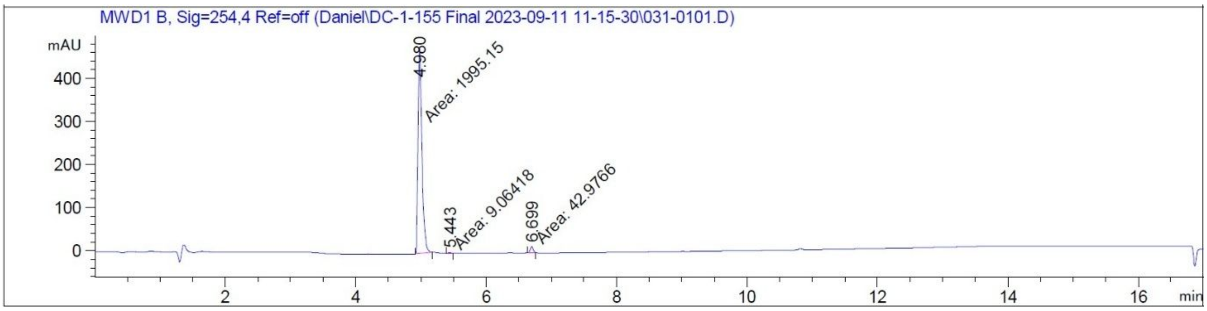

Signal 2: MWD1 B, Sig=254,4 Ref=off

Peak #	RetTime [min]	Type	Width [min]	Area [mAU*s]	Height [mAU]	Area %
1	4.980	MM	0.0689	1995.14575	482.60843	97.4579
2	5.443	MM	0.0607	9.06418	2.48880	0.4428
3	6.699	MM	0.0513	42.97656	13.96926	2.0993
Totals :				2047.18649	499.06649	

21

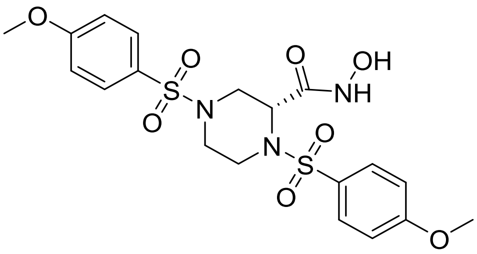

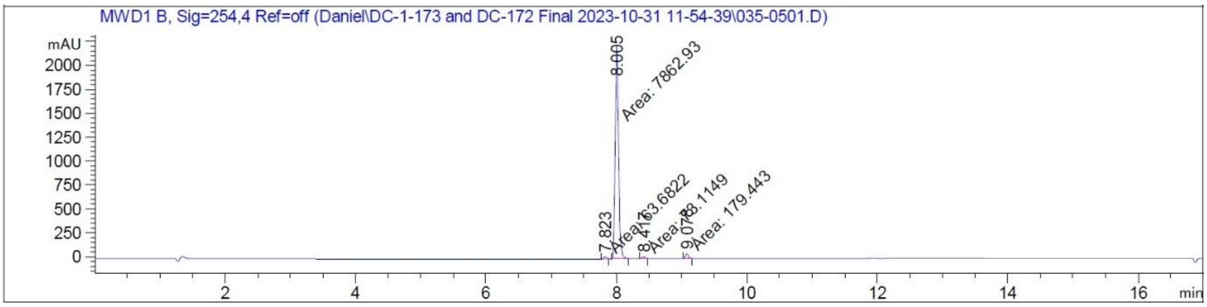

Signal 2: MWD1 B, Sig=254,4 Ref=off

Peak #	RetTime [min]	Type	Width [min]	Area [mAU*s]	Height [mAU]	Area %
1	7.823	MM	0.0510	63.68225	20.82471	0.7786
2	8.005	MM	0.0587	7862.92725	2234.38428	96.1336
3	8.417	MM	0.0535	73.11490	22.78942	0.8939
4	9.078	MM	0.0560	179.44345	53.45038	2.1939

Totals : 8179.16784 2331.44878

22

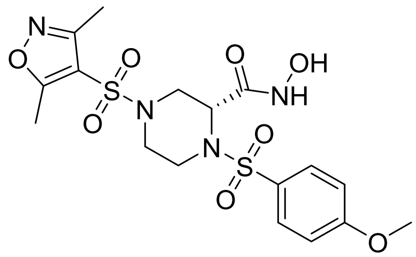

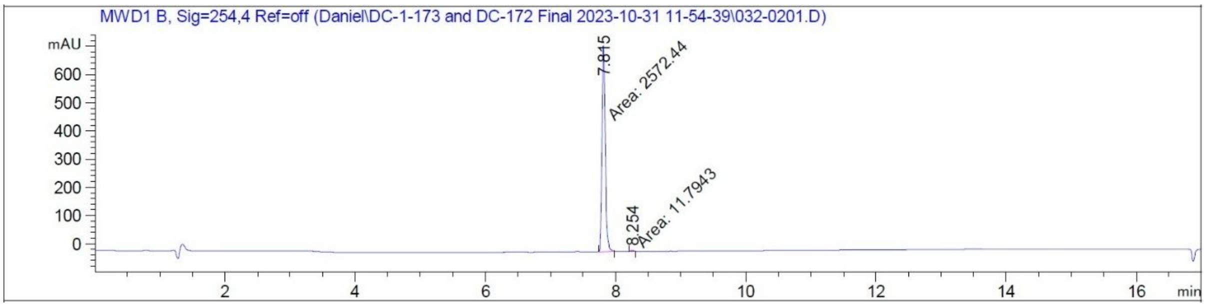

Signal 2: MWD1 B, Sig=254,4 Ref=off

Peak #	RetTime [min]	Type	Width [min]	Area [mAU*s]	Height [mAU]	Area %
1	7.815	MM	0.0580	2572.43970	739.35779	99.5436
2	8.254	MM	0.0534	11.79435	3.68048	0.4564

Totals : 2584.23404 743.03827

23 (DC-174)

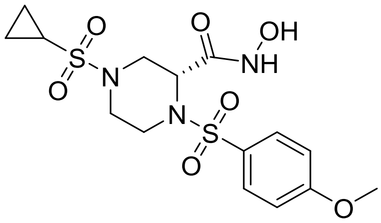

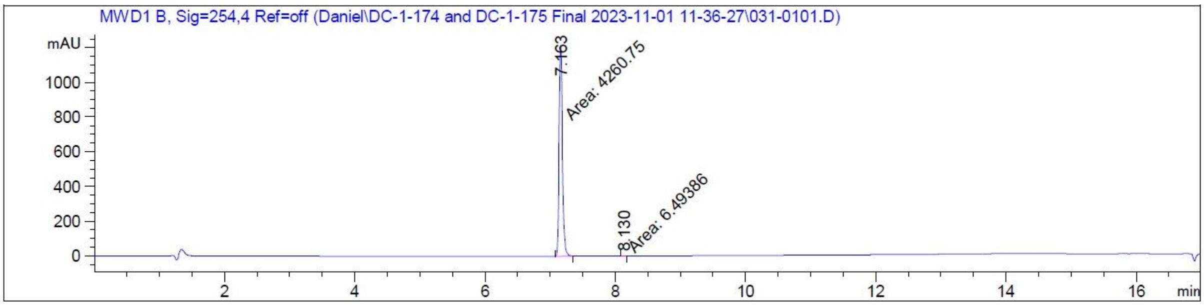

Signal 2: MWD1 B, Sig=254,4 Ref=off

Peak #	RetTime [min]	Type	Width [min]	Area [mAU*s]	Height [mAU]	Area %
1	7.163	MM	0.0581	4260.74561	1222.90796	99.8478
2	8.130	MM	0.0567	6.49386	1.90844	0.1522

28

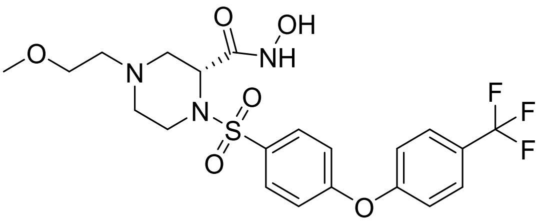

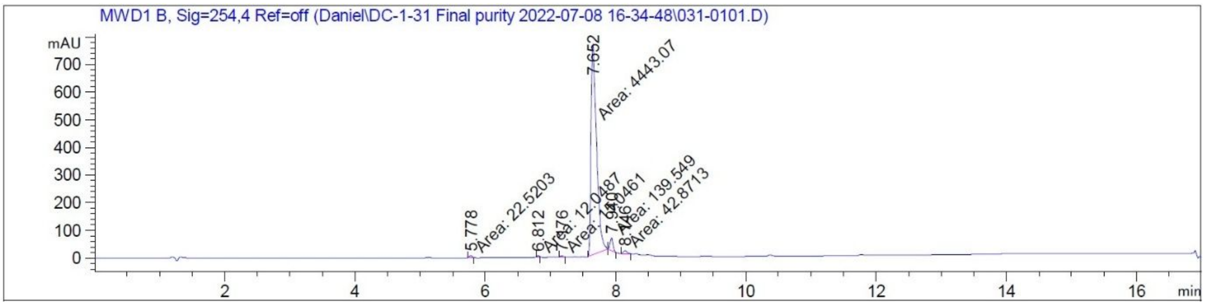

Signal 1: MWD1 B, Sig=254,4 Ref=off

Peak #	RetTime [min]	Type	Width [min]	Area [mAU*s]	Height [mAU]	Area %
1	5.778	MM	0.0507	22.52026	7.40401	0.4817
2	6.812	MM	0.0397	12.04874	5.05228	0.2577
3	7.176	MM	0.0525	15.04609	4.77448	0.3218
4	7.652	MM	0.0973	4443.07422	761.03986	95.0368
5	7.940	MM	0.0518	139.54942	44.92149	2.9849
6	8.146	MM	0.0676	42.87134	10.56538	0.9170

Totals : 4675.11006 833.75750

29

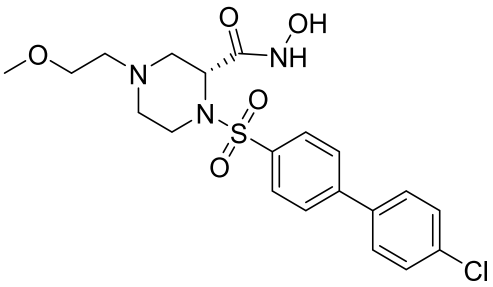

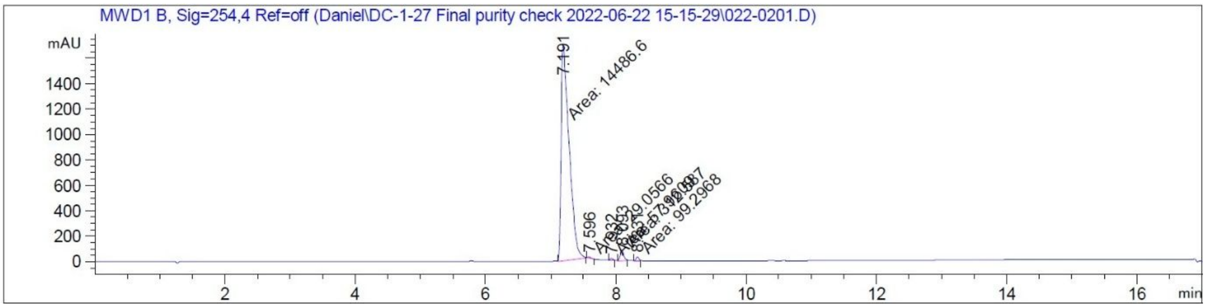

Signal 1: MWD1 B, Sig=254,4 Ref=off

Peak #	RetTime [min]	Type	Width [min]	Area [mAU*s]	Height [mAU]	Area %
1	7.191	MM	0.1419	1.44866e4	1701.34167	96.6708
2	7.596	MM	0.0497	29.05659	9.73809	0.1939
3	7.932	MM	0.0556	57.96090	17.38863	0.3868
4	8.093	MM	0.0645	312.58688	80.75212	2.0859
5	8.331	MM	0.0539	99.29678	30.70459	0.6626

Totals : 1.49855e4 1839.92510

30

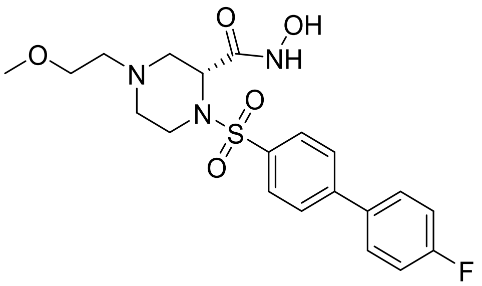

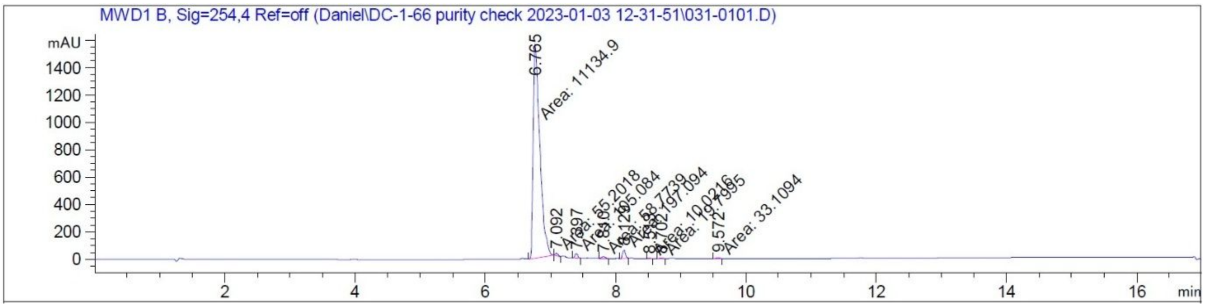


```
Signal 1: MWD1 B, Sig=254,4 Ref=off

Peak RetTime Type  Width     Area      Height     Area
  #   [min]        [min]   [mAU*s]     [mAU]       %
----|-------|----|-------|----------|----------|--------|
   1   6.765 MM    0.1189 1.11349e4  1560.84570  95.8749
   2   7.092 MM    0.0500   55.20181   18.38698   0.4753
   3   7.397 MM    0.0539  105.08374   32.47163   0.9048
   4   7.810 MM    0.0691   58.77395   14.17370   0.5061
   5   8.129 MM    0.0522  197.09447   62.89034   1.6970
   6   8.513 MM    0.0496   10.02161    3.36678   0.0863
   7   8.702 MM    0.0648   19.79950    5.08985   0.1705
   8   9.572 MM    0.0604   33.10936    9.12894   0.2851

Totals :                  1.16140e4  1706.35393
```

Supplementary Figures

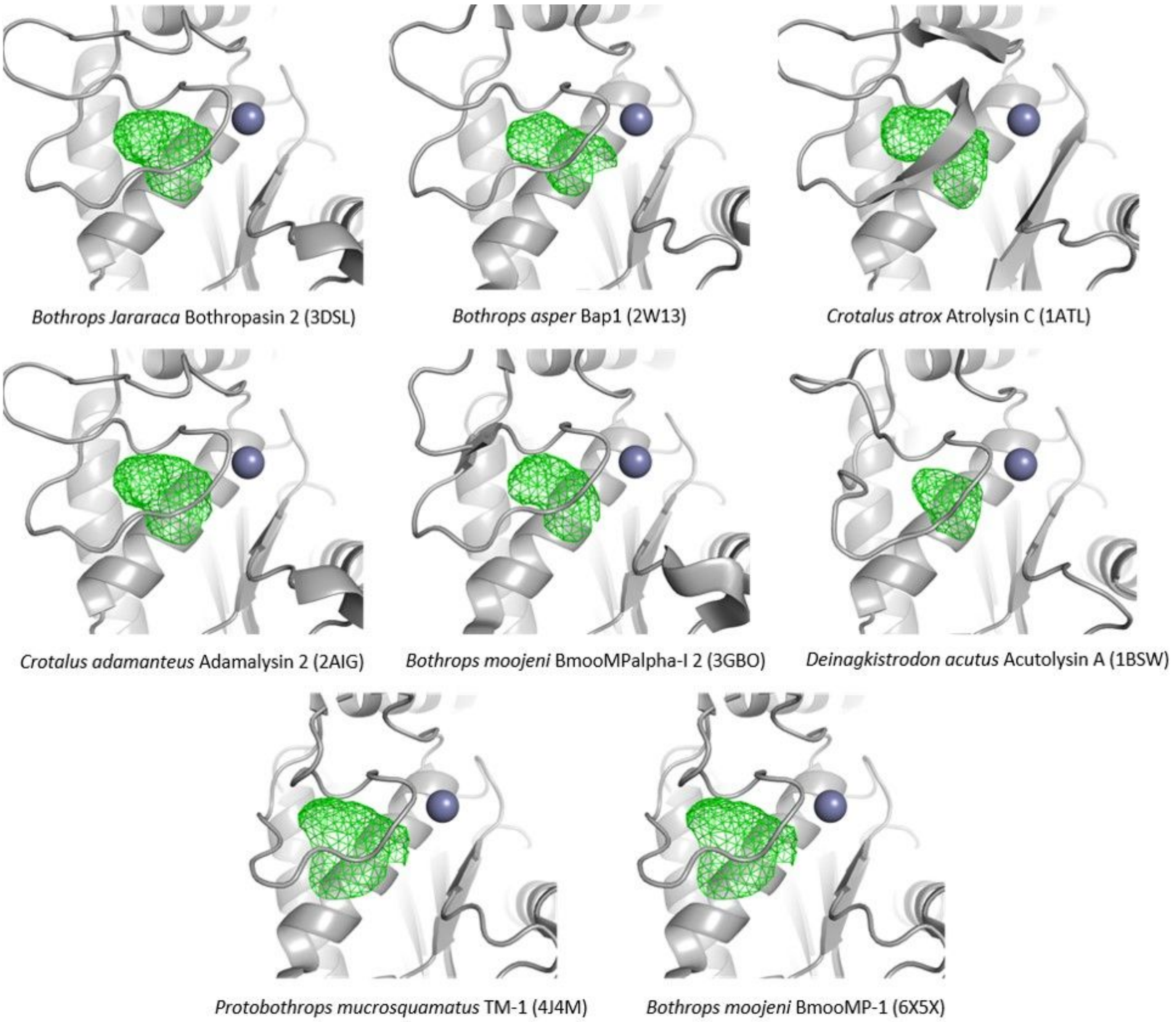

Figure S1. Structures of SVMPs from different species of snake available in the PDB database. Accession codes are given in parentheses. Protein secondary structure is represented by grey cartoon, the S1' pocket volume is shown as green wireframe and the catalytic zinc ion is shown as a blue sphere.

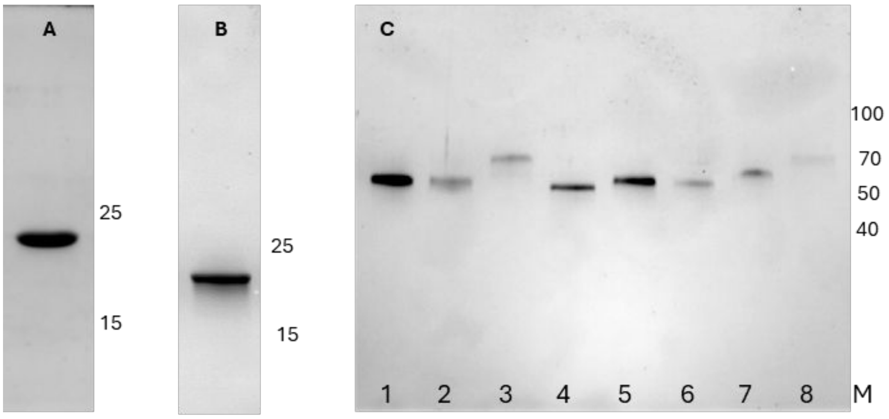

Figure S2. SDS-PAGE analysis of the purified *E. romani* SVMPs. Gel **A**: 22 kDa P-I SVMP. The gel used was 15% acrylamide, made in-house. Gel **B**: 21 kDa P-II SVMP. The gel used was 4-20% acrylamide (BioRad). Gel **C**: P-III SVMPs. The gel used was BioRad 'Any kD'. Lane 1-8, the eight main forms of 52-68 kDa P-III SVMPs individually isolated. When run under non-reducing conditions, the P-III SVMPs in lanes 4 and 5 were shown to be P-IIIc (dimer) forms, the rest were P-IIIa (monomers). All eight were pooled to provide a P-III SVMP sample for use in this study. All gels were stained with Coomassie Blue R250 and key molecular weight markers (Thermo Page Ruler) are indicated in kDa on the right.

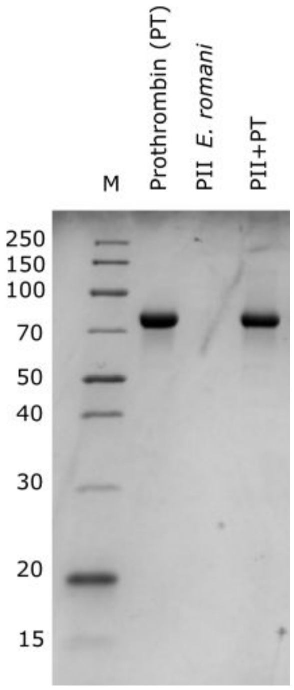

Figure S3. The P-II SVMP from *E. romani* venom does not degrade prothrombin. Prothrombin degradation gel in the presence of P-II SVMP isolated from *E. romani*. Prothrombin (2 µg), SVMP II (200 ng) or prothrombin pre-incubated with 200 ng SVMP II for 1h at 37 ºC (lane 3) were run on an SDS-PAGE gel and stained with Coomassie Brilliant Blue. Protein molecular weights of the marker (M) in kDa are shown on the left.

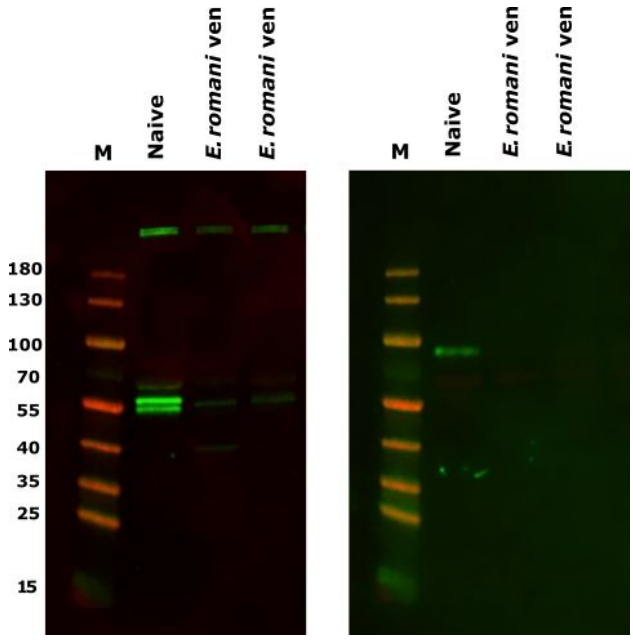

Figure S4. ***E. romani*** **venom depletes fibrinogen and prothrombin** ***in vivo*****.** Anti-fibrinogen (left) and anti-prothrombin (right) western blots of plasma from mice injected intraperitoneally with 90 µg of *E. romani* (n=2) versus naïve controls. Whereas the three chains of murine fibrinogen (alpha, beta and gamma, ~55-65 kDa) are present for the naïve unenvenomed control, a single faint beta chain band remains following the administration of *E. romani* venom (left). Complete degradation of murine prothrombin is seen in envenomed mice (right). M – prestained molecular weight marker.

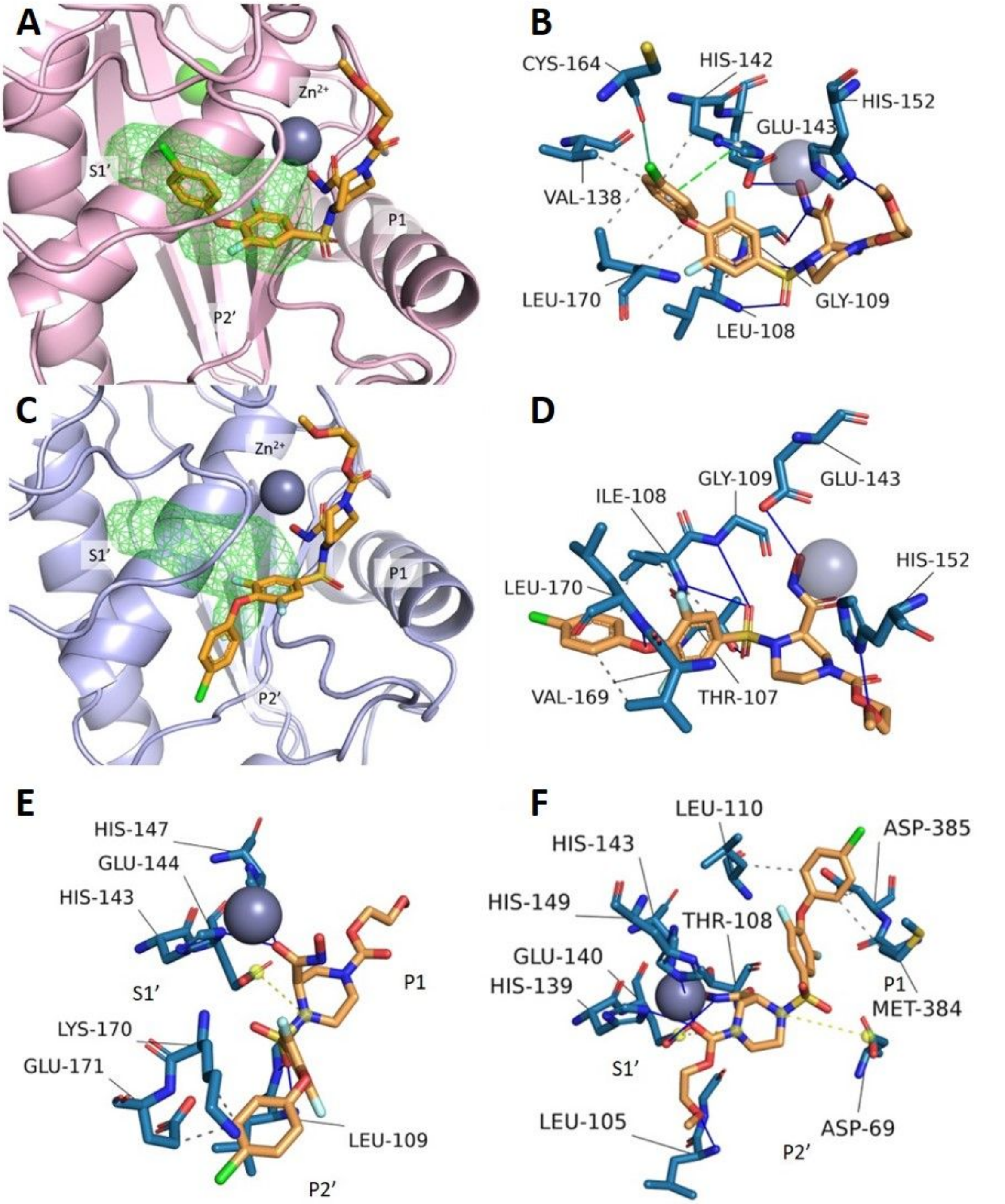

Figure S5. Molecular modelling predicted binding poses and interactions between SVMPs and 1f (XL-784). Docked poses of XL-784 (orange sticks) in atrolysin C SVMP (pink cartoons, PDB: 1ATL) (**A** and **B**) and BAP-1 SVMP (light blue cartoons, PDB: 2W13) (**C** and **D**), ecarin (PDB: 9CLP) (**E**) and RVV-X (PDB: 2E3X) (**F**). (A and C) Structure of the docked inhibitors in the SVMP binding site, the S1' pocket is highlighted in green wireframe. Zn^{2+} and Ca^{2+} ions are shown in deep blue and green, respectively. (B, D, E, F) Analysis of non-covalent interactions formed by inhibitors in the docked structures. Binding residues are shown in blue, hydrophobic interactions are shown as grey dashed lines, salt bridges are shown in yellow dashed lines, π-stacking interaction are shown as green dashed lines, halogen bonds are shown as green solid lines, and hydrogen bonding interactions are shown as blue solid lines.

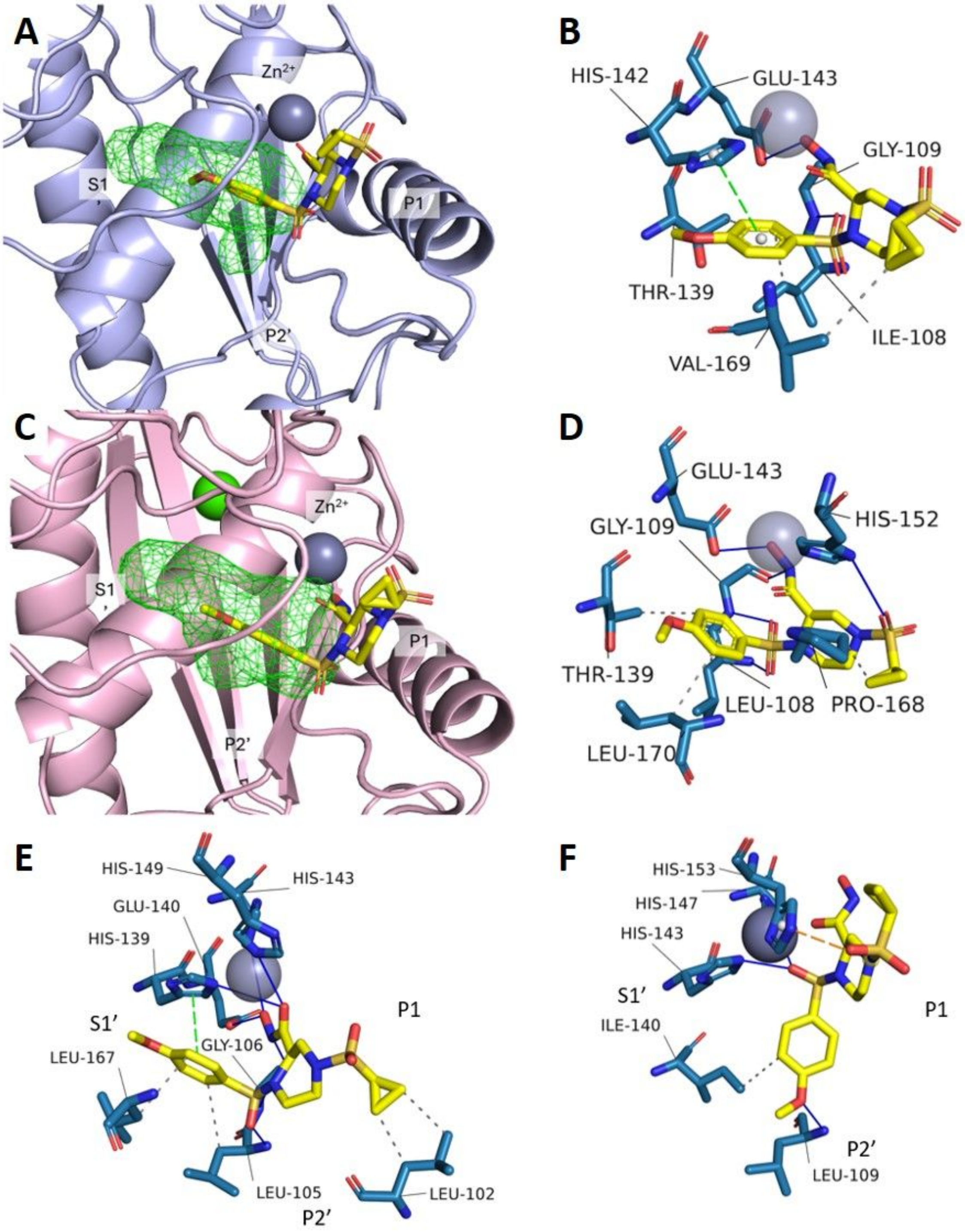

Figure S6. Molecular modelling predicted binding poses and interactions between SVMPs and 23 (DC-174). Docked poses of **23** (yellow sticks) in BAP-1 SVMP (light blue cartoons, PDB: 2W13) and atrolysin C SVMP (pink cartoons, PDB: 1ATL) (**A** and **C**) - Structure of the docked inhibitors in the SVMP binding site, the S1' pocket is highlighted in green wireframe. Zn^{2+} and Ca^{2+} ions are shown in deep blue and green, respectively. (**B** and **D**) Analysis of non-covalent interactions formed by inhibitors in the docked structures. Binding residues are shown in blue, hydrophobic interactions are shown as grey dashed lines, π-stacking interaction are shown as green dashed lines, and hydrogen bonding interactions are shown as blue solid lines.

Molecular Modelling and Docking. To investigate binding of **1f** (XL-784) and **23** (DC-174) in representative SVMPs, we carried out molecular docking simulations. Compounds were built and energy minimised at the MMFF level using Spartan24 [Spartan '24 version 1.1.0 (Wavefunction, 2024)]. Energy minimised compounds were docked into available crystal structures of relevant SVMP from venoms used in *in vitro* profiling; *Bothrops jararaca* (bothropasin, PDB: 3DSL)[1], *Crotalus atrox* (atrolysin C, PDB: 1ATL)[2], *Bothrops asper* (BaP-1, PDB: 2W13)[3], *Echis carinatus* (ecarin, PDB: 9CLP)[4] and *Daboia siamensis* (RVV-X, PDB: 2E3X)[5]. Docking was carried out in GOLD[6] with a docking protocol validated by re-docking of the co-crystallised ligands for each SVMP. Docking was carried out without allowed early termination and allowed flipping of amides, pyramidal nitrogen and flipping ring corners, default settings were used otherwise. Harmonic distance restraints were applied to atoms involved in chelation of the catalytic zinc in the SVMP catalytic site with minimum distance set at 1.5 Å, maximum distance set at 2.5 Å and spring constant set at 10 – in each extracted ligand the bound moieties are carboxylate groups interacting with zinc by each oxygen. The CHEMPLP scoring function to generate poses. This afforded docking poses with RMSD values of < 1.5 Å in each structure with respect to the extracted ligand.

1f (XL-784) and **23** (DC-174) were docked into each SVMP using the validated protocol. Harmonic restraints were applied between the hydroxamic acid carbonyl and sp^3 oxygen, and Zn with the same distance thresholds and spring constant as described above. Top ranked poses by CHEMPLP score in each SVMP were extracted and analysed for non-covalent interactions using PLIP[7].

Molecular dynamics

To sample the flexibility of the P1 pocket, we used each homology model as starting coordinates for molecular dynamics simulations. For each SVMP model we carried out five replica 5 ns simulations, generating new velocities for each replicate run. The molecular dynamics (MD) simulations were performed using Gromacs 2022.0 and the Amber FF99-SB ILDN force field[8]. Following the preparation of the system, steepest descent energy minimization was conducted for 50,000 steps. The system was subsequently equilibrated for 100 ns in the NVT followed by 100 ns simulation in the NPT ensemble with positional restraints on protein heavy atoms. The production MD was performed with a leap-frog integrator using a time step of 2 fs for a total simulation duration of 5 ns. Long-range electrostatics were computed using the Particle Mesh Ewald (PME) method, with a real-space cut off of 1.2 nm, and van der Waals interactions were treated with a force-switch modifier within a 1.0–1.2 nm cut off. LINCS constraints were applied to all bonds involving hydrogen. Temperature was controlled using a modified Berendsen thermostat (V-rescale) at 300 K[9], with separate coupling groups for the protein and solvent, and pressure was maintained at 1 bar using the Parrinello-Rahman barostat[10]. Output coordinates, energies, and logs were saved every 10 ps.

Mdtraj was used to process trajectories[11]. Trajectories were pre-processed by discarding the first nanosecond of each for equilibration – totalling 20 ns simulation time for each subtype. Trajectories were concatenated and clustered into 12 clusters each using agglomerative clustering as implemented in scikit-learn[12] based on the root-mean-square deviation of protein heavy atoms within 12.0 Å of the catalytic zinc ion. Cluster centroids were extracted for use in

ensemble docking. In simulations involving P-I, P-III_1, P-III_2 and P-III_3 the catalytic zinc was observed to dissociate – cluster centroids where the zinc had dissociated were not included for ensemble docking. Structures of cluster centroids were aligned to each other based on protein heavy atoms within 12.0 Å of the catalytic zinc using the Pymol python API[13].

Supplementary Computational Studies

Additional discussion for molecular docking studies

Docking studies using molecular dynamics-derived ensembles of *E. romani* SVMP homology models (one P-I, one P-II, and three P-III; see Figure S7 sequence selections for homology modelling; see Table S1 for of protein sequences used for homology model building; Table S2 for summary of ensemble docking) were performed to assess binding of DC-174, prinomastat, and **1f** (XL-784). Both **23** (DC-174), and **1d** (prinomastat) formed binding modes across all SVMP subtypes (see Figure S8). However, XL-784 failed to bind effectively to P-II SVMPs, likely due to its bulky biaryl side chain not fitting in the P1' pocket. These findings align with fibrinogen cleavage data (Figure 3A) and are further detailed in the discussion below.

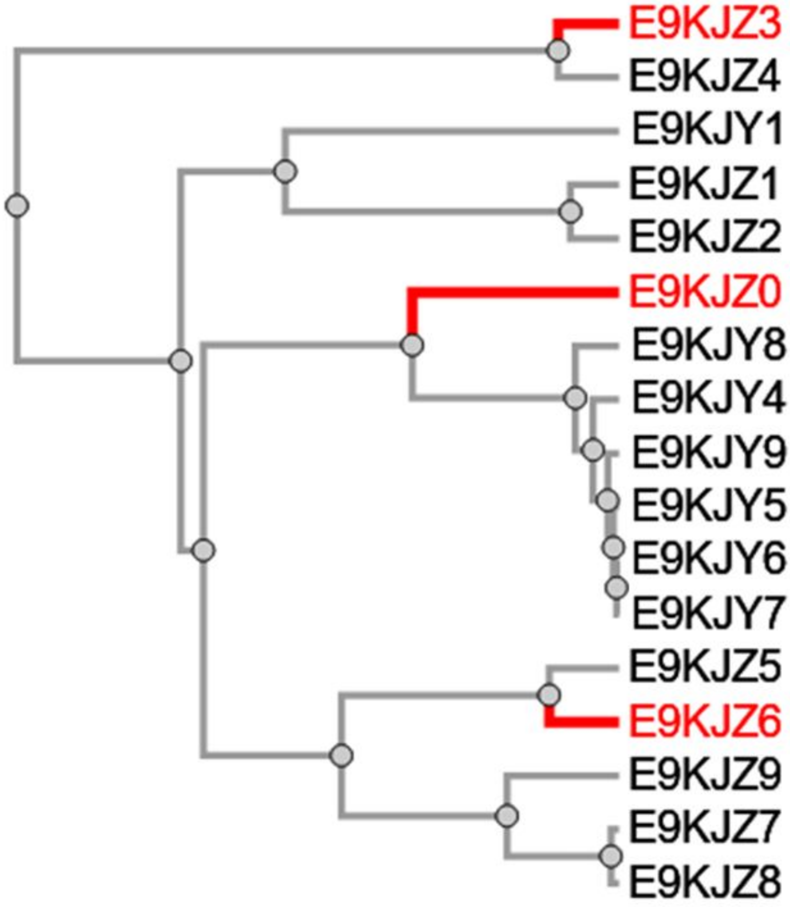

Figure S7. Phylogenetic tree of P-III sequences denoted by their UniProt identifier. Identifiers highlighted in red denote the P-III sequences used to build homology models for this work. Primary sequence data for *E. romani* snake venom metalloproteinases (SVMPs) were obtained from UniProt (EoMP06, sequence ID Q6X1T6), comprising two P-I, seven P-II, and 17 P-III SVMP sequences; one representative sequence each from the P-I and P-II subclasses was selected based on sequence coverage, while three P-III sequences were chosen using a phylogenetic analysis conducted with the EMBL-EBI phylogenetics tool in Clustal Omega[14]. Homology models were generated using the SWISS-MODEL web API, model templates were automatically assigned based on sequence similarity[15]. We selected the highest scoring model with the catalytic zinc ion present. Model quality metrics, including Global Model Quality Estimation (GMQE), QMEANDisCo global score, and template coverage, were evaluated, and all selected models passed reliability criteria detailed in table S2, which also summarizes UniProt identifiers and corresponding template structures. All models produced had a QMEANDisCo score of > 0.6 indicating reasonable model quality[16].

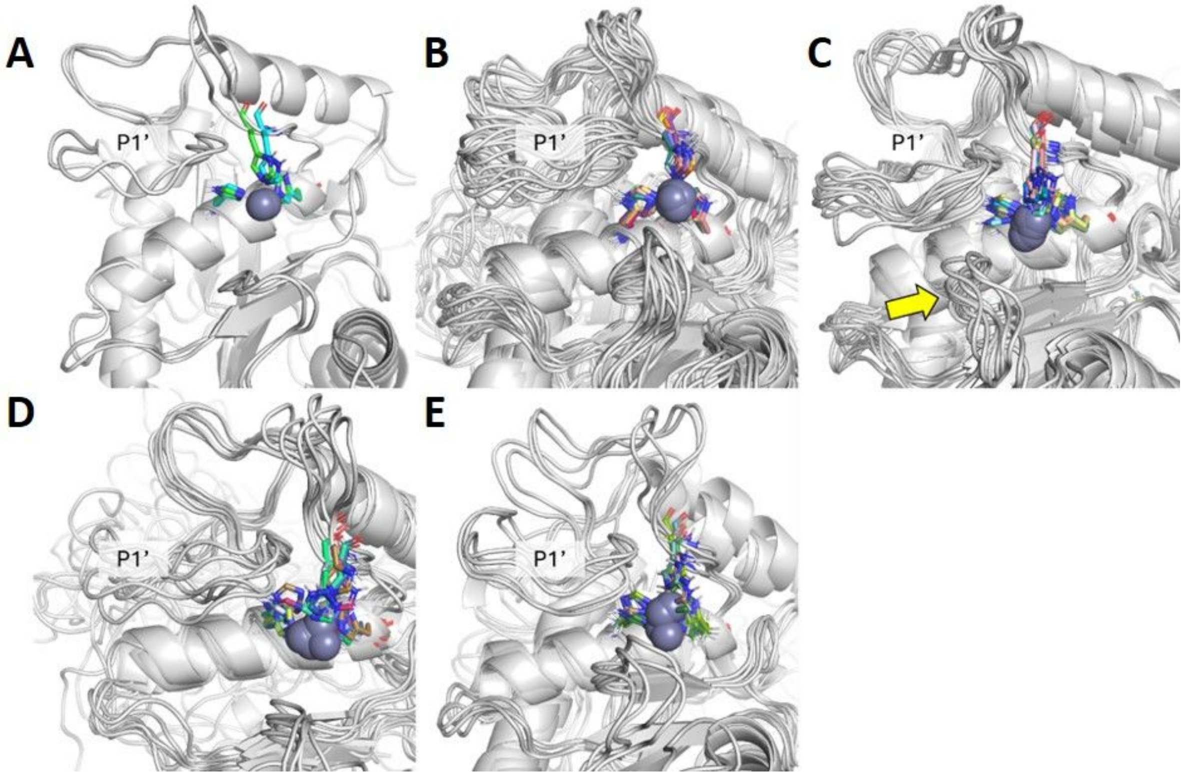

Figure S8. Conformational ensembles extracted from MD simulations of *E. romani* SVMP subtype homology models; (**A**) P-I, (**B**) P-II, (**C**) P-III_1, (**D**) P-III_2, (**E**) P-III_3. The P1' pocket is annotated in each panel. In panel c, an adjacent loop which was observed to occlude the P1' pocket is highlighted by the yellow arrow. Ensemble docking was carried out using the CCDC GOLD Python API[17]. Docking was carried out as described as in the main text with modifications to automate setting of harmonic distance constraints using the Gold Python API. The top ten binding poses as determined by ChemPLP scoring function were visually analysed for presence of the ideal binding mode. All scripts used in this work and Pymol sessions of docked structures are available at https://github.com/cmwoodley/SVMP_ensemble_docking.

Table S1. Summary of protein sequences used for homology model building.

Model	Code	Uniprot ID	Sequence Description [a]	Template [b]	Template Description	GMQE [c]	QMEANDisCo [d]
1	PI	E9KJX3	Group I snake venom metalloproteinase	2DW2	VAP2 from *Crotalus atrox* venom	0.43	0.68
2	PII	E9KJX0	Group II snake venom metalloproteinase	2DW2	VAP2 from *Crotalus atrox* venom	0.45	0.68
3	PIII_1	E9KJZ0	Group III snake venom metalloproteinase	2DW2	VAP2 from *Crotalus atrox* venom	0.62	0.74
4	PIII_2	E9KJZ3	Group III snake venom metalloproteinase	2DW0	VAP2 from *Crotalus atrox* venom	0.79	0.72
5	PIII_3	E9KJZ6	Group III snake venom metalloproteinase	2DW0	VAP2 from *Crotalus atrox* venom	0.62	0.74

[a] All sequences are from *E. romani* [b] PDB accession codes of templates used to build the homology model [c] GMQE – Global Model Quality estimation [d] QMEANDisCo – Quantitative Model Energy Analysis with Distance Constraints

Table S2. Summary of ensemble docking into *E. romani* SVMP subtypes.

		**23**				Prinomastat				XL-784			
Code	Interaction	Top 1[a]	Top 3[b]	Top 10[c]	Ideal[d]	Top 1[a]	Top 3[b]	Top 10[c]	Ideal[d]	Top 1[a]	Top 3[b]	Top 10[c]	Ideal[d]
P-I	Binds P1'	Y	3/3	10/10	5/10	Y	3/3	10/10	7/10	Y	3/3	9/10	4/10
	Sulfone HB	N	1/3	5/10		Y	3/3	7/10		N	1/3	5/10	
P-II	Binds P1'	N	1/3	4/10	3/10	Y	1/3	5/10	2/10	N	0/3	0/10	0/10
	Sulfone HB	Y	2/3	5/10		N	0/3	2/10		N	1/3	1/10	
P-III _1	Binds P1'	N	0/3	0/10	0/10	Y	0/3	0/10	0/10	N	0/3	0/10	0/10
	Sulfone HB	N	0/3	0/10		N	0/3	0/10		N	0/3	0/10	
P-III _2	Binds P1'	N	0/3	2/10	1/10	N	0/3	2/10	1/10	N	0/3	0/10	0/10
	Sulfone HB	N	0/3	2/10		N	0/3	1/10		N	0/3	1/10	
P-III _3	Binds P1'	Y	3/3	9/10	5/10	Y	3/3	10/10	1/10	Y	1/3	5/10	4/10
	Sulfone HB	Y	2/3	5/10		N	1/3	1/10		Y	1/3	4/10	
	**Total P1':**	2	7	25		4	7	27		2	4	14	
	**Total Sulfone:**	2	5	17		1	4	11		1	3	11	
	**Totals:**	4	12	42	14	5	11	38	11	3	7	25	8

[a] Presence of a given interaction in the top scoring pose [b] Presence of a given interaction in the top 3 poses [c] Presence of a given interaction in the top 10 poses [d] Presence of the ideal binding mode, defined by a pose in which the aryl substituent is bound within the P1' pocket and the sulfonamide forms hydrogen binding interactions with the protein backbone.

Molecular modelling of E. romani SVMP isoforms

A limitation of docking into available X-ray structures of SVMPs is that it does not account for the diversity of SVMP isoforms within a species. Our gel electrophoresis experiments demonstrated that different molecules exhibited distinct inhibition profiles against various SVMP subtypes from *E. romani*. To address this, we docked **23** (DC-174), **1d** (prinomastat), and **1f** (XL-784) into homology models constructed from the primary sequences of *E. romani* P-I, P-II, and P-III SVMP subtypes (see Table S1 for protein sequences used for homology model building). The P1' pocket is known to be flexible[2, 3], so to sample conformational space, we performed multiple short unbiased molecular dynamics (MD) simulations using the homology models as starting coordinates. Representative structures were then extracted through clustering of the resulting trajectories; these ensembles are shown in Figure S8. In the first P-III isoform (Figure S8C) an adjacent disorganised loop occluded the P1' pocket in each representative structure; in the docking studies this prevented formation of interactions with the P1' pocket and backbone hydrogen bonds with the sulfonamide.

We used the observed binding modes to qualitatively compare ligands based on the number of poses in an ideal binding mode - defined as the aryl group occupying the P1' pocket and the sulfonamide forming hydrogen bonds with the protein backbone. The main limitation of this workflow is that we do not consider the free energy landscape of P1' pocket opening. This limits the inference of this method to assessing whether or not a ligand can form an ideal binding mode, rather than assessing how likely a ligand is to bind. However, due to the biological complexity of SVMPs within a given snake species venom, we believe this is a reasonable compromise considering conformational flexibility for multiple isoforms while balancing computational expense. A summary of these qualitative results of this ensemble docking is provided in Table S2.

Consistent with docking results using X-ray structures, **23** (DC-174) formed ideal binding modes across all *E. romani* SVMP isoforms, with the largest number of ideal binding poses compared to **1d** (Prinomastat) and **1f** (XL-784). Prinomastat also formed ideal binding modes with all isoforms, while XL-784 failed to produce any ideal binding poses with P-II SVMPs. This aligns with the poor inhibition of fibrinogen cleavage by XL-784 observed in gel electrophoresis assays (Figure 3A), where both DC-174 and Prinomastat showed significant inhibition. In our homology models, we observed the presence of an arginine residue in the P1' pocket of P-II SVMP, which may act as a gatekeeper, similar to the arginine in MMP1 that plays a crucial role in the selective inhibition of MMP13[18, 19]. This arginine residue likely prevents the bulky side chain of XL-784 from effectively binding within the P1' pocket of P-II SVMP.

Further analysis of the number of poses with the aryl group bound in the P1' pocket revealed comparable results for DC-174 (**23**) and Prinomastat (**1d**), whereas XL-784 (**1f**) showed fewer poses. This suggests that the bulkiness of the biaryl side chain in XL-784 hinders its ability to bind effectively in the P1' pocket, consistent with secondary assay observations. These studies highlight that truncating the aryl substituent to a single ring may be a viable strategy to achieve broad-spectrum activity both within and across species.

X-ray Crystallography data of 23 (DC-174)

Table S3. The determination of absolute configuration of **23** (DC-174) using X-ray crystallography analysis (CCDC deposition number: 2451930).

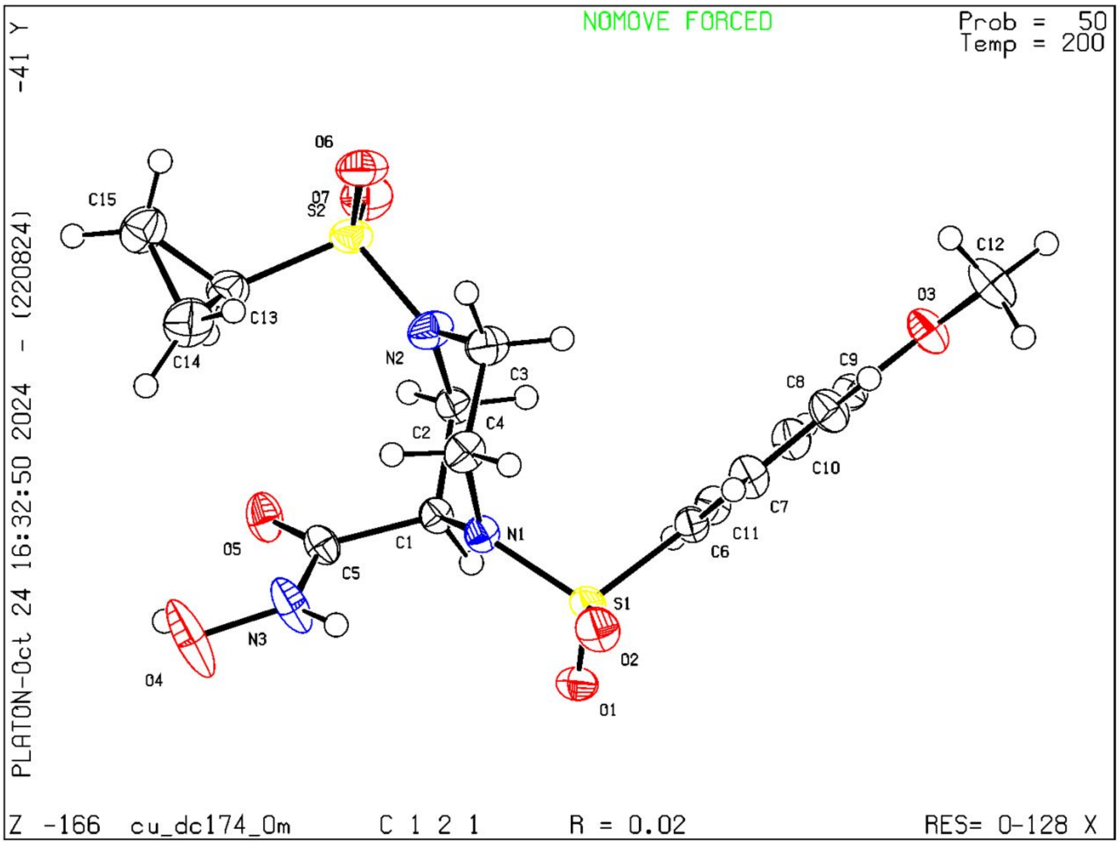

Crystal data and structure refinement for **23** (DC-174).

Identification code	cu_DC174_0m
Empirical formula	$C_{15}H_{21}N_3O_7S_2$
Formula weight	419.47
Temperature/K	200.0
Crystal system	monoclinic
Space group	C2
a/Å	19.7656(5)
b/Å	7.4345(2)
c/Å	14.2819(3)
α/°	90
β/°	118.6210(10)
γ/°	90
Volume/Å^3	1842.24(8)
Z	4
ρ_{calc}g/cm^3	1.512
μ/mm^{-1}	3.029
F(000)	880.0

Crystal size/mm^3	0.45 × 0.225 × 0.07
Radiation	CuKα (λ = 1.54178)
2Θ range for data collection/°	7.05 to 149.34
Index ranges	-24 ≤ h ≤ 24, -8 ≤ k ≤ 9, -17 ≤ l ≤ 17
Reflections collected	20717
Independent reflections	3595 [R_{int} = 0.0284, R_{sigma} = 0.0272]
Data/restraints/parameters	3595/1/249
Goodness-of-fit on F^2	1.092
Final R indexes [I>=2σ (I)]	R_1 = 0.0244, wR_2 = 0.0637
Final R indexes [all data]	R_1 = 0.0245, wR_2 = 0.0638
Largest diff. peak/hole / e Å^{-3}	0.17/-0.31
Flack parameter	0.057(4)

Supplementary References

1. J. R. C. Muniz, A. L. B. Ambrosio, H. S. Selistre-de-Araujo, M. R. Cominetti, A. M. Moura-da-Silva, G. Oliva, R. C. Garratt, D. H. F. Souza, The three-dimensional structure of bothropasin, the main hemorrhagic factor from Bothrops jararaca venom: Insights for a new classification of snake venom metalloprotease subgroups. *Toxicon* **52**, 807–816 (2008).

2. D. Zhang, I. Botos, F. X. Gomis-Rüth, R. Doll, C. Blood, F. G. Njoroge, J. W. Fox, W. Bode, E. F. Meyer, Structural interaction of natural and synthetic inhibitors with the venom metalloproteinase, atrolysin C (form d). *Proceedings of the National Academy of Sciences* **91**, 8447–8451 (1994).

3. T. Lingott, C. Schleberger, J. M. Gutiérrez, I. Merfort, High-Resolution Crystal Structure of the Snake Venom Metalloproteinase BaP1 Complexed with a Peptidomimetic: Insight into Inhibitor Binding. *Biochemistry* **48**, 6166–6174 (2009).

4. L. E. Misson Mindrebo, J. T. Mindrebo, Q. Tran, M. C. Wilkinson, J. M. Smith, M. Verma, N. R. Casewell, G. C. Lander, J. G. Jardine, Importance of the Cysteine-Rich Domain of Snake Venom Prothrombin Activators: Insights Gained from Synthetic Neutralizing Antibodies. *Toxins* **16**, 361 (2024).

5. S. Takeda, T. Igarashi, H. Mori, Crystal structure of RVV-X: An example of evolutionary gain of specificity by ADAM proteinases. *FEBS Letters* **581**, 5859–5864 (2007).

6. G. Jones, P. Willett, R. C. Glen, A. R. Leach, R. Taylor, Development and validation of a genetic algorithm for flexible docking11Edited by F. E. Cohen. *Journal of Molecular Biology* **267**, 727–748 (1997).

7. Y. Li, L. Han, Z. Liu, R. Wang, Comparative Assessment of Scoring Functions on an Updated Benchmark: 2. Evaluation Methods and General Results. *J. Chem. Inf. Model.* **54**, 1717–1736 (2014).

8. K. Lindorff-Larsen, S. Piana, K. Palmo, P. Maragakis, J. L. Klepeis, R. O. Dror, D. E. Shaw, Improved side-chain torsion potentials for the Amber ff99SB protein force field. *Proteins: Structure, Function, and Bioinformatics* **78**, 1950–1958 (2010).

9. G. Bussi, D. Donadio, M. Parrinello, Canonical sampling through velocity rescaling. *The Journal of Chemical Physics* **126**, 014101 (2007).

10. M. Parrinello, A. Rahman, Polymorphic transitions in single crystals: A new molecular dynamics method. *Journal of Applied Physics* **52**, 7182–7190 (1981).

11. R. T. McGibbon, K. A. Beauchamp, M. P. Harrigan, C. Klein, J. M. Swails, C. X. Hernández, C. R. Schwantes, L.-P. Wang, T. J. Lane, V. S. Pande, MDTraj: A Modern Open Library for the Analysis of Molecular Dynamics Trajectories. *Biophysical Journal* **109**, 1528–1532 (2015).

12. F. Pedregosa, G. Varoquaux, A. Gramfort, V. Michel, B. Thirion, O. Grisel, M. Blondel, P. Prettenhofer, R. Weiss, V. Dubourg, J. Vanderplas, A. Passos, D. Cournapeau, M. Brucher, M. Perrot, É. Duchesnay, Scikit-learn: Machine Learning in Python. *J. Mach. Learn. Res.* **12**, 2825–2830 (2011).

13. M. M. Badawi, A. A. F. Alla, S. S. Alam, W. A. Mohamed, D. A. N.-E. Osman, S. A. A. A. Ali, E. M. E. Ahmed, A. A. Adam, R. O. Abdullah, M. A. Salih, Immunoinformatics Predication and in silico Modeling of Epitope-Based Peptide Vaccine Against virulent Newcastle Disease Viruses. *American Journal of Infectious Diseases and Microbiology* **4**, 61–71 (2016).

14. F. Sievers, A. Wilm, D. Dineen, T. J. Gibson, K. Karplus, W. Li, R. Lopez, H. McWilliam, M. Remmert, J. Söding, J. D. Thompson, D. G. Higgins, Fast, scalable generation of high-quality protein multiple sequence alignments using Clustal Omega. *Molecular Systems Biology* **7**, MSB201175 (2011).

15. A. Waterhouse, M. Bertoni, S. Bienert, G. Studer, G. Tauriello, R. Gumienny, F. T. Heer, T. A. P. de Beer, C. Rempfer, L. Bordoli, R. Lepore, T. Schwede, SWISS-MODEL: homology modelling of protein structures and complexes. *Nucleic Acids Research* **46**, W296–W303 (2018).

16. G. Studer, C. Rempfer, A. M. Waterhouse, R. Gumienny, J. Haas, T. Schwede, QMEANDisCo—distance constraints applied on model quality estimation. *Bioinformatics* **36**, 1765–1771 (2020).

17. A. J. Campbell, M. L. Lamb, D. Joseph-McCarthy, Ensemble-Based Docking Using Biased Molecular Dynamics. *J. Chem. Inf. Model.* **54**, 2127–2138 (2014).

18. B. Lovejoy, A. R. Welch, S. Carr, C. Luong, C. Broka, R. T. Hendricks, J. A. Campbell, K. A. M. Walker, R. Martin, H. Van Wart, M. F. Browner, Crystal structures of MMP-1 and -13 reveal the structural basis for selectivity of collagenase inhibitors. *Nature Structural Biology* **6**, 217–221 (1999).

19. J. Y. Choi, E. Chung, Molecular Dynamics Simulations of Matrix Metalloproteinase 13 and the Analysis of the Specificity Loop and the S1′−Site. *International Journal of Molecular Sciences* **24**, 10577 (2023).
